# Librarian of Alexandria: A Modular Chemical Data Extraction Pipeline to Compare LLM Performance

**DOI:** 10.1021/acs.jcim.6c00374

**Published:** 2026-06-09

**Authors:** Morgan Grougan, Janya Subasinghe, Mark A. Hix, Alice R. Walker

**Affiliations:** Department of Chemistry, 2954 Wayne State University, Detroit, Michigan 48202, United States

## Abstract

Dataset creation is a critical component of predictive machine learning technology. In the field of chemistry, large experimental datasets are scarce, especially for niche chemical properties and topics, and their creation is cumbersome and time-consuming when performed manually. Here, we present Librarian of Alexandria (LoA), an open-source and lightweight framework for testing large-language models (LLMs) on the task of generating large datasets via direct extraction from scientific literature. LoA is available on GitHub along with example inputs, outputs, Docker container, and a Colab-friendly Jupyter Notebook. LoA has the chosen LLM(s) check the relevance of a research paper and perform data extraction. Two separate or identical LLMs may be independently and modularly specified by the end-user for these separate tasks. LoA can be easily updated via simplified user incorporation of the latest available LLMs. We compare several LLMs for both relevance and extraction functions and automate the collection of research papers for several popular chemical journals providing open access. LoA provides a much-needed testing environment which can be used to develop LLMs capable of assembling enormous chemical datasets with minimal effort on the part of the scientist. The best models we were able to test achieved ∼95% accuracy, which is sufficient for the purposes of training predictive machine learning models.

## Graphical abstract

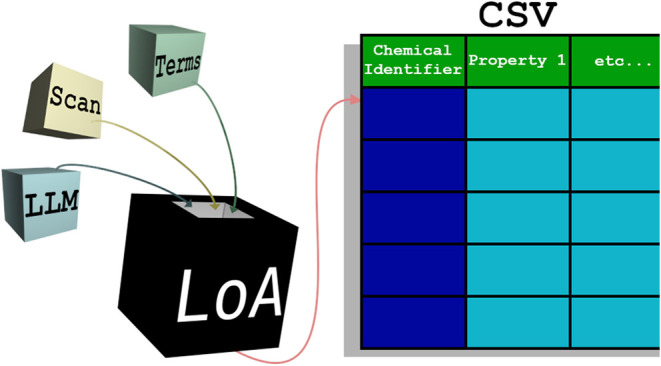

## Introduction

Chemistry research continually seeks to expand human knowledge, which has led to an exponential increase in published chemical literature. The number of publications doubles roughly every 15 years, continuously increasing the amount of information that researchers must identify, parse, and build on.^1^ The magnitude of this challenge has been recognized since at least the turn of the millennium, leading to the development of many automated tools to extract, distill, or organize chemical information.^2^ These tools can be categorized along the spectrum between purely linguistic heuristics^3,4^ and large language models (LLM),^5–11^ with hybrid approaches in the middle^12,13^ (see Table 1).

**Table 1:** Representative Automated Tools for Chemical Information Extraction, Categorized by Methodological Approach

category	representative tools	typical inputs	outputs/scope
Heuristic/Rule-based	ChemDataExtractor;^3^ label-free extraction^4^	Scientific text (PDF, HTML)	Structured entities (e.g., compounds, properties); high precision, domain-specific rules
Hybrid (rules + ML)	OpenChemIE;^12^ Shon et al.^13^	Scientific text, sometimes annotated corpora	Relation extraction and structured knowledge; balances generalization and interpretability
LLM-based	Vaucher et al.;^5^ Polak et al.;^6^ Lee et al.;^7^ Kumar et al.;^8^ Gilligan et al.;^9^ Dagdelen et al.;^10^ Chen et al.^11^	Natural language prompts; scientific text; multimodal inputs (in some cases)	Flexible extraction, reasoning, and autonomous workflows; adaptable but variable reliability

Schilling and Wilhelmi et al. provide a comprehensive review of data extraction methods, highlighting both the promises and the challenges of LLM-driven extraction in chemistry.^14^ Heuristic-based tools remain widely used due to lower hardware costs.^15^ LLMs excel at parsing nuanced language and can infer relationships that may not be explicitly stated, yet their probabilistic text generation may lead to “hallucination” of results.^16^ In most cases, researchers prefer to sacrifice some coverage (missing data) rather than risk hallucinated values, especially when extracting numerical data for downstream machine learning. This is likely why heuristic-based approaches have remained the mainstay over LLM-based methods, despite their difficulty of use.

Before the advent of contemporary large context transformer models, question answering (QA) systems often relied on fine-tuned bidirectional encoder representations from transformers (BERT) variants specifically tailored to scientific domains, such as SciBERT^17^ or BioBERT.^18^ These models aimed to identify relevant passages or tokens and then extract short, factual answers. Such BERT-based QA pipelines and their reliance on targeted retrieval are a common ancestor of several modern approaches, including both the retrieval-augmented generation (RAG) method and the Librarian of Alexandria (LoA) method, which we introduce here. Traditional QA systems delivered strong accuracy on limited spans of text, but struggled to parse entire papers or large property tables. Vector databases provided faster information retrieval from large sets of documents, enabling LLM chat interactions to deliver factual responses on demand.^19^

RAG is a strategy for enriching chat interactions with LLMs, in which an external textual context is gathered from some repository and is appended to the LLM prompt, providing more specialized or up-to-date knowledge in response to conversational user queries.^20^ Although the advantages of RAG include an expanded context window and fewer hallucinations, it requires additional overhead for indexing and embeddings maintenance. Our work focuses on large-scale chemical data extraction rather than short interactive dialogues, utilizing prompt engineering and taking advantage of the higher token limits offered by modern models, resulting in different application options.

Here we present the “Librarian of Alexandria” (LoA), an open-source tool designed to test modern LLMs on the task of automated large-scale search and extraction of chemical property data from published literature (see Figure 1). We include a GitHub repository with Docker container that includes examples and a Colab-friendly Jupyter notebook for ease of use and implementation. It may be described as a specialized RAG pipeline; however, it diverges from typical RAG usage in purpose and design. LoA aims at testing modern and future LLMs on their ability to obtain high-recall coverage and ease of dataset construction, rather than rapid response QA. Our approach prioritizes the extraction of large amounts of data in a stable CSV or database, sacrificing the convenience of the chat-like interaction *ad hoc*. LoA incorporates an external retrieval step from a user-specified set of chemical publications by scraping and converting text from open-access chemical journals, repositories, or other APIs. Using a two-phase LLM pipeline, it filters papers by relevance, parses the full text of these papers into machine-readable formats, and runs an extraction model to obtain the desired chemical properties. LoA then validates and filters the extracted data to create a highly ordered dataset (such as a CSV file) for subsequent machine learning operations. LoA can be viewed as an architectural choice within the broader space of retrieval-augmented and agentic-inspired systems,^21^ which interleave retrieval, transformation, and reasoning over external data sources. Unlike fully agentic approaches that rely on dynamic planning and tool selection, LoA adopts a structured, pipeline-based design that emphasizes modularity and reproducibility. Given that agentic retrieval systems remain an emerging and fragmented paradigm with limited standardization and evaluation practices,^20,22^ LoA-style pipelines provide a practical and accessible framework for systematically exploring these design patterns.

**Figure 1:**
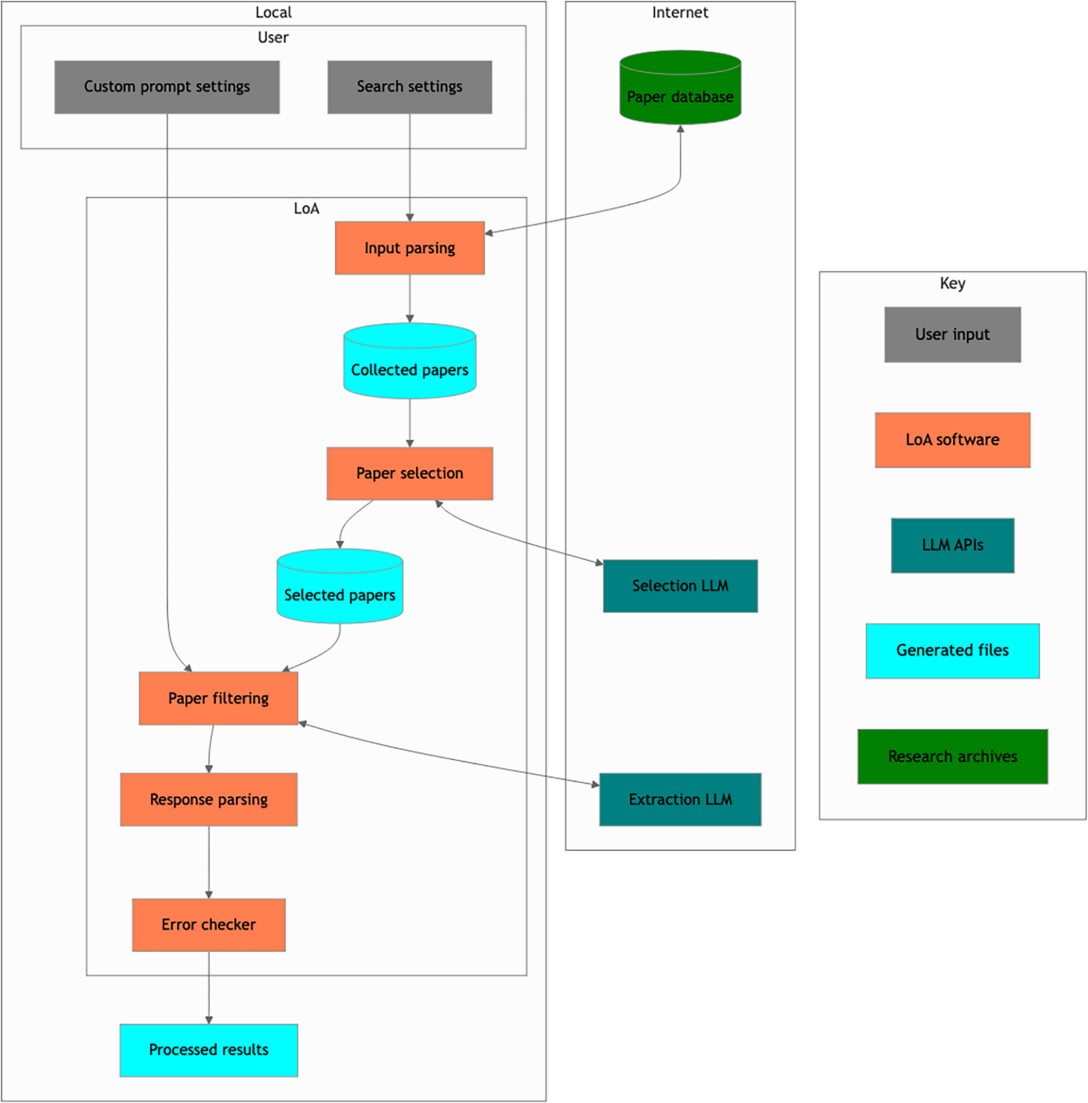
Overall workflow of LoA, from paper scraping to extraction. Directional arrows indicate the forward flow of information through our code, while bidirectional arrows indicate external queries which provide information back.

The design principles of LoA emphasize flexibility and future-proofing, allowing the end user to select different LLMs for each phase and enabling comparisons between language models with minimal extra coding. Because LoA is built with widely used open-source libraries, it can be adapted to more advanced retrieval or extraction modules with minimal changes.

Within the broader landscape of automated chemical text mining, LoA distinguishes itself not merely by integrating existing components, but by formalizing a modular, LLM-centric pipeline architecture tailored for large-scale dataset construction rather than interactive query answering. In contrast to conventional RAG or fully agentic systems, LoA decouples relevance assessment and extraction into independently configurable LLM stages, enabling systematic evaluation of model behavior across subtasks while maintaining a structured and reproducible workflow. A key methodological contribution is the programmatic construction of prompts from user-defined schemas-including explicit constraints, generated examples, and optional domain heuristics-which reframes extraction as a semiformal specification rather than *ad hoc* prompt engineering.

These design choices allow LoA to leverage high-context reasoning models without requiring fine-tuning, vector databases, or complex preprocessing pipelines, thereby reducing the demand for specialized coding, high-end hardware, and multistage system design. In practice, this simplifies and accelerates large-scale dataset creation from the literature while preserving user control and extensibility, including the ability to incorporate domain knowledge and to interchange LLMs on the fly. As a result, LoA serves not only as a practical extraction tool, but as a reproducible and extensible framework for benchmarking and exploring LLM-driven data extraction, aligning with emerging agentic retrieval paradigms while retaining transparency and controllability.

## Methods

### Paper Scraping

LoA accepts user-provided search term lists and uses these to scrape research papers from various sources. “Definite” search terms are used in every search, while “maybe” search terms are iterated. This produces a combinatorial set of search terms, often leading to more relevant results (Figure 2). The user is also able to control the maximum number of papers to fetch per combination of search terms per database, ensuring that database access limits are not exceeded.

**Figure 2:**
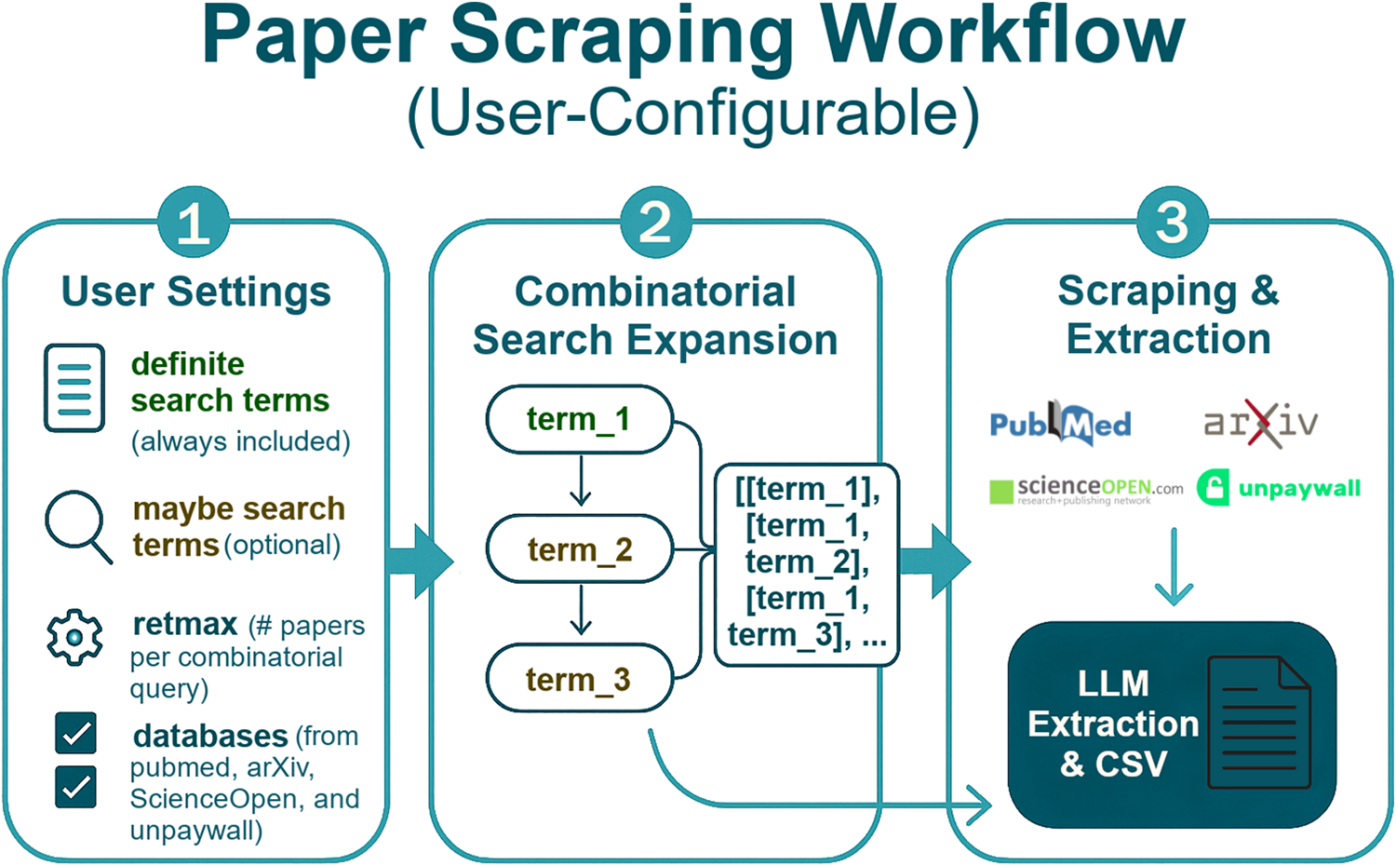
Settings the user may define when performing scraping, and the effects they have on the resulting search­(es).

The current release of LoA includes scraping capabilities for arXiv, chemrXiv, PubMed, ScienceOpen, and UnPayWall, each with their own set of specific requirements and behaviors (see Figure 2). While arXiv, chemrXiv, and UnPayWall return PDFs which must be processed through optical character recognition (OCR), PubMed returns XML-formatted papers which may be processed to plaintext with perfect fidelity. Additionally, while the others provide an API for scraping, ScienceOpen within LoA requires the Chrome Internet browser to be installed on the user’s machine for scraping as there is no direct API available. ScienceOpen does not forbid scraping of their Web site, and hosts content under a CC-BY 4.0 license. Each repository requires consideration of access rate limits to avoid lockouts, so we encoded a simple handling process in accordance with each Web site’s access rules as per their robots.txt files and terms and conditions. However, we determined that the extraction model size required to achieve reasonable competence is not fast enough to meet this limit on our testing hardware. It should be noted that LoA can be provided with a specific folder containing documents via the “batch_extract­()” function. As well, to do repeated tests on the same papers that have already been scraped by LoA, simply remove the portion of the job script for scraping, but leave in the search terms, and LoA will process all previously downloaded documents. This way the scraping function can be used to establish a database, and then process it afterward with various models and settings for comparison.

### Document Parsing

The document parsing in LoA is designed around the type of document that is obtained by the various scraping functions. We process the XML files that come from PubMed Central directly. The other repositories return PDFs which we pass through a sophisticated Optical Character Recognition (OCR) system built on the Unstructured Python library, making it possible for LoA to handle the file types shown in Table 2. We use the high resolution (hi-res) setting offered as it results in a significant reduction of character recognition errors at the expense of speed. The Unstructured developers state that the high-res OCR method has trouble with papers containing multiple columns, so we revert to low-res mode when multiple columns are identified in a paper.

**Table 2:** File Types by Category

category	file types
CSV	.csv
Email	.eml, .msg, .p7s
EPUB	.epub
Excel	.xls, .xlsx
HTML	.html
Image	.bmp, .heic, .jpeg, .png, .tiff
Markdown	.md
Org Mode	.org
Open Office	.odt
PDF	.pdf
Plain text	.txt
PowerPoint	.ppt, .pptx
reStructured Text	.rst
Rich Text	.rtf
TSV	.tsv
Word	.doc, .docx
XML	.xml

Scraped papers and their corresponding processed plaintexts are stored in respective subdirectories during execution, and the proper reference is included in the entry in the final CSV. This allows the user to select random entries for manual validation if they desire and may also provide guidance in diagnosing potential problems with search terms, LLM choices, or other parameters.

### LLM Provider

In projects involving LLMs it often becomes necessary to write or include code for every type of model you would like to run, due to the widely varied architecture of LLMs in the present day. A core aim of this project was to enable the swapping of LLMs with ease. In keeping with our design principle of flexibility and to enable easy swapping of LLMs, we use Ollama as our backend for running inference with various LLMs as it is consistently updated to use the latest LLMs (currently 232 supported models). This has allowed us to quickly swap models out to test and compare them against each other during the development of LoA, and ensures that LoA can incorporate improved LLM models upon release.

We also designed LoA to run unprivileged to allow users without administrator access to use it. This includes checks for system privileges to determine whether Ollama can be installed with or without higher-level access, or if a locally downloaded binary must be obtained and run as an isolated subprocess within LoA. This approach enables the usage of LoA by any user on any Linux distribution. We believe that this design approach will allow researchers with access to a distributed computing systems to apply LoA for use with larger and more accurate models than could reasonably be obtained and used on consumer-grade desktop computing systems.

### Prompt Engineering

Multiple sources of text are used to create the final prompt given to the LLM. The user-provided section aims to clarify and specify the type of information desired from their perspective on the scientific task. Users may easily and quickly add short statements to help avoid common pitfalls. For example, if, in a task designed to extract acid dissociation constants (p*K*_a_), the model often confuses protein kinase A (PKA) with p*K*_a_, the user may add a statement that the model can incorporate into its reasoning to avoid the issue. In this way, a specific job can be quickly iterated upon to improve understanding by the LLMs (see Figure 3).

**Figure 3:**
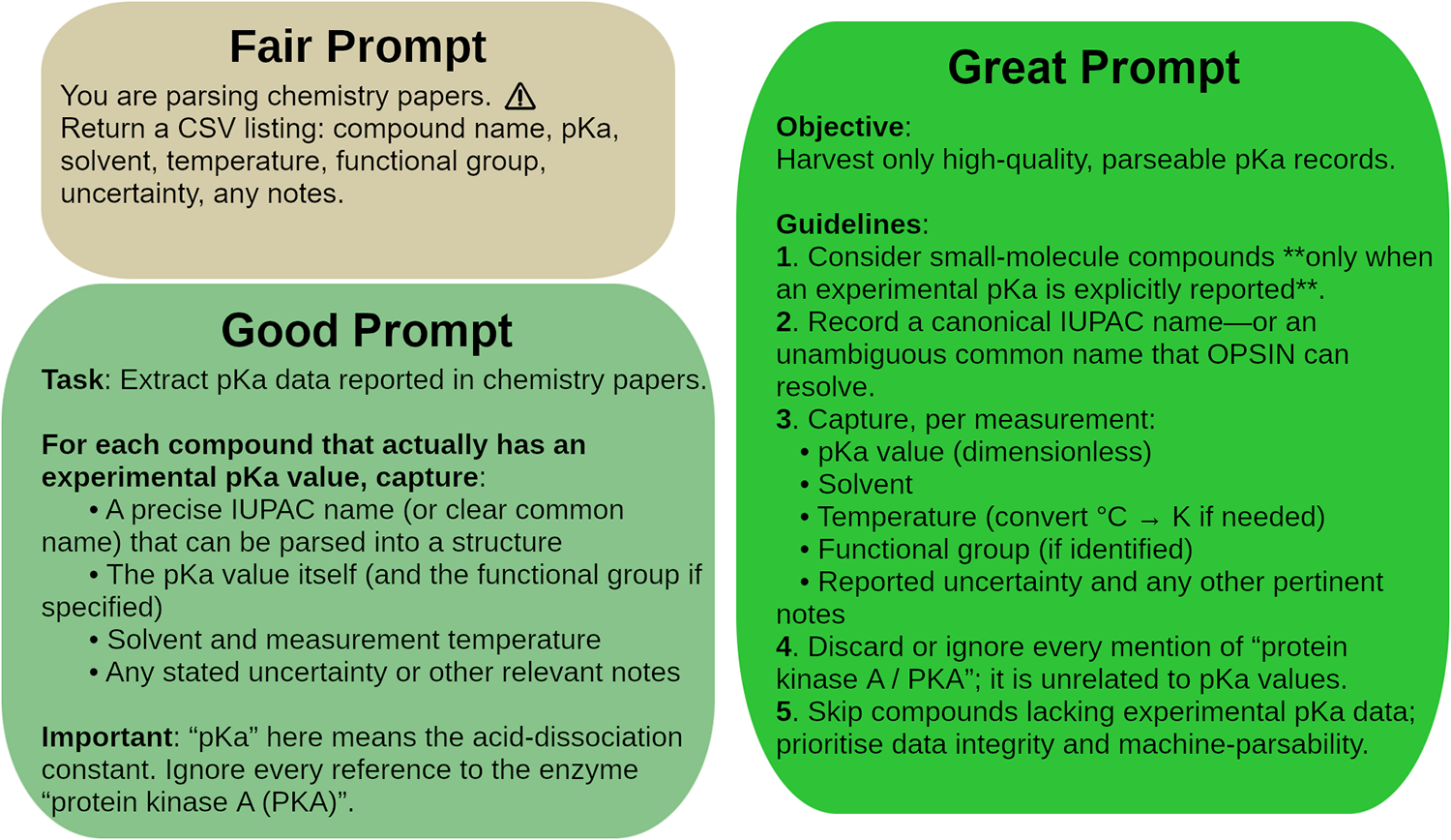
Examples of prompts of different quality. The fair prompt shows little to no foresight of the problem, includes problematic characters, and simply restates the information present in the schema. The good prompt provides more depth and includes things the model may need to consider when providing a response. The great prompt adds specific guidelines on the units desired, provides chemical intuition, and uses quality positive language to encourage positive results.

The user also defines expectations for the aggregated outputs by defining columns to be used in the CSV along with datatypes (integer, decimal, text string, boolean, etc.) and ranges or other constraints. The user is also able to define a description of the property for each column in a manner similar to the overall description of the problem, which can offer more detailed insights or limitations on a given column. This information is used in the construction of the final prompt (see Figure 4).

**Figure 4:**
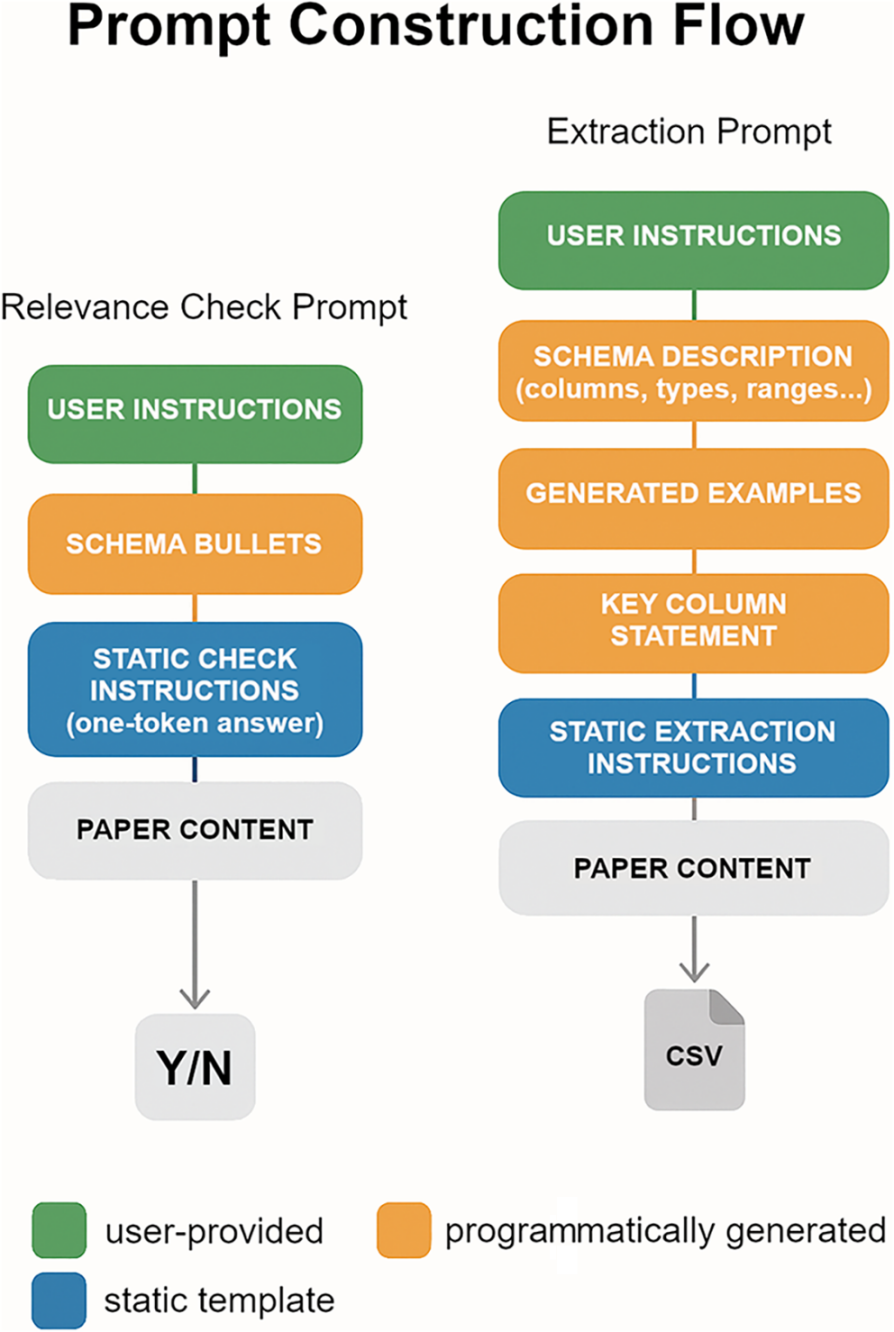
Anatomy of the constructed prompt, simplified to show the purpose of each section, as well as what information comes from the user, and what we generate automatically using the schema.

LoA converts all the column information into natural language for the LLM, then generates examples with varying numbers of entries to show the model the exact format its output should be in. The user is able to designate “key columns” specifying what information must be included and unique for a result to be valid. For example, the name of a chemical in an entry should be a key column because logically a chemical property cannot exist without a chemical, and because a single chemical cannot have two values for the same property. Because every job may have different requirements for the final output, it is important that we programatically generate examples to guide the model in the production of this output.

The remainder of the prompt is primarily a static set of instructions, with a section containing the raw paper information that is updated for each paper processed. These instructions were iteratively modified during development to get the best results with many different models and architectures, and they are primarily designed to provide a high-level explanation to the model regarding its task. In addition, they provide clear instructions on the desired output format which are general enough to align with any reasonable format the user has defined. Interestingly, we found that clearly instructing the model that invalid or improperly formatted responses waste time and resources helped to ensure that the model adheres to the desired output format.

In addition to the extraction prompt, we construct a similar simplified prompt to check for relevance of the paper to the user’s stated goals. This prompt still provides the model with information regarding the user’s desired output; however, the expected response from the model is a simple Boolean token. We further include instructions in the prompt that any extraneous response outside of the Boolean token is considered a waste, and the paper is discarded.

### Response Parsing

We created our own response parser based on the most common types of responses and subsequent errors within. Many models provide responses in a CSV format even when instructed to use JSON, which may be due to the way in which a CSV entry may more closely resemble natural language (a comma-separated list of items). As a result of this more “natural” format expectation, subsequent response parsing was considerably easier.

Parsing begins by ignoring any “thought sections” generated by the use of modern reasoning models. We then parse each line of response individually, as the CSV format necessarily treats each entry as its own line of text. This approach allows us to immediately discard any lines that have the incorrect number of columns, followed by lines which do not meet the data-type requirements, and finally remaining range and other user-defined value restrictions (i.e., “SMILES string must be a valid chemical structure”) This enables us to filter out any erroneous data and provides the user with more control for their specific use case.

LoA supports six chemistry-aware operating modes: small molecule, peptide, protein, reaction, polymer, and general. In small molecule mode, the model is prompted to return parseable molecular identifiers (preferably SMILES, or a common name that can be resolved). In protein and peptide modes, LoA expects amino-acid sequence representations (e.g., FASTA-style strings) or resolvable names; peptide handling remains sequence-centric and is most reliable for standard residue representations. In polymer mode, LoA validates polymer outputs as BigSMILES. In reaction mode, LoA validates separate reactant and product fields as molecular strings. In general mode, no built-in target column is injected, but per-column validation can still be enabled through schema-level validation settings. Validation is performed by first attempting direct parsing/conversion into machine-readable molecular representations, and when needed, by fallback name resolution through tools such as RDKit/Cirpy/PubChem-based lookups, ensuring extracted entities are suitable for downstream ML workflows.

### Multimodal Inference

Recently there has been a large improvement in multimodal models’ ability to reason scientifically. As such, we believed it imperative to include support for these types of models. In our testing, we found that multimodal models were able to reason over scientific figures to extract numerical values not present in the text. However, they are not able to determine the SMILES string or even IUPAC name of compounds from including the image in a multimodal prompt alone. So, we attached DECIMER^23–25^ to our code, allowing for automated recognition and parsing of chemical figures into SMILES strings.

Our code also attempts to place the parsed SMILES in the proper location within the text, adjacent to a figure identifier. This allows the model to see the figure as embeddings, know where it is meant to go within the text, and know what chemicals are referenced in the figures. Taken together, this is incredibly powerful for meaningfully parsing chemical figures into usable data. Not all modern models are capable of parsing images in this way (i.e., text-only LLMs, such as deepseek-v3.2, lack native image-processing capability, whereas multimodal models with integrated vision encoders, such as recent GPT-4-class or Gemini models, can process images), so we have made the use of this feature a mode which can be toggled on and off. Testing of this feature was performed, and is detailed in our results.

### Miscellaneous Features

There are a number of small functions and features included in LoA that contribute to its improved functionality, including data-type verification, model-updating, and DOI handling for relevant papers. For complete open-access code, please refer to the stable release (https://doi.org/10.5281/zenodo.19895268) for the code used in this paper and/or our maintained GitHub repository for the latest version of our code and Docker container. (https://github.com/arwalkerlab/LoA-Stable)

## Results and Discussion

### Check Model Comparison

To determine the best model among the current model zoo for use in checking the relevance of scraped papers, we ran jobs with different models as the ’check model’ on different tasks and quantified their performance by manually reviewing the results and labeling them according to true positive (TP), true negative (TN), false positive (FP), and false negative (FN) (Figure 5). A result is considered to be true positive if there is both information present to be extracted and the check model recognizes this; if it does not recognize it as present, it is considered false negative. Similarly, if a given paper has no relevant information and the model recognizes this, it is true negative; otherwise, it is false positive.

**Figure 5:**
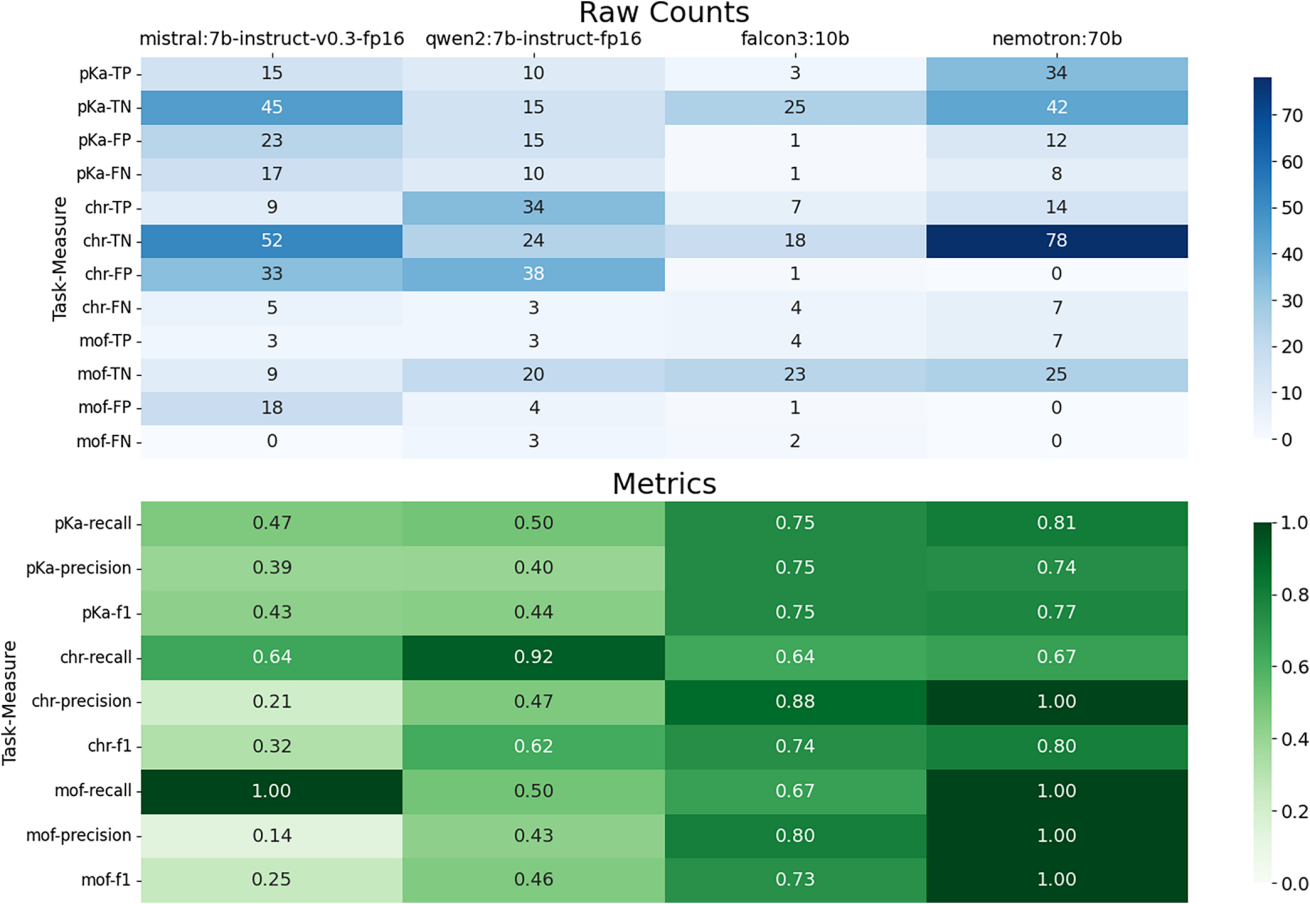
Performance of models on the task of checking whether or not the paper is relevant, accross three user-defined tasks. Computed recall, precision, and f1 scores are provided to gauge performance.

By quantifying the performance of the check model in this way, we can calculate precision, recall, and overall F_1_ score for models on the check prompt for easy comparison.

Precision is defined as the ratio of true positives to the number of predicted positives

$$\mathrm{precision}=\frac{\mathrm{TP}}{\mathrm{TP}+\mathrm{FP}}$$

Recall is defined as the number of true positives divided by the number of actual positives

$$\mathrm{recall}=\frac{\mathrm{TP}}{\mathrm{TP}+\mathrm{FN}}$$

Finally, *F*_1_ score is defined as the harmonic mean of precision and recall

$$F_{1}=\frac{\mathrm{precision}\cdot \mathrm{recall}}{\mathrm{precision}+\mathrm{recall}}$$

We had to limit the number of results checked per model in some cases, as manually verifying thousands of results would be too time-consuming for these results to be relevant by the time of publication. We also qualify on the basis of the time it takes to extract, as it is possible that a slight decrease in model accuracy may be worth the improvement in overall computational speed (see Figure 5).

### Discussion of Best Practices

There are a number of parameters under the complete control of the user, and consequently there are some “best practices” when setting up LoA to run a job. There are many small things the user can do to improve the results. Including common mistakes in the prompt may help the model avoid them, and is a step users should consider to maximize success using LoA. When starting a new job, it is recommended that the user run on a few entries at a time until they identify a mistake, then modify the prompt to address it, and rerun. While this may seem tedious and counterintuitive to the goal of automation, it can be done very quickly, and is highly effective due to the similarity of issues which may arise from a common domain. The previously mentioned issue of mistaking protein kinase A with p*K*_a_ is easily solved by explaining this difference to the model in the user-entry portion of the prompt. Our hard-coded instructions seek to eliminate many of the domain-agnostic issues, but for certain areas of research, specific issues will arise which can be overcome simply by recognizing them and preempting them in the initial input.

LoA is available for use in both interactive and batch mode, with the latter requiring only modifications to an initial input file. Details on these methods are provided on the LoA-stable GitHub page and within the Google Colab notebook provided there. We also tested the use of several popular large language models to generate these input files automatically given an explanation of the goals; however, these were often too general and should not be used without user scrutiny.

### Testing Multimodal Performance

To demonstrate the power of multimodal models at this task, we obtained an experimental dataset of the photophysical properties of chromophores.^26^ This database was painstakingly constructed by humans manually reviewing 1358 research papers for relevant information. Our goal was to have LoA reconstruct a portion of this database from these same articles, leveraging multimodal parsing. We were able to obtain 1242 of the original 1358 articles and run extraction over all 12 fields present in the original dataset via LoA. This constitutes approximately 150,000 individual fields for LoA to reconstruct via extraction from the original papers. The remaining papers were inaccessible due to our university lacking the required academic journal subscriptions for manual download. By starting with an existing database, we were able to quantify the results on the basis of numerical accuracy. We used OpenAI’s o3 model to produce these results, which are highly accurate for all properties except quantum yield (Figure 6, see Table 3, S1–S18).

**Figure 6:**
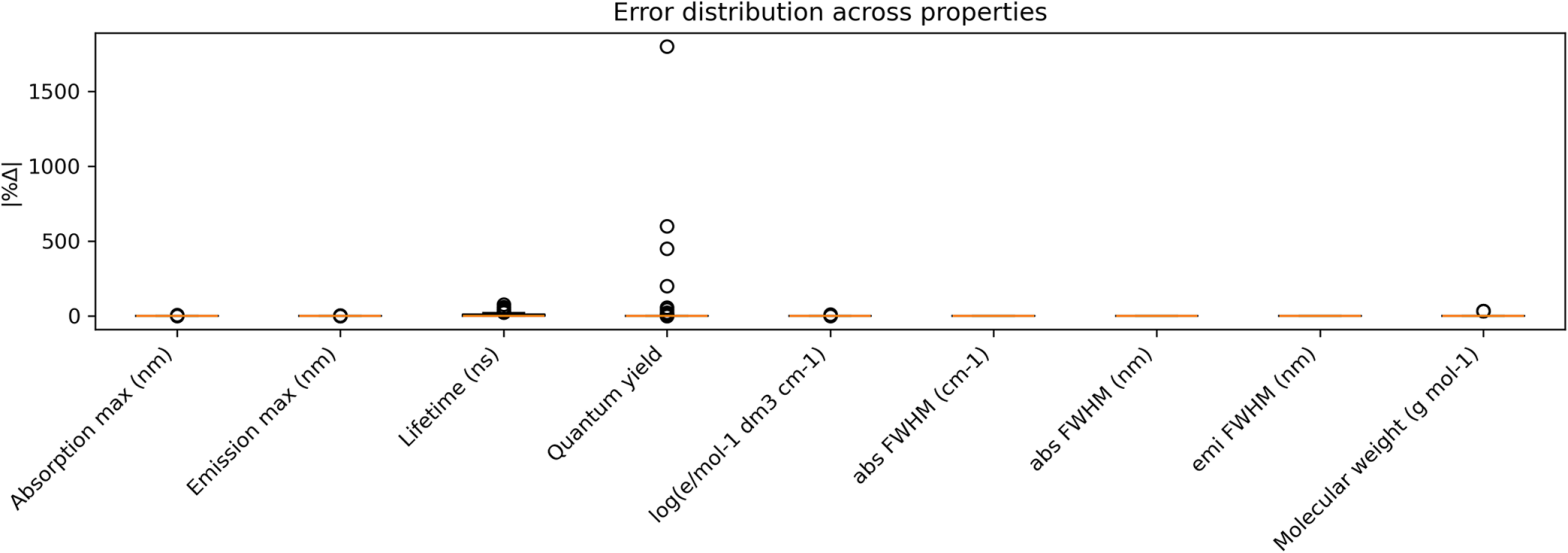
Distribution of errors by percentage across all types of results. All properties aside from Quantum Yield show nearly perfect error distributions.

**Table 3:** Photophysical Extraction Results Summary

property	accuracy (%)	mean |Δ|%	missed	extra	
absorption max (nm)	99.91	0.09	5	0	
emission max (nm)	99.94	0.06	4	2	
lifetime (ns)	91.03	8.97	5	3	
quantum yield	69.53	30.47	0	4	
log(ε/mol^–1^ dm^3^ cm^–1^)	99.72	0.28	0	5	
Abs FWHM (cm^–1^)	100.00	0.00	0	0	
Emi FWHM (cm^–1^)	—	—	0	0	
Abs FWHM (nm)	100.00	0.00	24	0	
Emi FWHM (nm)	100.00	0.00	39	0	
molecular weight (g mol^–1^)	98.20	1.80	29	0	
**average coverage ratio (incl.** zero-extractions)					0.014
**average coverage ratio (excl.** zero-extractions)					0.913
**overall accuracy**					95.37%

A likely component of this increased error in quantum yield is the limited numerical precision to which it can be reported. Even a small error results in a large percentage difference. It also may be that this value is often reported in images, leading to errors due to the multimodality failing to correctly read the integers. It is also possible that the subset of papers we processed reported quantum yield in different ways (for example, as a percentage rather than a decimal). There are many more possibilities, but the takeaway is that the method seems to accurately extract some properties better than others.

**Table 4:** Accuracy Percentage by Numeric Property, Defined as 100 – (100 × Mean Relative Error)^a^

property	*n*_compared_	accuracy (%)
number-average molecular weight (Mn)	149	57.75
weight-average molecular weight (Mw)	80	41.35
dispersity (*D*)	151	87.34
degree of polymerization (*N*)	13	100.00
temperature (*T*)	79	–175.81
measured temperature (T_meas)	54	23.53
volume fraction of block 1 (f1)	176	95.51
total volume fraction of block 1 (f_tot1)	164	89.30
interaction parameter for block 1 (w1)	23	97.65
density of block 1 (rho1)	65	83.47
volume fraction of block 2 (f2)	176	95.20
total volume fraction of block 2 (f_tot2)	153	96.34
interaction parameter for block 2 (w2)	23	76.40
density of block 2 (rho2)	65	74.81

a Values are defined as per the original Block Copolymer Phase Behavior Database.^27^

There is also an issue of coverage: some properties were extracted in as high quantities as the original, some were extracted more than the original, and a few were extracted far less than the original. In this experiment, the notable losses came from all of the full-width at half-maximum (FWHM) properties. To extract this property, the extractor (human being or LoA) must measure the distance between the lines of the spectrum at half of the peak value. This is an intrinsically involved process requiring a decent amount of measurement. Were a human to extract this data, they would likely use software to measure the distance accurately. However, the multimodal model must “eyeball” the value without any measurement tools. As such, it seems that either the model is more likely to shy away from providing a value at all when it is likely to make a mistake, or that it simply did not recognize that it had access to this information. We find the latter to be more likely, though the model might argue otherwise if asked. It is likely that an improvement to the prompt could correct this behavior, but due to the monetary cost of running the o3 model via API, we are limited to the results we have from this run. Scatter plots and histograms of all results’ accuracy and coverage can be found in the SI.

It is also worth mentioning that coverage is only 1.4% when you consider failed papers. There were a few papers which our method failed to extract any data from, which contained a huge number of data points to be extracted. It seems that the model struggles when the information density is too large. If you exclude these cases, the coverage is optimal, sitting at 91.3%. This is a monumental disparity, and is a notable area for future improvement.

To further qualify LoA’s ability to be extrapolated to other types of information extraction, the Block Copolymer Phase Behavior Database was utilized as a source of ground truth which contains reference to the papers which the information came from.^27^ We selected 31 papers randomly from the database, with our target being to get over 500 total rows to extract. Then, due to the extensive amount of sparse data in the database, we removed all columns for which there were less than 20 instances of data within the sampled reference dataset. We then used the remaining columns as our target for LoA. This extraction shows that most properties achieve accuracy levels useful for downstream machine learning (Table 4). However, temperature related values do not. This is likely due to differences in how temperature is reported, i.e., varying units or symbolic representations. Molecular weight was challenging for a similar reason: Clustering the data via scatter plot shows a cluster of points with a similar trend to the main line 3 orders of magnitude lower than expected, indicating the issue arises from reporting in either Daltons/atomic mass units or kilodaltons. There is also a cluster of points with a reported molecular weight of 0, which could easily be filtered out in downstream applications. As such, we expect that minor adjustments to our prompt to specify desired units or minimal postcollection data cleaning could solve these issues (Figure 7).

**Figure 7:**
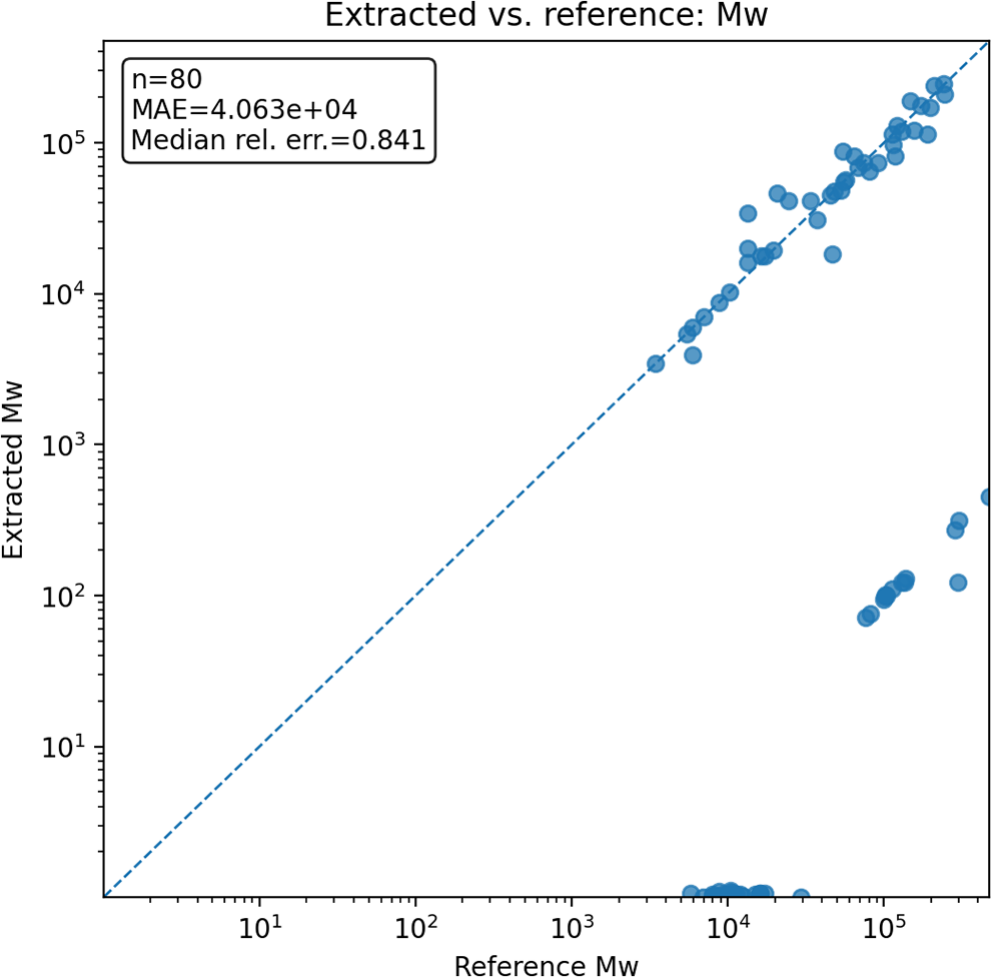
Expected versus predicted molecular weight for our extraction of polymer information.

### Full Photophysical Dataset

To showcase the functionality of LoA, we include the final output from a full run. We focused on building a dataset of spectroscopic measurements, specifically of small molecules with valid structures and experimental photophysical measurements while avoiding e.g., fluorescent proteins. After testing several prompts, we used:

Extract information about small molecules with photophysical data from the provided research paper according to these guidelines:

1. Include any molecule (often called a chromophore, fluorophore, dye, probe, etc.) for which absorption, emission, quantum yield, or other photophysical properties are reported. We are not strictly limited to the term “chromophore.”
2. Only include data if the chemical name is parseable (i.e., a valid IUPAC name or a well-known common/trade name typically resolvable by name-to-structure libraries). Exclude local paper labels (e.g., “Compound 1a”, “V-3a”, etc.) when no parseable name is provided. In general, if you are unsure if a name is parsable, if it is exceedingly short, it is not parsable. However, though most papers use names like this to avoid rewriting the full iupac name over and over, they often do provide the full iupac name in the paper somewhere.
3. For numeric values:
  - Absorption and emission wavelengths range from 200 to 1100 nm.
  - Quantum yield is between 0 and 1.
  - If only a range is provided, take the midpoint of that range (do not provide min-max ranges).
  - Lifetimes may be reported in ns or μs; store them as a float in ns if possible.
4. Exclude data for proteins, large biomolecules, or polymers unless a specific small-molecule chromophore or moiety is well-defined and parseable.
5. Include any additional relevant photophysical measurements, if available (e.g., molar absorptivity). Because you have trouble recognizing valid IUPAC names, here is a concise definition I think will help: IUPAC names are official, systematic chemical names following International Union of Pure and Applied Chemistry (IUPAC) rules. They typically:
  - Identify the main structure (longest carbon chain or ring).
  - Use prefixes (e.g., “methyl,” ”chloro”) for substituents, along with numerical locants (e.g., 1-, 2-, 3-) showing where those substituents attach.
  - End with a suffix naming the principal functional group (e.g., “-ol,” “-oic acid,” “-one”).
  - Indicate stereochemistry when relevant (e.g., (*R*)/(*S*), (*E*)/(*Z*), *cis*/*trans*).

Trivial or trade names (e.g., “benzene,” “acetone,” “chloroform”) may not be strictly systematic, but some are widely accepted by IUPAC. Names that lack clear IUPAC formatting or key elements-such as unnumbered substituents-are typically not valid IUPAC names. Do not perform an extraction if there is no valid IUPAC (or other common name that can be parsed into a structure) name present in the paper.

To demonstrate the value of robust prompt construction, we performed a 3 tiered ablation test on a small subset of papers from our reference photophysical dataset which we could quantify the performance of LoA on. The first run of LoA contained the full prompt exactly as shown above. The second only contained this prompt above up through point 2 in the list of guidelines, everything after was thrown out. For the final ablation step we reduced the prompt down to only the major part of the first sentence: “Extract information about small molecules with photophysical data from the provided research paper.”. This represents absolutely maximal ablation of prompt while still retaining clear instruction on the task. Because we ran this experiment on only 30 papers, we only measured relative performance between ablation levels on properties for which we extracted more than 5 reported values, which turned out to be absorption max, emission max, and quantum yield (Figure 8).

**Figure 8:**
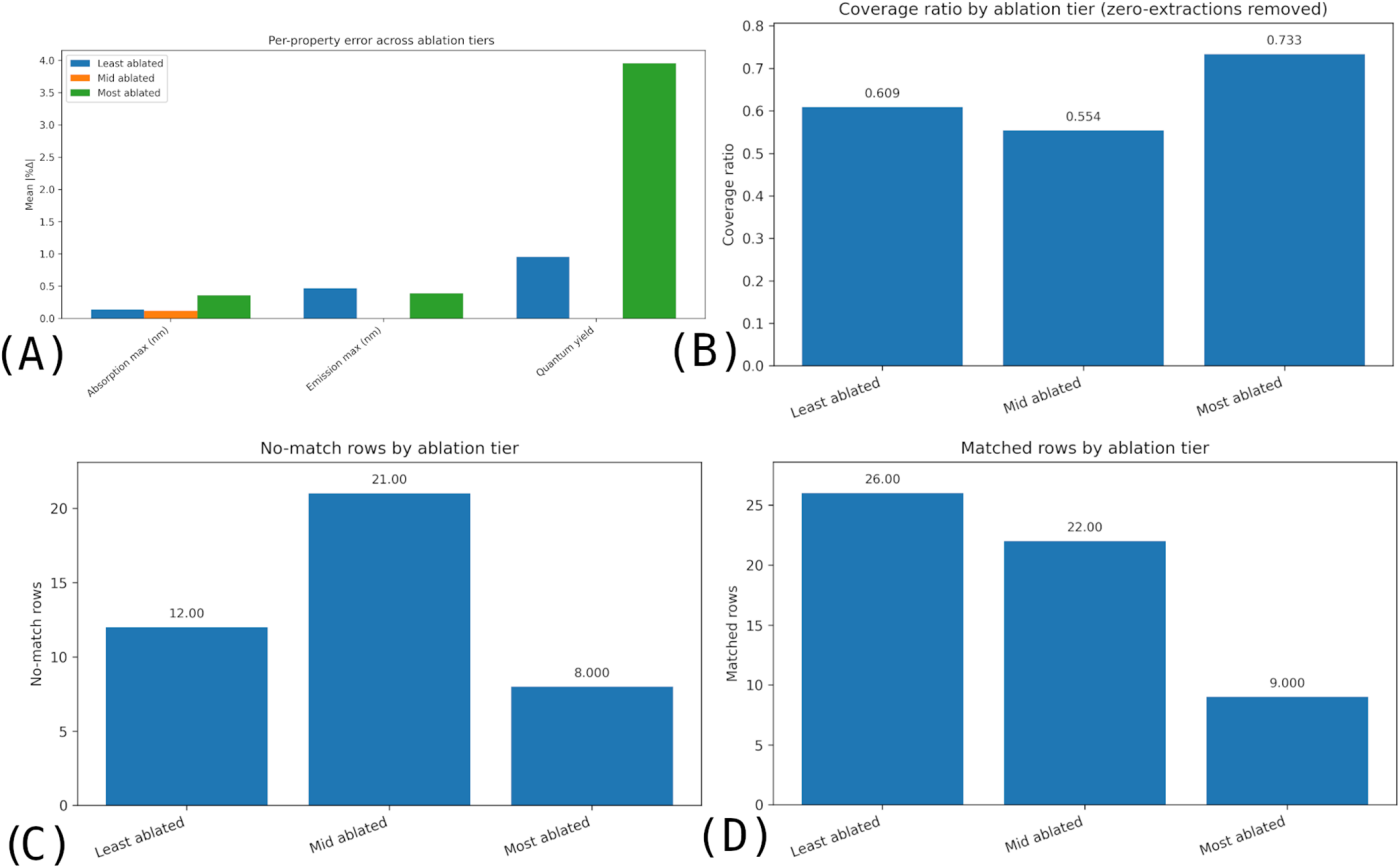
Four metrics collected over the three tiers of ablation. Ablation 1 is unablated, ablation 2 is partly ablated, and ablation 3 is maximally ablated. (A) Error rates per property and ablation level. (B) The ratio of extracted to total possible extracted data points by ablation level, excluding papers which failed. (C) The number of rows which we could not match to our dataset based on ablation level. (D) The number of rows we could match based on ablation level.

The results of this experiment were somewhat surprising, in that the ablation initially appeared to improve results; however, examining coverage statistics reveals this to be incorrect. As expected, the most ablated did suffer performance losses, most significantly in the extraction of Quantum Yield values. However, across all three properties, the mid tier ablation had the lowest percentage error, which would be surprising. However, looking at the matched rows by tier and the no-match rows by tier starts to paint a clearer picture of the benefit of a well-crafted prompt. A steady drop in the number of matched rows can be observed through ablation. As well, while the mid tier ablation did produce many rows in total, many of them did not match. It appears that the primary benefit of a well-crafted prompt is not to improve numerical precision, though it does appear to do this, but to increase coverage and to ensure extracted information is relevant. This confirms that the prompt we should be using is the one shown above, and as such we have used it to generate a large-scale dataset.

We obtained just over 11,000 results in 7 days running on a consumer-grade desktop computer equipped with a GeForce RTX 4090 and 64 GB of RAM. It is noteworthy that we did not use a multimodal model for the assembly of this dataset, due to the monetary cost of capable APIs. We manually validated selections of results (see Table 5) and determined that the correct name and absorption were obtained in approximately 80% of the entries. A small portion of results was verified before randomizing the order of results, and we observe that this did not greatly impact the final accuracy scores.

**Table 5:** Correct Name and Absorption Counts

counts:	correct name	correct absorption
TRUE	48	39
FALSE	12	8
%TRUE	80.00%	82.98%

only sorted:	correct name	correct absorption
TRUE	36	27
FALSE	8	5
%TRUE	81.82%	84.38%

Experimental measurements do not need to be perfect for many chemical machine learning applications.^28^ For biological targets, error thresholds vary by algorithm: Naive Bayes Networks remain predictive with up to 39% training error, whereas Random Forests and Probabilistic Neural Networks lose effectiveness at 29% and 20% error, respectively performance being measured via receiver operating characteristic area under the curve (ROC AUC) (>0.7) or mean top 10% IC_50_ (<750 nM).^29^ Materials research employs calibrated ensemble error bars to support predictions across 33 properties, and rigorous data collection in biodegradability studies yields balanced accuracy of as high as 94.2%.^30^ Other domains, including Quantitative Structure–Activity Relationship (QSAR) modeling and nuclear magnetic resonance (NMR) chemical shift prediction, show that models sometimes exceed the nominal quality of their training data.^31^ These findings indicate that an acceptable experimental error depends both on the chemical domain and the machine learning approach used.^28^ The data obtained using LoA can currently be directly used for machine learning applications, without requiring human examination of every entry in the resulting dataset, when the correct machine learning algorithm is used downstream. For more error sensitive machine learning methods, better open-source multimodal LLMs will need to be developed and tested to achieve higher accuracy rates.

### Comparison to Existing Tools

To get an accurate gauge of LoA’s performance, we selected two popular chemical data extraction tools to compare against: ChemDataExtractor2 and OpenChemIE. For OpenChemIE, as they provide a ground truth set of extracted chemical reactions with reference papers, we ran LoA on their existing dataset to provide a comparison. In the case of ChemDataExtractor2, ground truth final answers were not provided, nor were their extracted values. We instead provide a qualitative comparison, as the efficacy of ChemDataExtractor2 is directly related to the quality of parser designed for it.

The OpenChemIE R-group resolution reference dataset contains 1007 individual reactions collected from 78 images over 48 papers,^12^ all of which we were able to obtain for comparison. Our LoA job was set up with a minimal extraction schema, where the only information to be extracted was the reactants and products for reactions presented in the papers. We implemented an option to utilize a companion to DECIMER, DECIMER-image-segmentation,^32^ which breaks down complex reaction diagrams into individual molecular diagrams. To properly utilize this package in LoA, we run DECIMER on each individual molecular diagram produced via segmentation, and reassemble information for the LLM in the body of the paper afterward.

As an initial comparison, we manually validated 71 entries from their provided R-group resolution dataset re-extracted with LoA. Notably, the R-group resolution dataset only contains the most difficult examples in which groups that are not drawn explicitly must be inferred from the body of the text and other diagrams. Upon comparison, we noticed several issues with direct SMILES comparison, most notably in stereochemistry. Many figures do not explicitly define stereochemistry, especially with an R-group involved. As can be seen in Figure 9, LoA manages to preserve explicitly ambiguous stereochemistry in the output SMILES, whereas the reference dataset does not include explicit ambiguous stereochemistry. Similarly, there are other minor differences in SMILES extraction which require assumptions, such as nonunique traversal paths, different starting atoms, resonance representations, or chiral conventions, which may cause issues with direct SMILES comparison. That said, our manual validation shows that 70/71 (∼98.6%) of the LoA-extracted reactions were presented in the given papers explicitly. However, this is a small dataset and further study would be required to truly assess LoA’s abilities on the task of chemical reaction extraction, though these results are promising.

**Figure 9:**
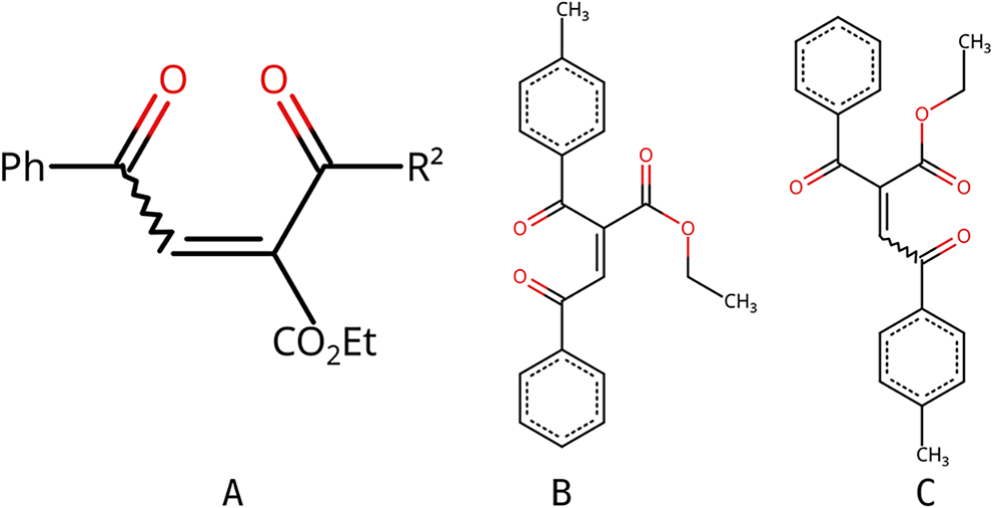
A comparison of two extracted structures to the original reference structure from one of the articles in the R-group resolution dataset provided for OpenChemIE evaluation.^12,33^ (A) The structure provided in the image of the original paper, notably containing an abbreviated phenol as well as an R group, which could be one of a few possible groups defined in the body of the text.^33^ (B) The provided corresponding reference structure for one of the possible R groups, in the R-group resolution dataset. (C) The LoA extracted version of the same structure, which preserved explicit unknown stereochemistry of the benzoyl group.

ChemDataExtractor2 requires the user to manually define a custom parser, as it is a programmatic extractor^3^ While we defined a model for extraction of photophysical properties which matches our schema layout for extraction in LoA, we found that there were a large number of differences in capabilities that made direct comparisons challenging. For example, ChemDataExtractor2 uses BERT, which has a limit of 512 tokens, and does not yet have a function to read tables that are given as images rather than parseable text. Additionally, ChemDataExtractor2 gives common names used within the papers instead of SMILES strings.^3^ When we ran ChemDataExtractor2 on the same set of papers which we ran LoA on to validate against the existing photophysical dataset, we did not obtain sufficient data via ChemDataExtractor2 for a quantitative comparison. That said, we primarily see the differences as an issue of scale and user choice: the flexibility of LoA can be an improvement for extracting complex chemical data that does not lend itself as well to a rigid programmatic framework, while ChemDataExtractor2 provides exact control over desired extraction properties.

We also quantified ChemDataExtractor2’s performance on this task in terms of speed. We had ChemDataExtractor2 run on a high performance computing node with 6 CPUs. Over the course of 49 h we were able to process 177 PDF files. A few papers took an unusually long time to process and eventually timed out. The average time to process each paper was 17 min. LoA, which at it is slowest (using a locally hosted LLM), processes papers in an average time of about 1 min per paper. This rate can be pushed even further if the user has access to an external API for LLM inference.

## Conclusions

Automatically extracting chemical data from the literature provides a way to obtain large experimental datasets, particularly for niche values that can span a variety of literature and presentation norms. We demonstrate that our Librarian of Alexandria (LoA) package provides a way of extracting such data with LLMs, allowing both natural language prompting and LLM-swapping in a modular fashion. We provide example inputs, outputs, and a Colab-friendly Jupyter Notebook on Github. We believe LoA provides a needed environment to test the capabilities and accuracies of a variety of LLMs on chemical literature, including their capabilities to properly extract numerical, structural and taxonomical features.

## Supplementary Material



## Data Availability

All source code, CSV files from which the results have been tabulated, as well as all input files used to generate them, are available on our stable github page and through Zenodo: https://github.com/arwalkerlab/LoA-Stable, 10.5281/zenodo.15328297. This includes a Jupyter notebook for running as well. A list of all relevant files with descriptions is available in the SI document.

## Notes

(i) Complete tabulated extraction results for chromophore, metal–organic framework (MOF), and p*K* datasets across all evaluated model configurations, including raw outputs and corresponding user annotations of check-model performance; (ii) validation outputs generated using the provided validation workflow, including categorized valid, invalid, and no-information results, as well as annotated validation tables; (iii) summary tables comparing check-model performance across models and tasks; (iv) finalized datasets used in the manuscript, including the spectroscopic dataset and comparison datasets; (v) scripts used for data processing, validation, sampling, and evaluation (including validate_results.py, sample_results.py, and evaluation utilities); (vi) archived logs from all computational runs; and (vii) full evaluation outputs, including histograms, scatter plots, and box plots for all extracted properties (e.g., absorption and emission maxima, fluorescence lifetimes, quantum yields, molecular weights, and full width at half-maximum values), along with text summaries of evaluation metrics and records of unmatched or invalid entries; access information for the full dataset repository and includes additional figures generated during analysis that are not shown in the main text; All files are organized by task and model configuration, with descriptions provided (PDF)
