# Supplementary material for: Librarian of Alexandria: A Modular Chemical Data Extraction Pipeline to Compare LLM Performance
Source: J Chem Inf Model. 2026 Jun 9;66(12):6921–32. doi: 10.1021/acs.jcim.6c00374 (PMC13292210; doi:10.1021/acs.jcim.6c00374)
Supplement: Supplementary file 1 [file ci6c00374_si_001.pdf]

Supporting Information

Librarian of Alexandria: A Modular Chemical Data Extraction Pipeline to Compare LLM Performance

Morgan Grougan[1], Janya Subasinghe[1], Mark A. Hix[1], and Alice R. Walker[1*]

1. Department of Chemistry, Wayne State University, Detroit MI, USA

E-mail: arwalker@wayne.edu

All generated tables can be found on the LoA-Stable github under the folder 'paper_data'; https://github.com/MorganRO8/LoA-Stable/tree/main/paper_data
Note: Unless otherwise stated, all jobs were run using the hi-res method.

+ chr: Folder containing all of the generated CSV files for jobs focusing on chromophore extraction. This does not include the final chromophore-adjacent job performed, as it was generalized to represent all spectroscopic data, we kept that separate.

    - deep8_chr_falcon10_hi.csv: File containing results from a job utilizing deepseek-r1:8b for the extraction model and falcon3:10b for the check model, as well as hi-resolution mode. This was not used to generate any data mentioned in the paper.

    - deep_chr_falcon_hi.csv: Goes alongside the next file, was intended to be used to get a comparison between the hi and low resolution methods using deepseek-r1:32b and falcon3:10b.

    - deep_chr_falcon_low: Identical parameters to the previous job aside from strictly using the low-res processing method. Both jobs were constrained to scraping sources which return PDF files to maximize the difference in processed results.

    - mistral_7b-instruct-v0.3-fp16_chromoFinal.csv: Contains raw data alongside user annotations on check model quality per the standards outlined in the paper. This job was run using mistral:7b-instruct-v0.3-fp16 as both the check and extraction model.

- nemotron_chr.csv: Contains raw data alongside user annotations on check model quality per the standards outlined in the paper. This job was run using nemotron:latest (nemotron:70b) for both the check and extraction model.

- openai_o1_mini_chr.csv: Contains raw data alongside user annotations on check model quality per the standards outlined in the paper. This job was run using o1-mini, which was offered by OpenAI via their API at the time of running. Since then, this model has been replaced, and may or may not still be available via API.

- openai_o1_preview_chr.csv: Very similar to the previous, this contains results alongside user annotations for o1-preview as the check and extraction model. Again, this model was available via API at the time of running, and may or may not be now as it has been replaced by better models.

- qwen2_7b-instruct-fp16_chromophores.csv: Contains raw data alongside user annotations on check model quality per the standards outlined in the paper. This data was generated using qwen2:7b-instruct-fp16 for both the check and extraction model.

+ mof: Folder containing all the CSVs pertaining to the jobs in which we attempt to extract information about Metal-Organic Frameworks from scraped literature, using a variety of models.

  - falcon_deepseek32_mof.csv: Contains raw data alongside user annotations on check model quality per the standards outlined in the paper. This CSV was generated using falcon3:10b for the check model, and deepseek-r1:32b for the extraction model.

  - mistral_7b-instruct-v0.3-fp16_mof_extraction.csv: Contains raw data alongside user annotations on check model quality per the standards outlined in the paper. This CSV was generated using mistral:7b-instruct-v0.3-fp16 as both the check and extraction model.

  - nemo_mof.csv: Contains raw data alongside user annotations on check model quality per the standards outlined in the paper. This CSV was generated using nemotron:latest (nemotron:70b) for both the check and extraction model.

  - o1_mini_mof_26.csv: Contains raw data alongside user annotations on check model quality per the standards outlined in the paper. This CSV was generated using o1-mini for both the check and extraction model.

  - o1_preview_mof_304.csv: Contains raw data alongside user annotations on check model quality per the standards outlined in the paper. This CSV was

generated using o1-preview for both the check and extraction model.

- qwen2_7b-instruct-fp16_mof_extraction.csv: Contains raw data alongside user annotations on check model quality per the standards outlined in the paper. This CSV was generated using qwen2:7b-instruct-fp16 for both the check and extraction model.

+ pKa: Folder containing all the CSV files pertaining to jobs done that attempt to extract pKa information from literature.

    - falcon_deep_pka.csv: Contains raw data alongside user annotations on check model quality per the standards outlined in the paper. This CSV was generated using falcon3:10b for the check model, and deepseek-r1:32b for the extraction model.

    - mistral_7b-instruct-v0.3-fp16_pka-extraction-schema.csv: Contains raw data alongside user annotations on check model quality per the standards outlined in the paper. This CSV was generated using mistral:7b-instruct-v0.3-fp16 as both the check and extraction model.

    - nemotron_pKa.csv: Contains raw data alongside user annotations on check model quality per the standards outlined in the paper. This CSV was generated using nemotron:latest (nemotron:70b) for both the check and extraction model.

    - pKa_o1_mini.csv: Contains raw data alongside user annotations on check model quality per the standards outlined in the paper. This CSV was generated using o1-mini for both the check and extraction model.

    - pKa_o1_preview.csv: Contains raw data alongside user annotations on check model quality per the standards outlined in the paper. This CSV was generated using o1-preview for both the check and extraction model.

    - qwen2_7b-instruct-fp16_pka-extraction-schema.csv: Contains raw data alongside user annotations on check model quality per the standards outlined in the paper. This CSV was generated using qwen2:7b-instruct-fp16 for both the check and extraction model.

+ validated_results_1: Folder containing the results of running ‘validate_results.py’ on the ‘spec.csv’ results, as well as a user annotated version to check the quality of the results.

    - invalid_results.csv: The validation script dumps all results which fail to validate here. They may fail to validate for a number of reasons. Note that the column ‘canonical_smiles’ simply refers to the identifier given, as the smiles could not be

resolved in the case of an error.

  - no_info_results.csv: The validation script dumps all results which no relevant information was obtained. So, the model itself could have failed, identified correctly that there was no info, or only provided a chemical name with no valid data.

  - valid_results.csv: All results from the original output CSV for which we could find a canonical SMILES from the given name, which also have relevant numerical data.

  - valid_results.ods: All the results from 'valid_results.csv' alongside our annotations on the correctness of specific columns.

- check_comparison.csv: Tabulated check model performance values across different tasks and models.

- check_comparison.xls: The same as 'check_comparison.csv', but in xls format to ensure that the calculations remain present upon a save and reload.

- logs.zip: The raw logs from all runs over the months in which we were collecting data.

- sample_results.py: A script that can be used to sample each of the three result types from a csv for further inspection; null, failed, and positive. The same can be achieved using custom sorting logic in CSV files, so we did it that way. However, if the user would like to we included this as a utility.

- spec.csv: The final iteration of our series of improvements on the prompt for extracting information about chromophores, now generalized to look for small molecule spectroscopic data in all its forms. This data was generated using deepseek-r1:32b for the extraction model, and falcon3:10b for the check model.

- spec_8.csv: The same as 'spec.csv' except using deepseek-r1:8b instead for the extraction model. This was not used, but served as a point of comparison.

- validate_results.py: The python script we designed to validate results from 'spec.csv'. It uses several databases to determine if the result can be parsed to a chemical structure, and whether or not it contains any relevant information. This resulted in the 'validated_results_1' folder.

+ eval: Folder containing all our tests using LoA in multimodal, and comparing against the dataset published here: https://figshare.com/articles/dataset/DB_for_chromophore/12045567/2?file=23637518

    - chromophore_quality_openai.csv: The output dataset from our short run using the OpenAI o3 model on a random subset of papers from the comparison dataset.

    - eval.py: The script we used to compare the datasets, match results, calculate accuracy, and output figures.

    - utils.py: An exact copy of utils.py from the main source code. It is placed here for eval.py to be able to function properly, as we wanted to utilize our exact verification scheme for comparing results.

    + evaluation_results: A folder containing all of the direct output from eval.py. This includes histograms and scatter plots for each property, a box blot for all properties, and text files summarizing results.

        - Absorption_max_nm_hist.png: Histogram showing absorption max extraction performance.

        - Absorption_max_nm_scatter.png: Scatter plot showing absorption max extraction performance.

        - Emission_max_nm_hist.png: Histogram showing emission max extraction performance.

        - Emission_max_nm_scatter.png: Scatter plot showing emission max extraction performance.

        - Lifetime_ns_hist.png: Histogram showing fluorescence lifetime extraction performance.

        - Lifetime_ns_scatter.png: Scatter plot showing fluorescence lifetime extraction performance.

        - Molecular_weight_g_mol-1_hist.png: Histogram showing molecular weight extraction performance.

        - Molecular_weight_g_mol-1_scatter.png: Scatter plot showing molecular weight extraction performance.

- Quantum_yield_hist.png: Histogram showing quantum yield extraction performance.

- Quantum_yield_scatter.png: Scatter plot showing quantum yield extraction performance.

- abs_FWHM_cm-1_hist.png: Histogram showing absorption FWHM extraction performance.

- abs_FWHM_cm-1_scatter.png: Scatter plot showing absorption FWHM extraction performance.

- abs_FWHM_nm_hist.png: Histogram showing absorption FWHM extraction performance.

- abs_FWHM_nm_scatter.png: Scatter plot showing absorption FWHM extraction performance.

- emi_FWHM_nm_hist.png: Histogram showing emission FWHM extraction performance.

- emi_FWHM_nm_scatter.png: Scatter plot showing emission FWHM extraction performance.

- log_e_mol-1_dm3_cm-1_hist.png: Histogram showing log molar extinction coefficient extraction performance.

- log_e_mol-1_dm3_cm-1_scatter.png: Scatter plot showing log molar extinction coefficient extraction performance.

- error_boxplot.png: A boxplot showing the errors of all extracted properties compared to one another.

- no_match_entries.txt: A text document which contains outputs for all structures that could not be automatically matched to one in the original dataset.

- multi_match_entries.txt: A text document which contains outputs for all structures that could be matched to multiple entries in the original dataset.

- invalid_smiles.txt: A text document which contains a list of all SMILES which could not be automatically parsed by rdkit into a valid structure, but may pertain to a legitimate pubchem entry.

- final_results.txt: a text based version of Table 2 in our paper. This is what eval.py outputs when run on the data.

+ CDE_comp: Folder containing the data, evaluation scripts, and generated comparison outputs for comparing LoA chromophore extractions against the CDE reference dataset. The generated figures are contained in the CDE and CDE_fair subfolders.
    + cde_100_results.csv: CSV containing the LoA extraction results used for the CDE comparison jobs.
    + eval_cde.py: The script used to evaluate LoA outputs against the CDE reference data and generate comparison figures and summary files.
    + eval_cde_fair.py: A fair-comparison version of the CDE evaluation script, used to generate the CDE_fair outputs.
    + CDE: Folder containing the direct CDE comparison outputs from eval_cde.py, including match-status text files, summary results, cache/support files, and generated figures.
        - Absorption_max_nm_hist.png: Histogram showing absorption max extraction error for the CDE comparison.
        - Absorption_max_nm_scatter.png: Scatter plot showing absorption max extraction performance for the CDE comparison.
        - Emission_max_nm_hist.png: Histogram showing emission max extraction error for the CDE comparison.
        - Emission_max_nm_scatter.png: Scatter plot showing emission max extraction performance for the CDE comparison.
        - Lifetime_ns_hist.png: Histogram showing fluorescence lifetime extraction error for the CDE comparison.
        - Lifetime_ns_scatter.png: Scatter plot showing fluorescence lifetime extraction performance for the CDE comparison.
        - Molecular_weight_g_mol-1_scatter.png: Scatter plot showing molecular weight extraction performance for the CDE comparison.
        - Quantum_yield_hist.png: Histogram showing quantum yield extraction error for the CDE comparison.
        - Quantum_yield_scatter.png: Scatter plot showing quantum yield extraction performance for the CDE comparison.
        - abs_FWHM_cm-1_scatter.png: Scatter plot showing absorption FWHM extraction performance in cm-1 for the CDE comparison.
        - abs_FWHM_nm_scatter.png: Scatter plot showing absorption FWHM extraction performance in nm for the CDE comparison.
        - emi_FWHM_cm-1_scatter.png: Scatter plot showing emission FWHM extraction performance in cm-1 for the CDE comparison.
        - emi_FWHM_nm_scatter.png: Scatter plot showing emission FWHM extraction performance in nm for the CDE comparison.
        - error_boxplot.png: A boxplot showing the errors of all extracted properties compared to one another for the CDE comparison.

- log_e_mol-1_dm3_cm-1_hist.png: Histogram showing log molar extinction coefficient extraction error for the CDE comparison.
- log_e_mol-1_dm3_cm-1_scatter.png: Scatter plot showing log molar extinction coefficient extraction performance for the CDE comparison.
- publication_figure.png: Combined publication-style summary figure for the CDE comparison.
- final_results.txt: A text based summary of the CDE comparison results output by eval_cde.py.
- no_match_entries.txt: A text document which contains outputs for structures that could not be automatically matched to one in the CDE dataset.
- multi_match_entries.txt: A text document which contains outputs for structures that could be matched to multiple entries in the CDE dataset.
- invalid_smiles.txt: A text document which contains a list of SMILES which could not be automatically parsed into valid structures for the CDE comparison.
- coverage.csv: CSV summarizing property-level coverage for the CDE comparison.

+ CDE_fair: Folder containing the fair CDE comparison outputs from eval_cde_fair.py, including filtered comparison results, worst-case summaries, match-status text files, cache/support files, and generated figures.
    - Absorption_max_nm_hist.png: Histogram showing absorption max extraction error for the fair CDE comparison.
    - Absorption_max_nm_scatter.png: Scatter plot showing absorption max extraction performance for the fair CDE comparison.
    - Emission_max_nm_hist.png: Histogram showing emission max extraction error for the fair CDE comparison.
    - Emission_max_nm_scatter.png: Scatter plot showing emission max extraction performance for the fair CDE comparison.
    - Lifetime_ns_scatter.png: Scatter plot showing fluorescence lifetime extraction performance for the fair CDE comparison.
    - Molecular_weight_g_mol-1_scatter.png: Scatter plot showing molecular weight extraction performance for the fair CDE comparison.
    - Quantum_yield_hist.png: Histogram showing quantum yield extraction error for the fair CDE comparison.
    - Quantum_yield_scatter.png: Scatter plot showing quantum yield extraction performance for the fair CDE comparison.
    - abs_FWHM_nm_scatter.png: Scatter plot showing absorption FWHM extraction performance in nm for the fair CDE comparison.
    - emi_FWHM_nm_scatter.png: Scatter plot showing emission FWHM extraction performance in nm for the fair CDE comparison.
    - error_boxplot.png: A boxplot showing the errors of all extracted properties compared to one another for the fair CDE comparison.

        - log_e_mol-1_dm3_cm-1_scatter.png: Scatter plot showing log molar extinction coefficient extraction performance for the fair CDE comparison.
        - publication_figure.png: Combined publication-style summary figure for the fair CDE comparison.
        - final_results.txt: A text based summary of the fair CDE comparison results output by eval_cde_fair.py.
        - worst_cases_summary.txt: A text summary of the largest fair-comparison errors.
        - Absorption_max_nm_worst_cases.csv: CSV containing the worst absorption max cases from the fair CDE comparison.
        - Emission_max_nm_worst_cases.csv: CSV containing the worst emission max cases from the fair CDE comparison.
        - Quantum_yield_worst_cases.csv: CSV containing the worst quantum yield cases from the fair CDE comparison.
        - coverage.csv: CSV summarizing property-level coverage for the fair CDE comparison.

+ OpenChemIE_comp: Folder containing files used for comparison against OpenChemIE-style annotated extraction outputs. This folder contains CSV data rather than generated figures.
    + Annotated_LoA_Extractions.csv: CSV containing annotated LoA extractions used for the OpenChemIE comparison.

+ polymer_eval: Folder containing the polymer-matching evaluation script, input data, and match result outputs. This folder contains python scripts, CSV files, and JSON/cache outputs rather than generated figures.
    + match_polymers.py: The script used to compare and match polymer extraction results against the polymer reference data.
    + polymer.csv: CSV containing the polymer data used as input for the matching evaluation.
    + match_results: Folder containing the direct outputs from match_polymers.py.
        - matches_only.csv: CSV containing only the rows that were successfully matched during polymer evaluation.
        - matched_merged.csv: CSV containing the merged matched outputs and reference data from the polymer evaluation.
        - summary.json: JSON summary of the polymer matching evaluation results.
        - column_stats.json: JSON file containing column-level statistics for the polymer matching evaluation.
        - headline_metrics.json: JSON file containing headline metrics from the polymer matching evaluation.
        - benchmark_config.json: JSON file containing the benchmark configuration used for the polymer matching evaluation.

- figures_manifest.json: JSON manifest describing figure-related output expectations for the polymer evaluation; no generated figure files are included in this folder.

Here are all the figures which were created but not included in the paper:
Original chromophore result figures, as mentioned above:

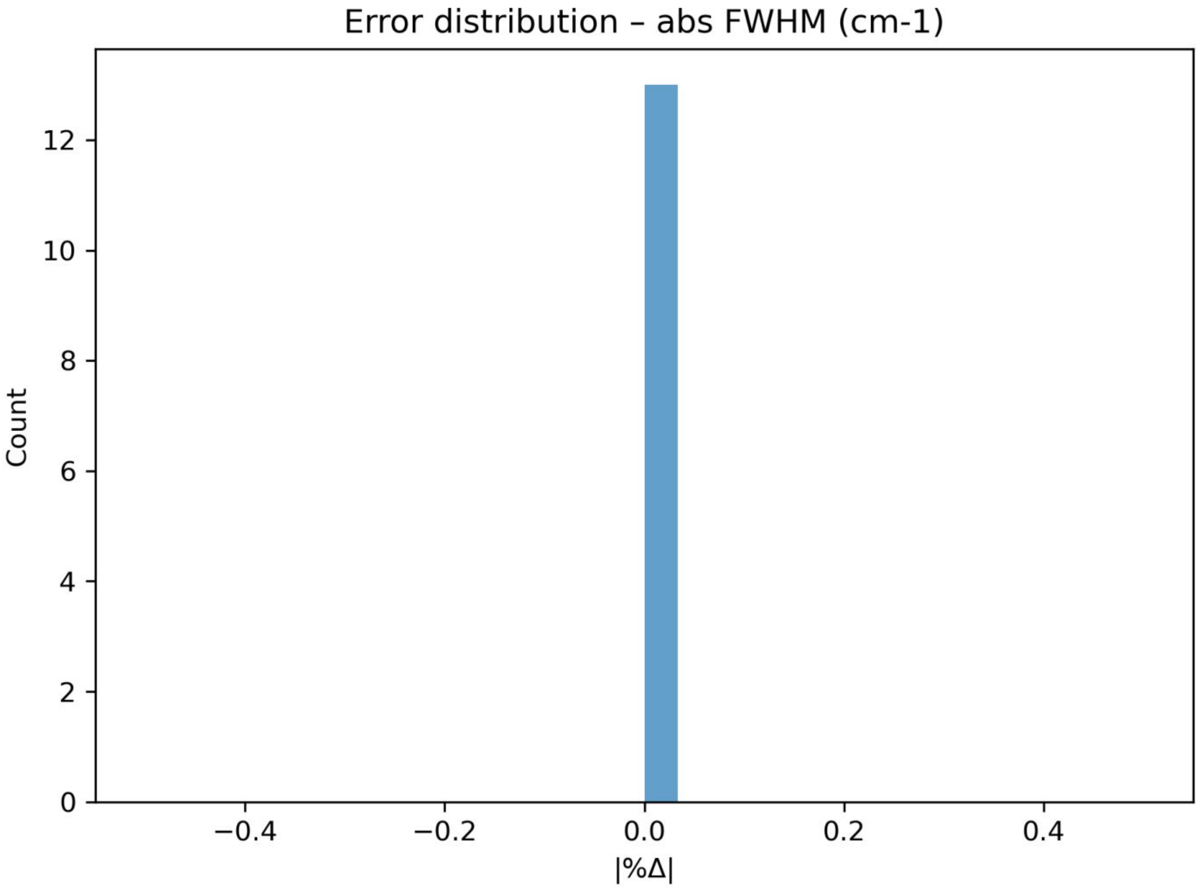

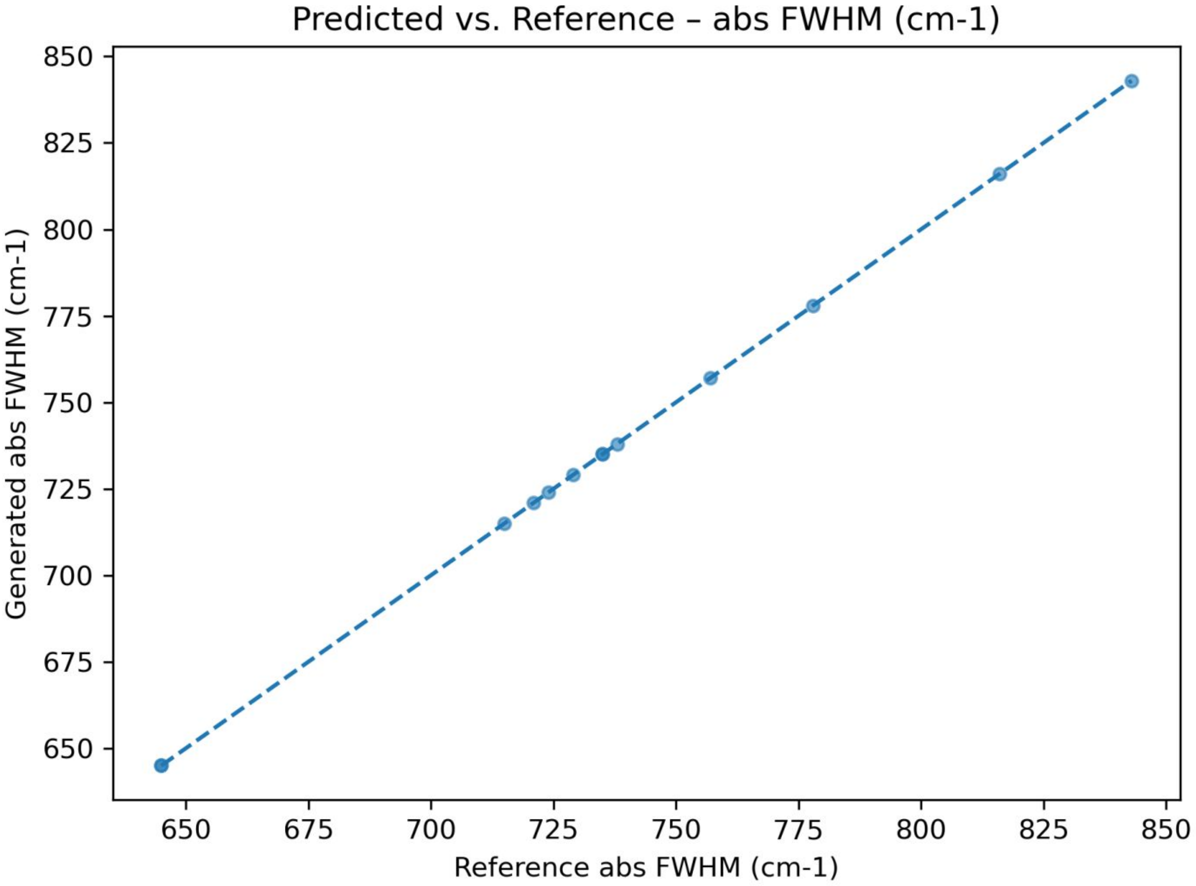
Predicted vs. Reference – abs FWHM (cm-1)
Generated abs FWHM (cm-1)
Reference abs FWHM (cm-1)
650
675
700
725
750
775
800
825
850

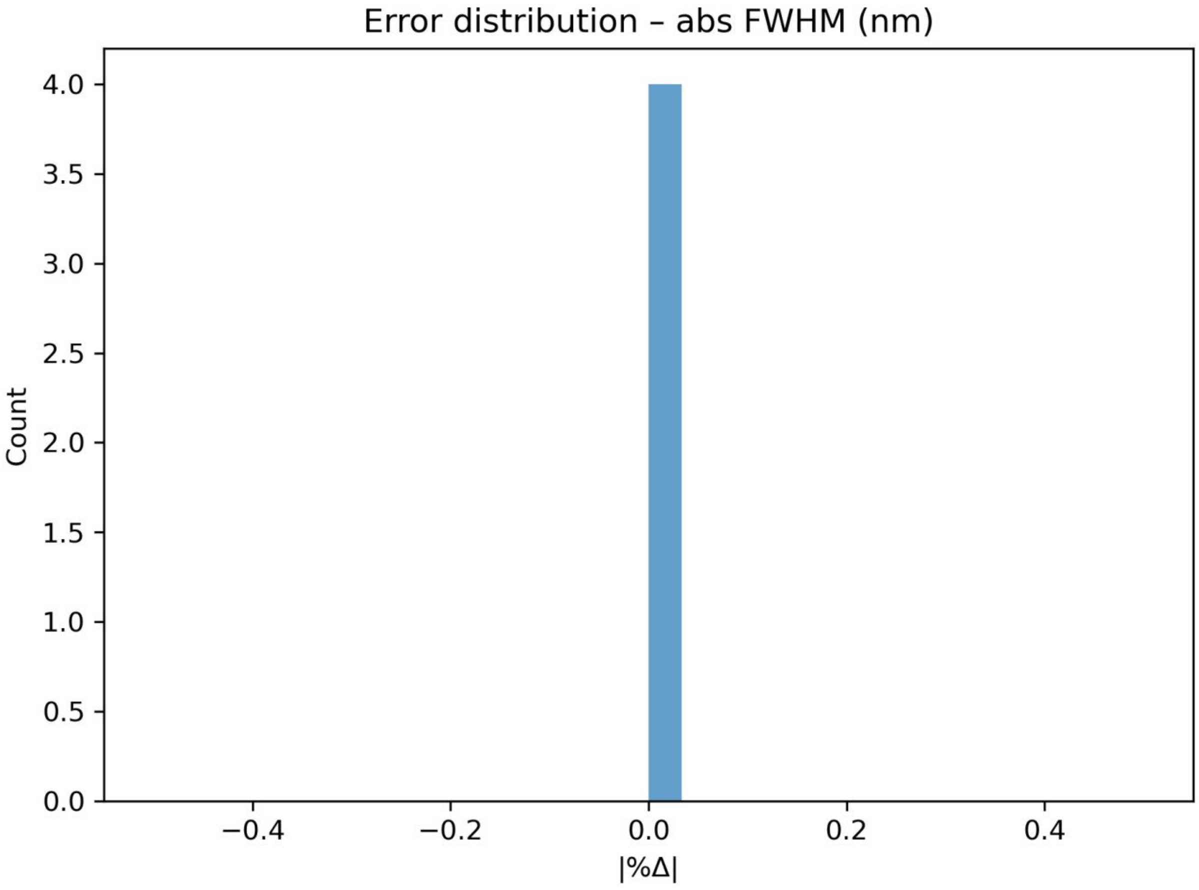
Error distribution – abs FWHM (nm)
Count
|%Δ|
4.0
3.5
3.0
2.5
2.0
1.5
1.0
0.5
0.0
−0.4
−0.2
0.0
0.2
0.4

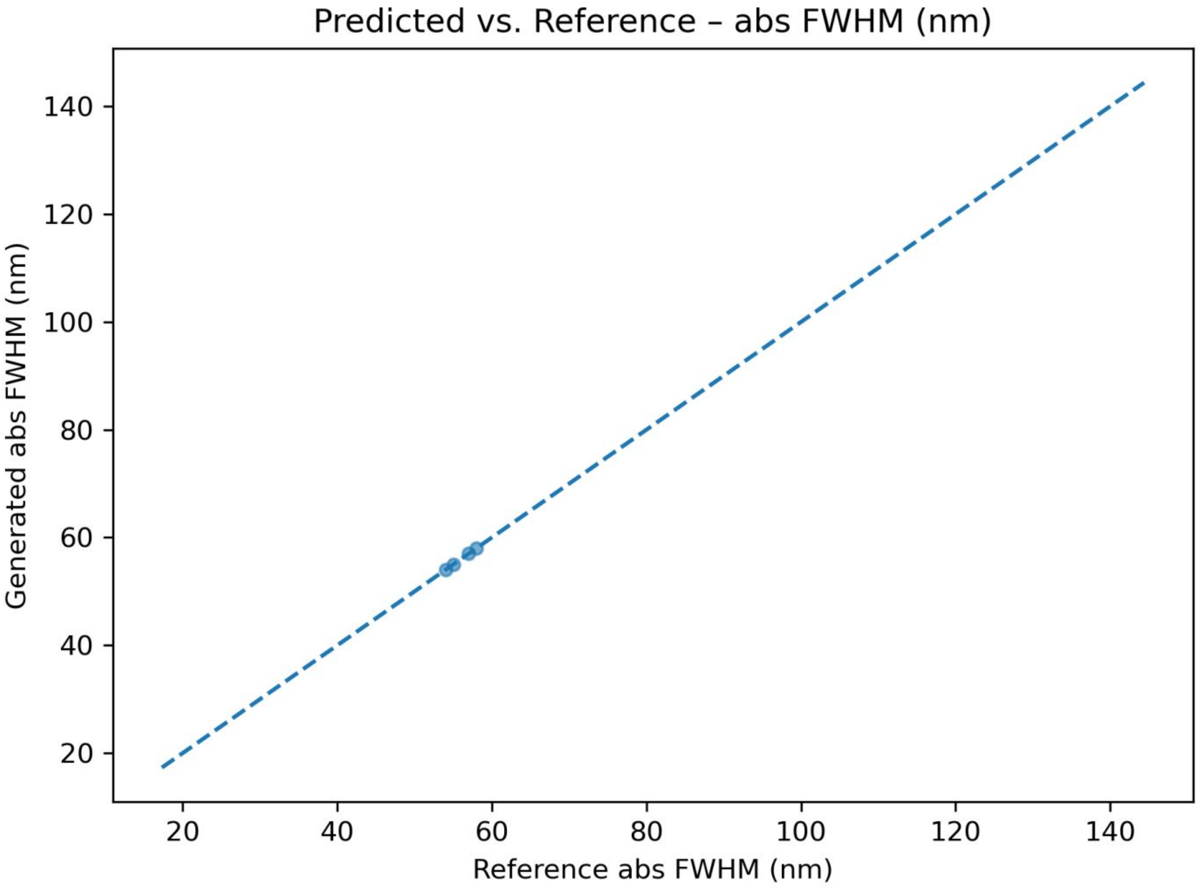
Predicted vs. Reference – abs FWHM (nm)
Generated abs FWHM (nm)
Reference abs FWHM (nm)
20
40
60
80
100
120
140

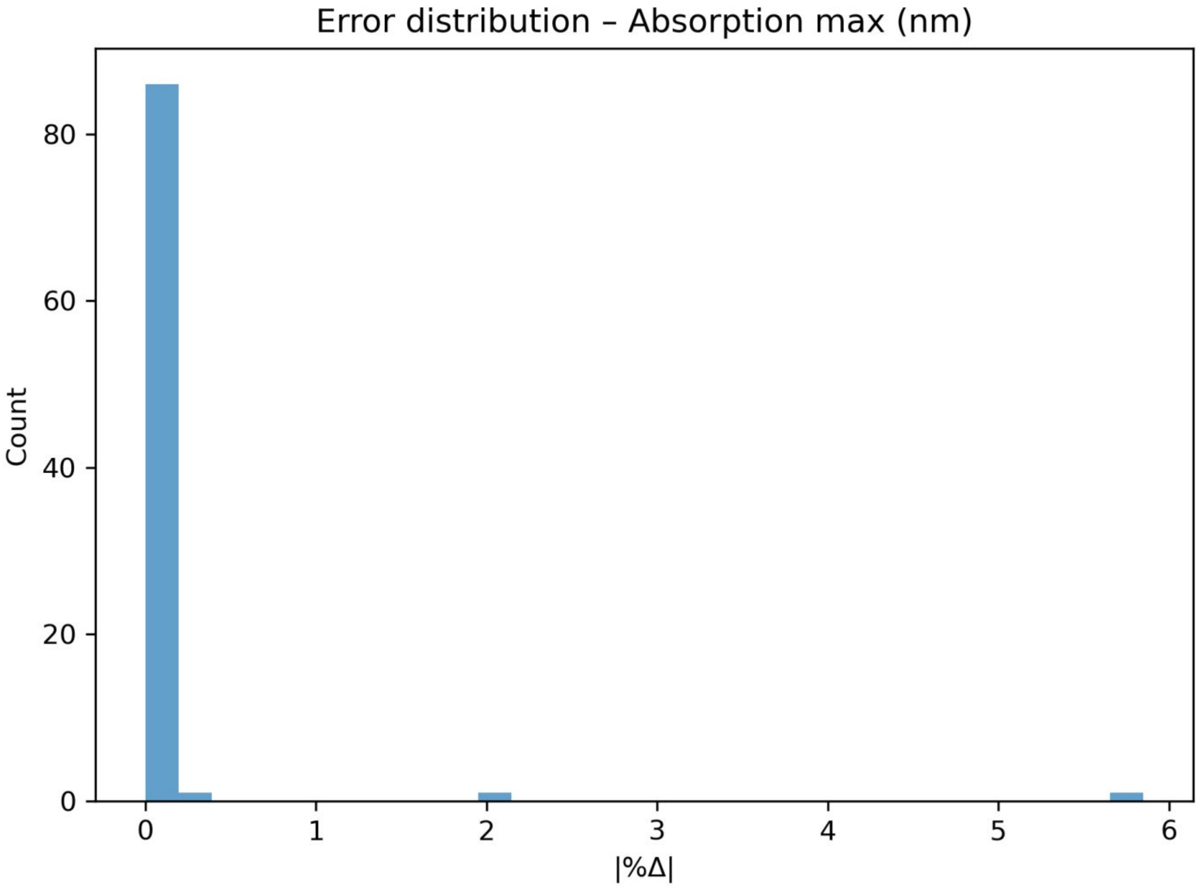
Error distribution – Absorption max (nm)
Count
|%Δ|
0
20
40
60
80
0
1
2
3
4
5
6

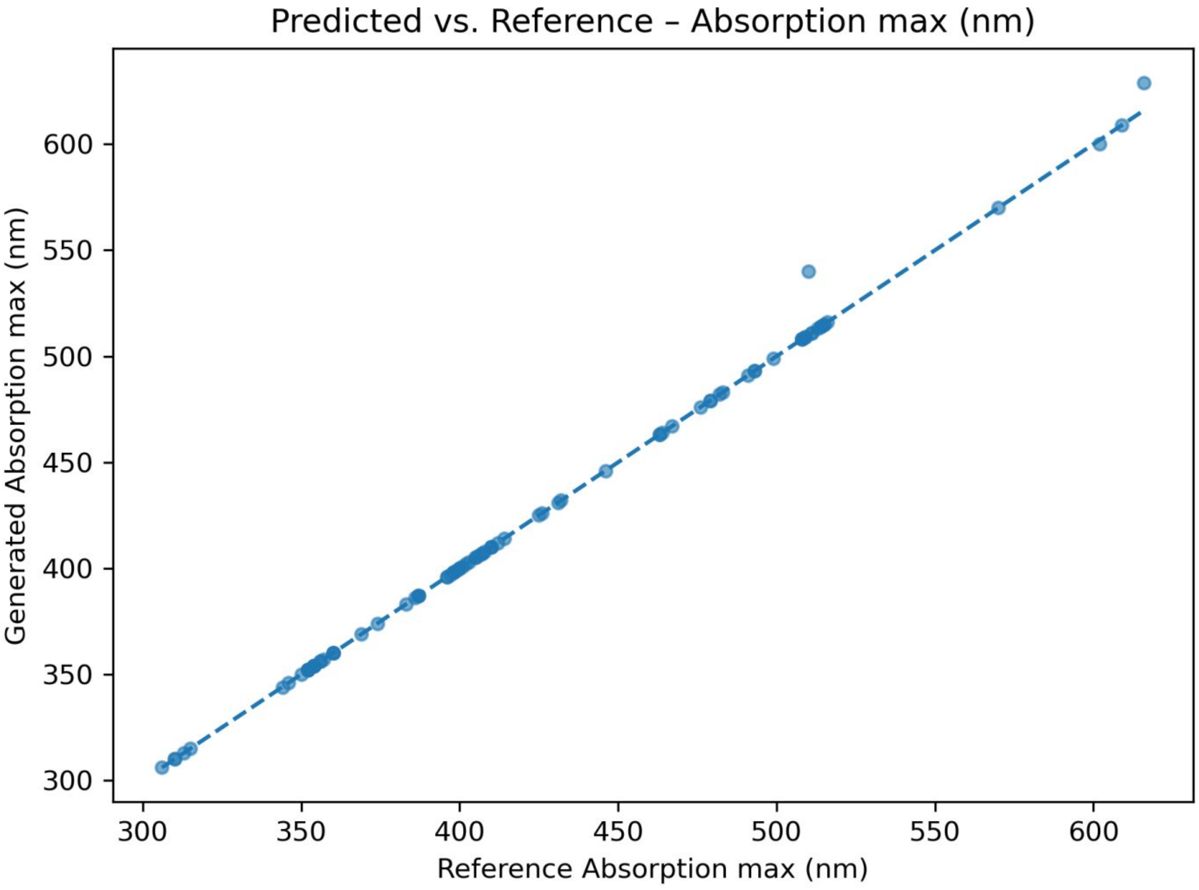

Predicted vs. Reference – Absorption max (nm)
Generated Absorption max (nm)
Reference Absorption max (nm)
300
350
400
450
500
550
600

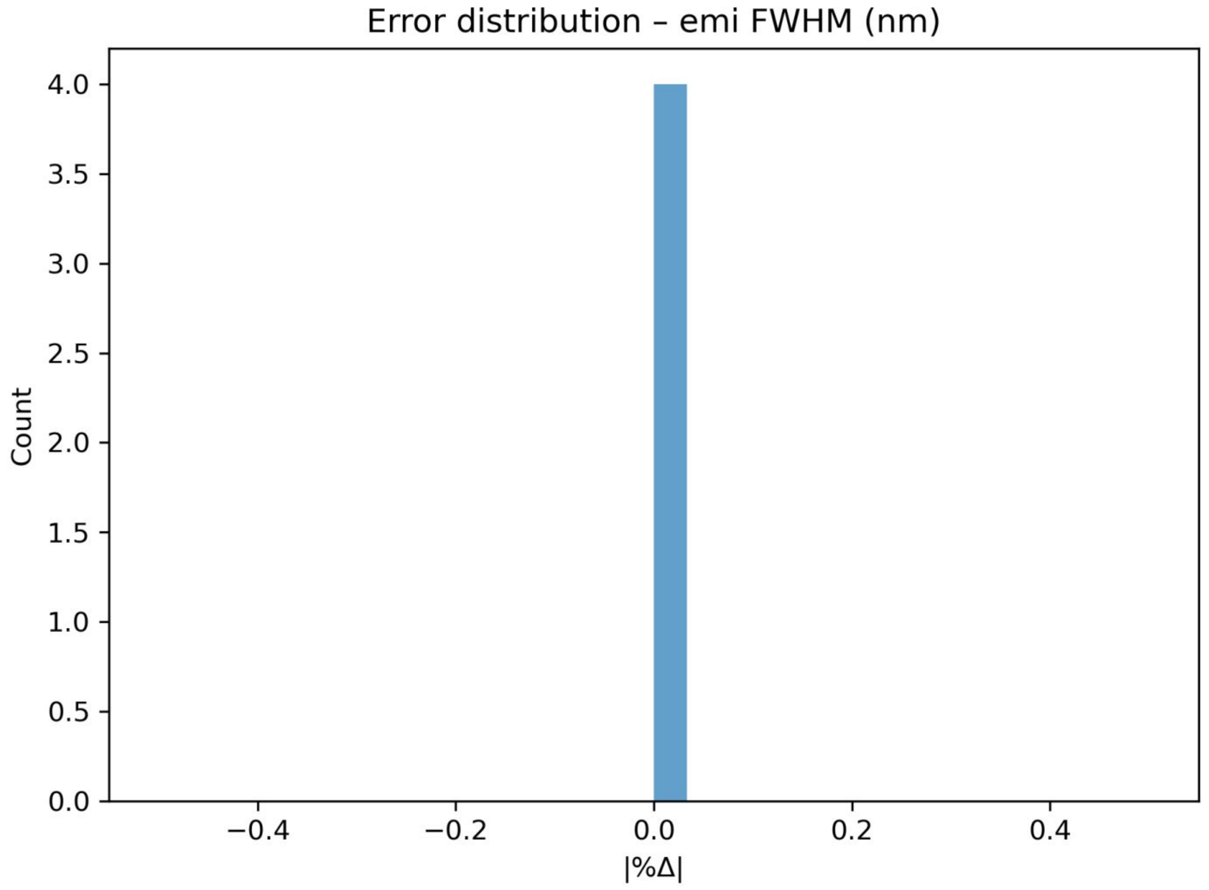
Error distribution – emi FWHM (nm)
Count
|%Δ|
4.0
3.5
3.0
2.5
2.0
1.5
1.0
0.5
0.0
−0.4
−0.2
0.0
0.2
0.4

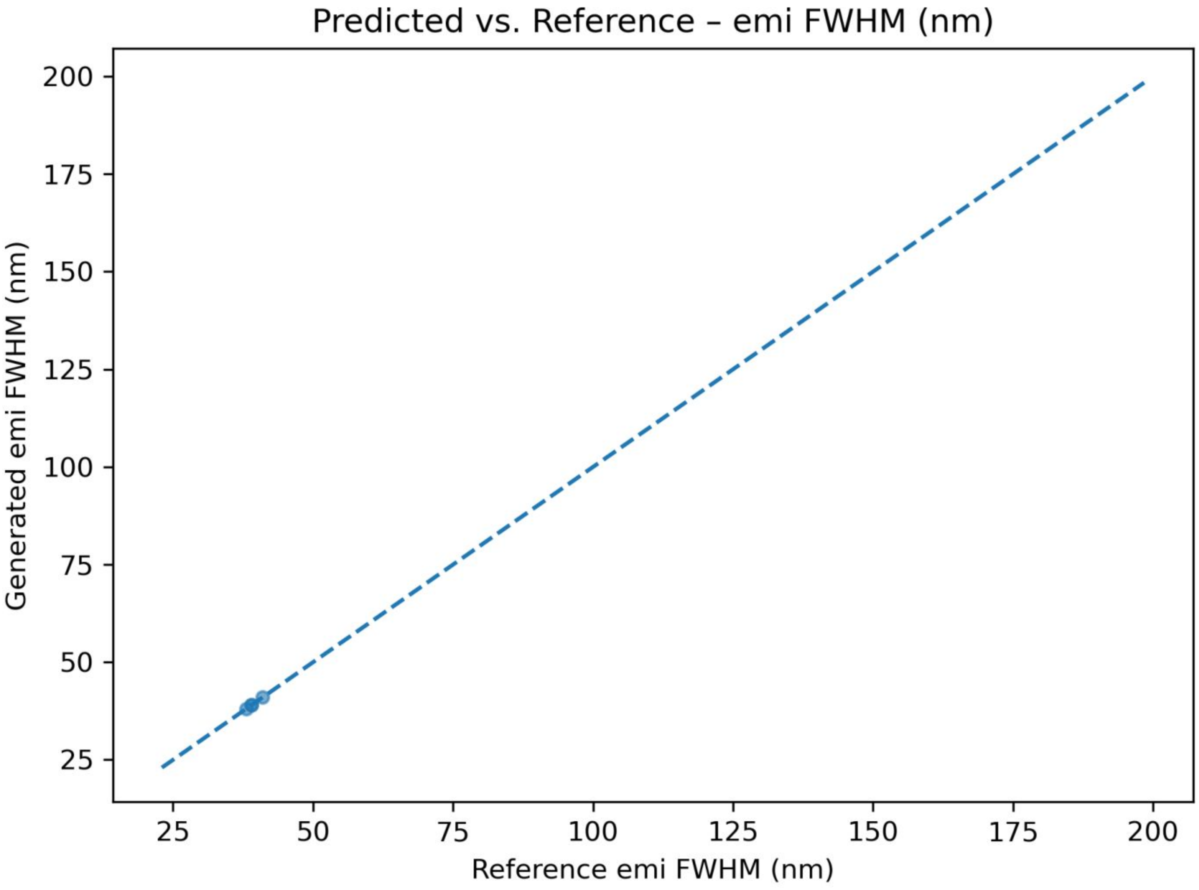
Predicted vs. Reference – emi FWHM (nm)
Generated emi FWHM (nm)
Reference emi FWHM (nm)
200
175
150
125
100
75
50
25
25
50
75
100
125
150
175
200

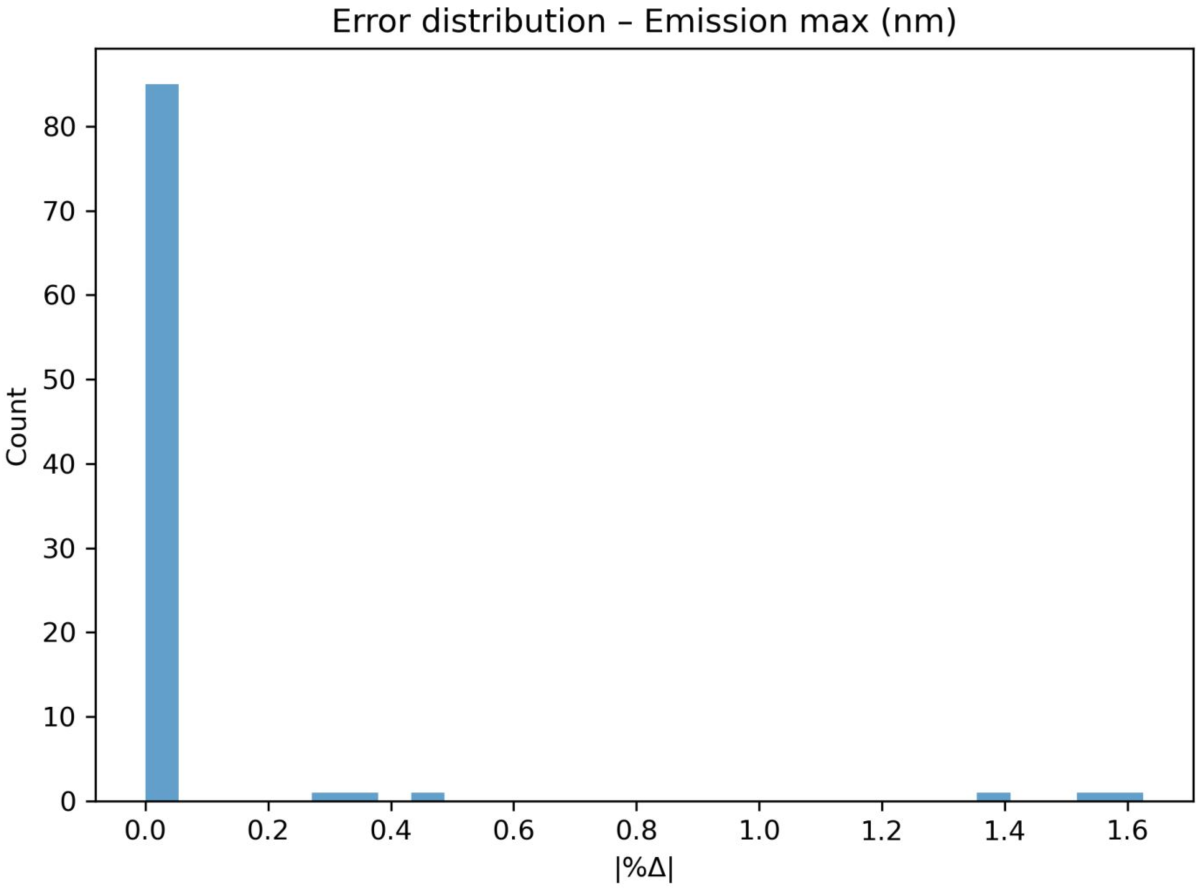
Error distribution – Emission max (nm)
Count
|%Δ|
0
10
20
30
40
50
60
70
80
0.0
0.2
0.4
0.6
0.8
1.0
1.2
1.4
1.6

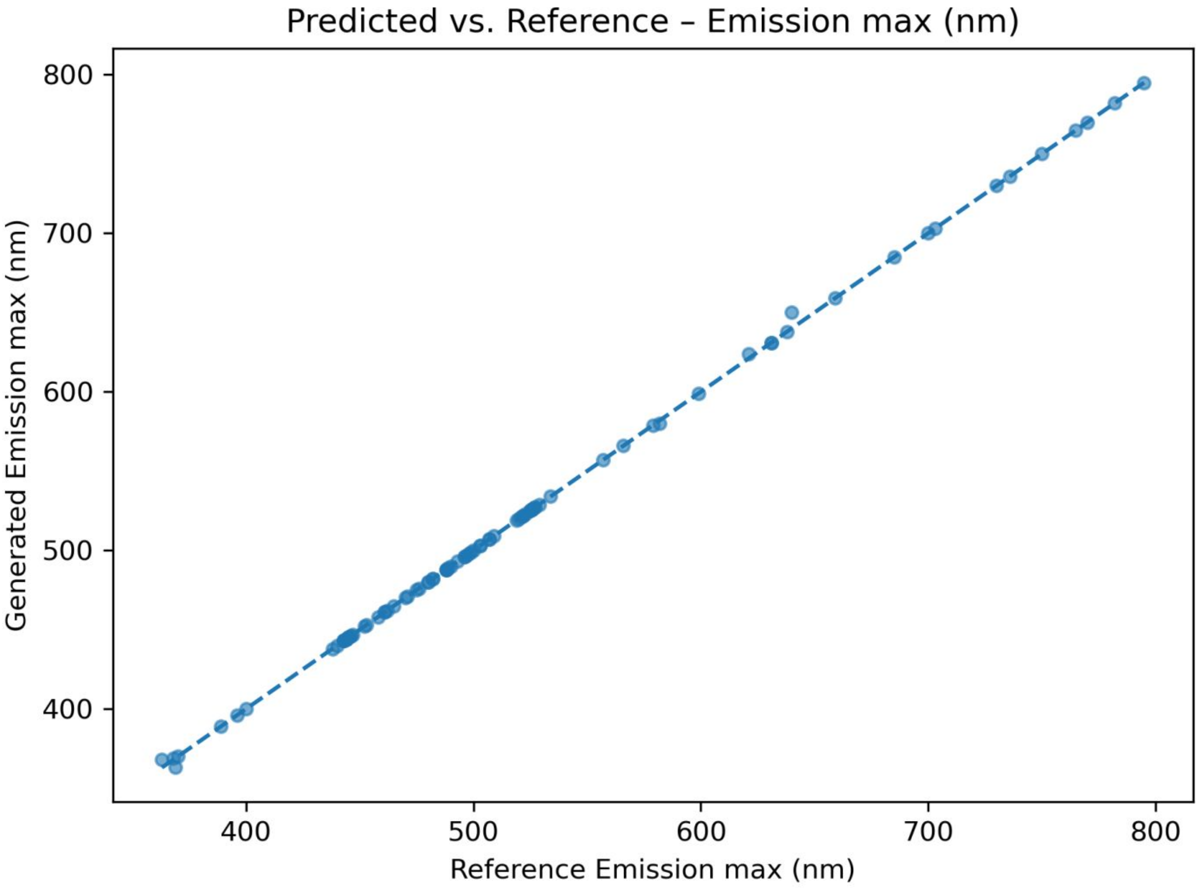
Predicted vs. Reference – Emission max (nm)
Generated Emission max (nm)
Reference Emission max (nm)
400
500
600
700
800
400
500
600
700
800

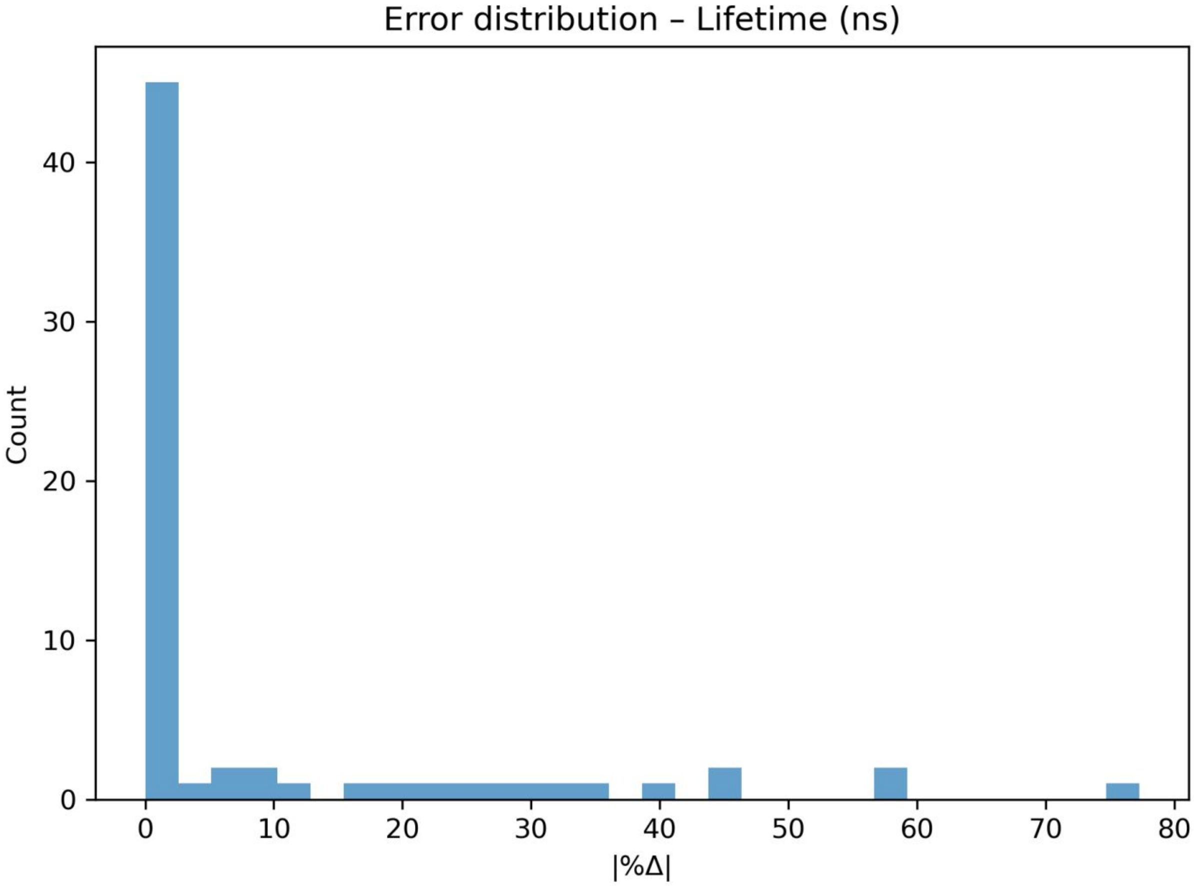

Error distribution – Lifetime (ns)
Count
0
10
20
30
40
0
10
20
30
40
50
60
70
80
|%Δ|

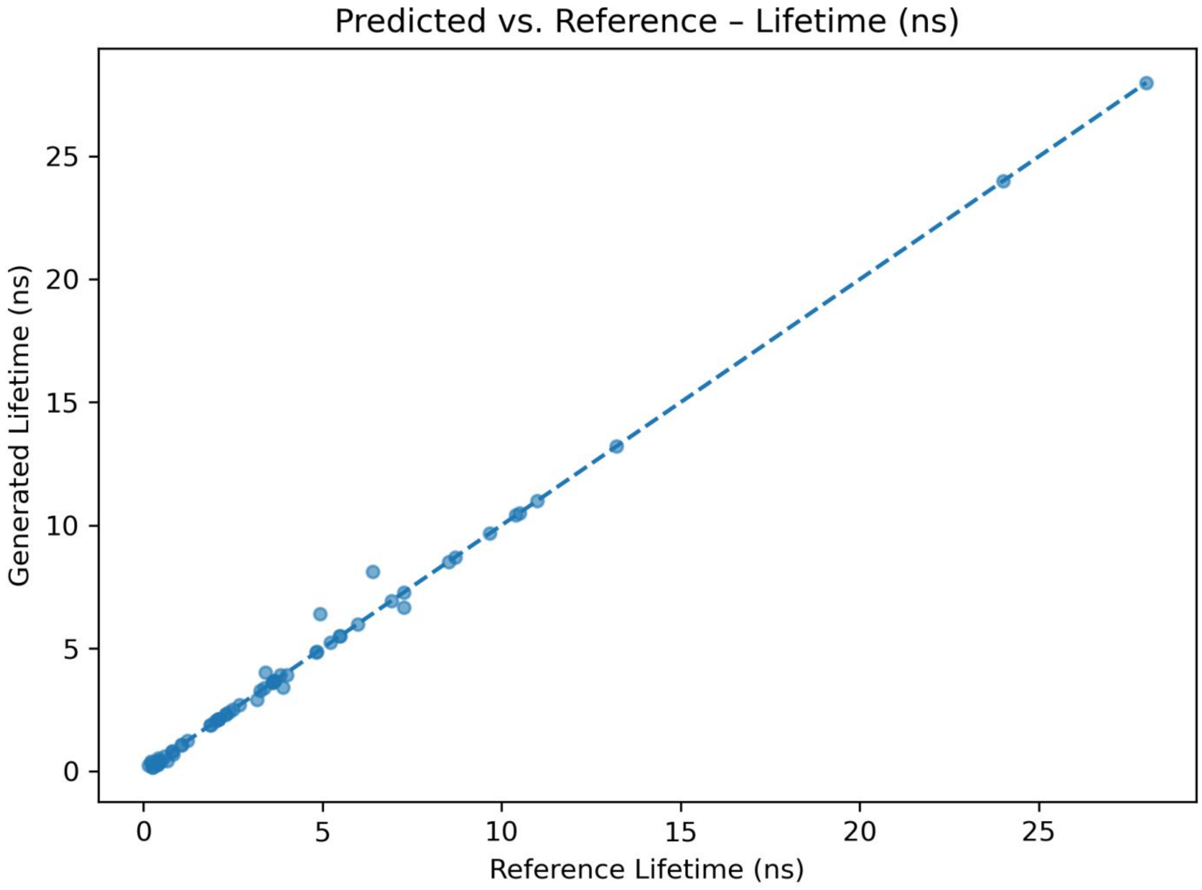

Predicted vs. Reference – Lifetime (ns)
Generated Lifetime (ns)
Reference Lifetime (ns)
0
5
10
15
20
25

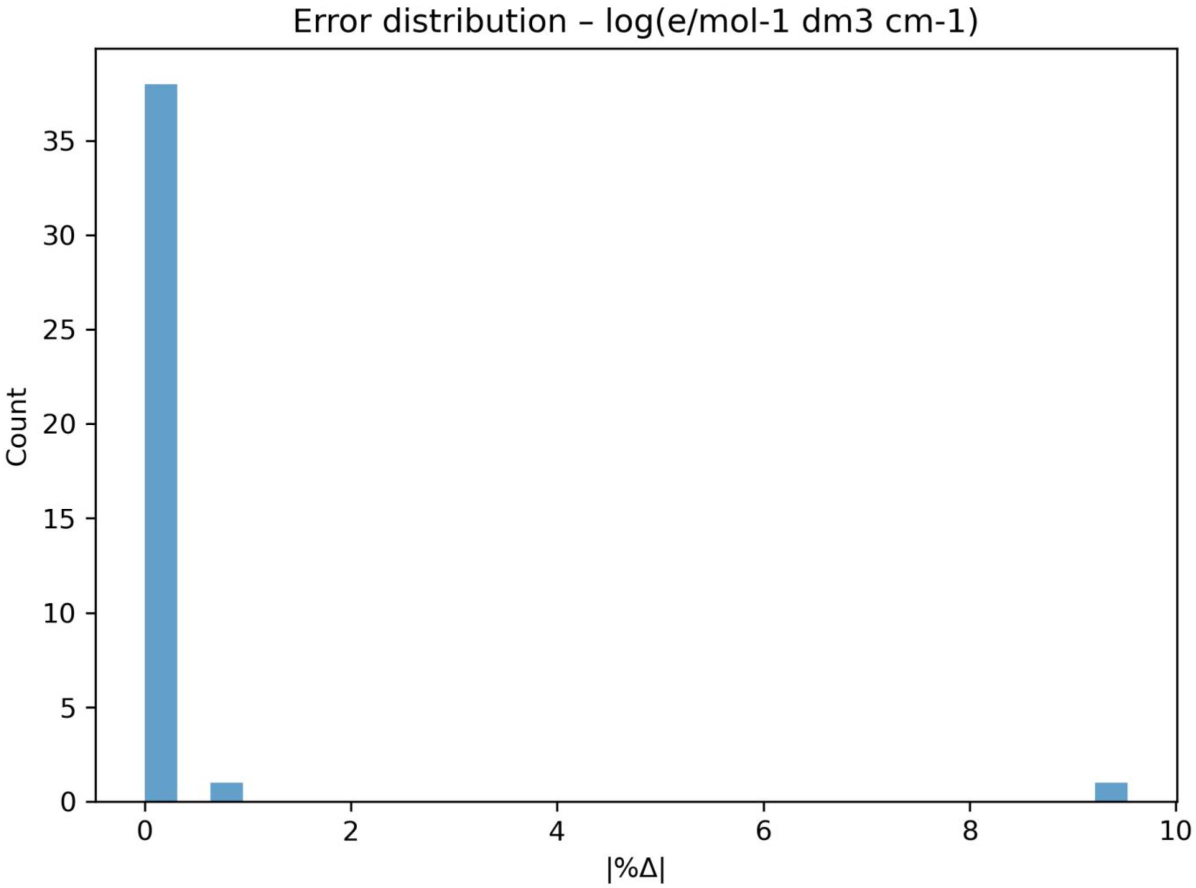
Error distribution – log(e/mol-1 dm3 cm-1)
Count
35
30
25
20
15
10
5
0
0
2
4
6
8
10
|%Δ|

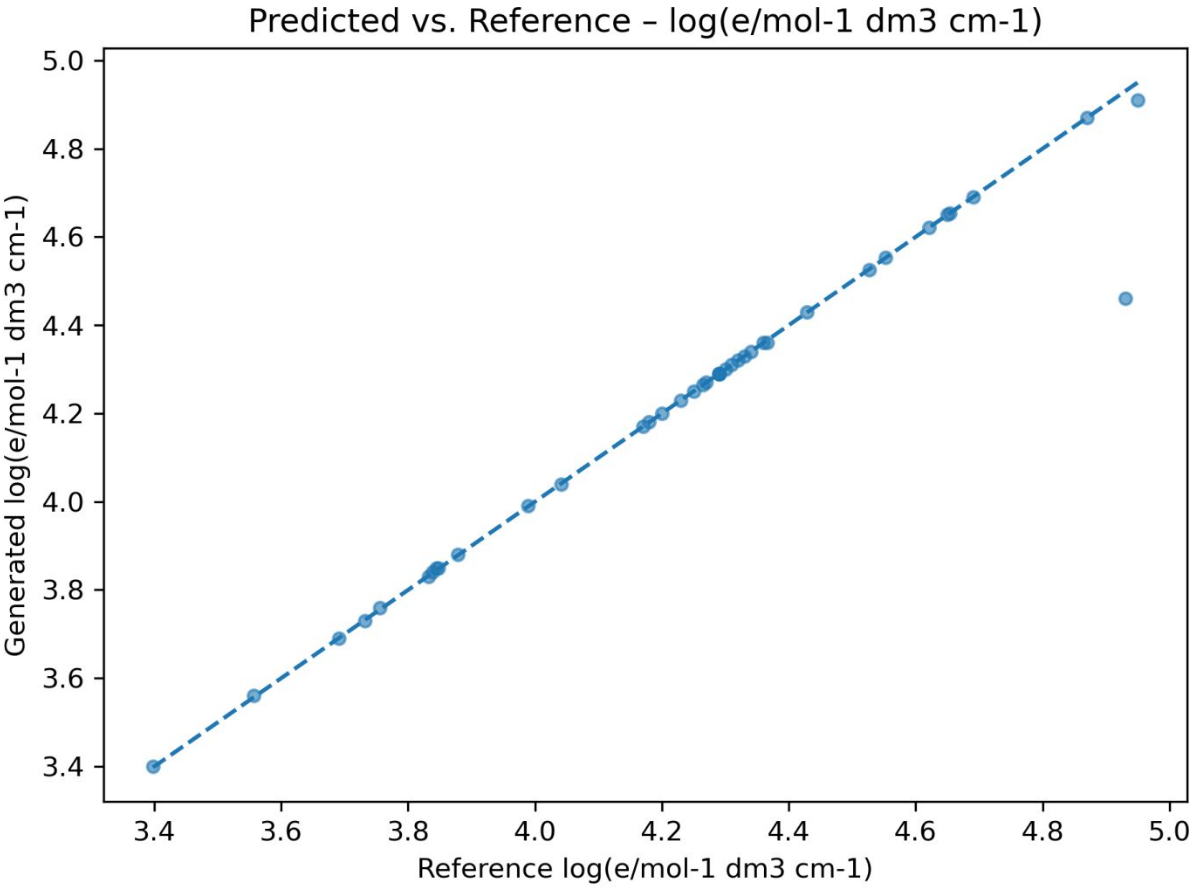
Predicted vs. Reference – log(e/mol-1 dm3 cm-1)
Generated log(e/mol-1 dm3 cm-1)
Reference log(e/mol-1 dm3 cm-1)
5.0
4.8
4.6
4.4
4.2
4.0
3.8
3.6
3.4
3.4
3.6
3.8
4.0
4.2
4.4
4.6
4.8
5.0

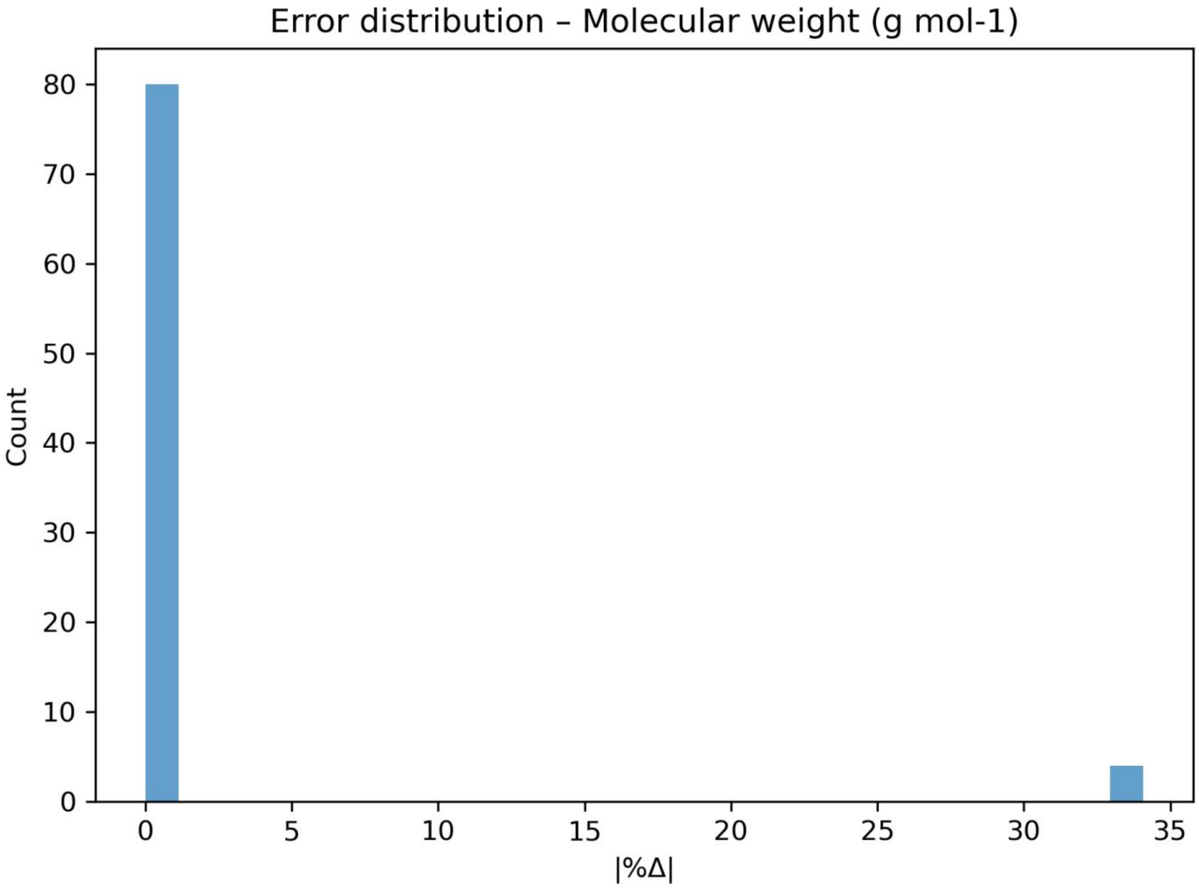

Error distribution – Molecular weight (g mol-1)
80
70
60
50
40
30
20
10
0
Count
0
5
10
15
20
25
30
35
|%Δ|

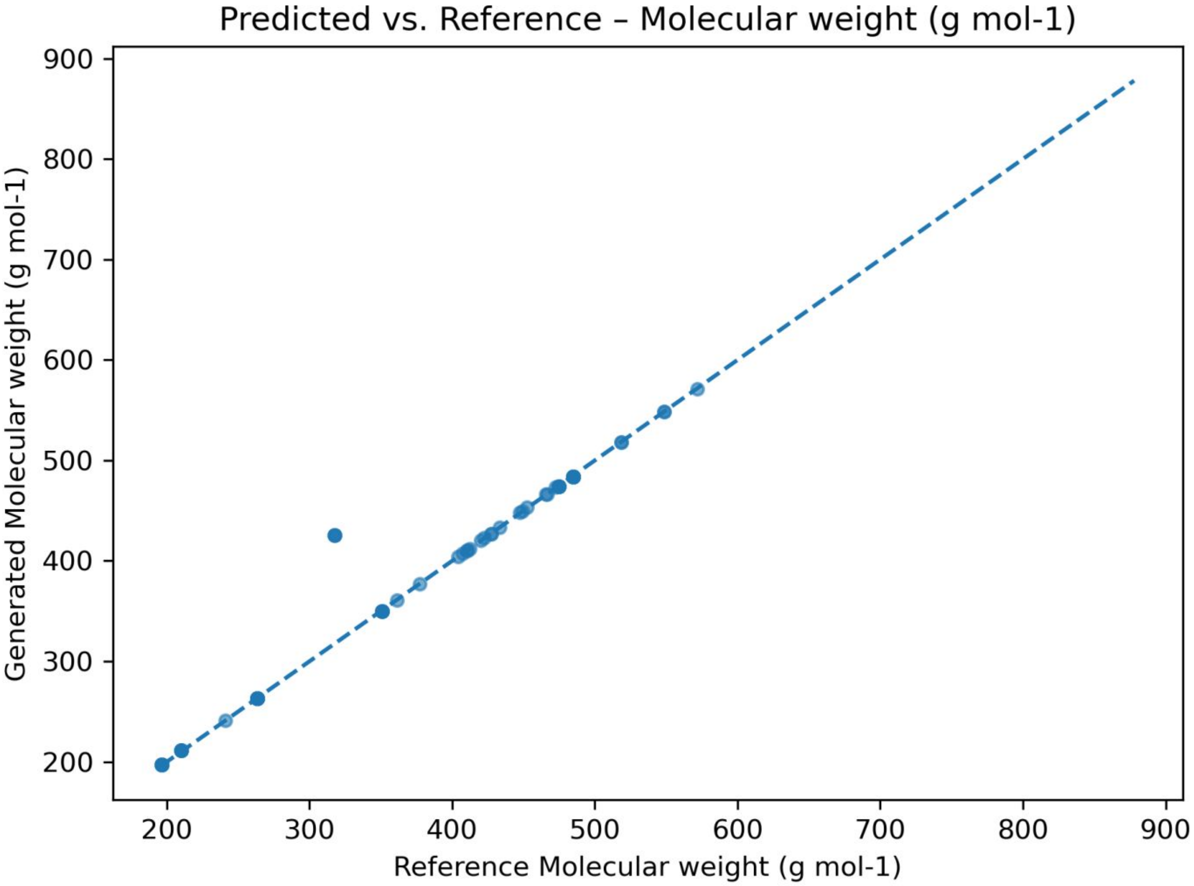
Predicted vs. Reference – Molecular weight (g mol-1)
Generated Molecular weight (g mol-1)
Reference Molecular weight (g mol-1)
900
800
700
600
500
400
300
200
200
300
400
500
600
700
800
900

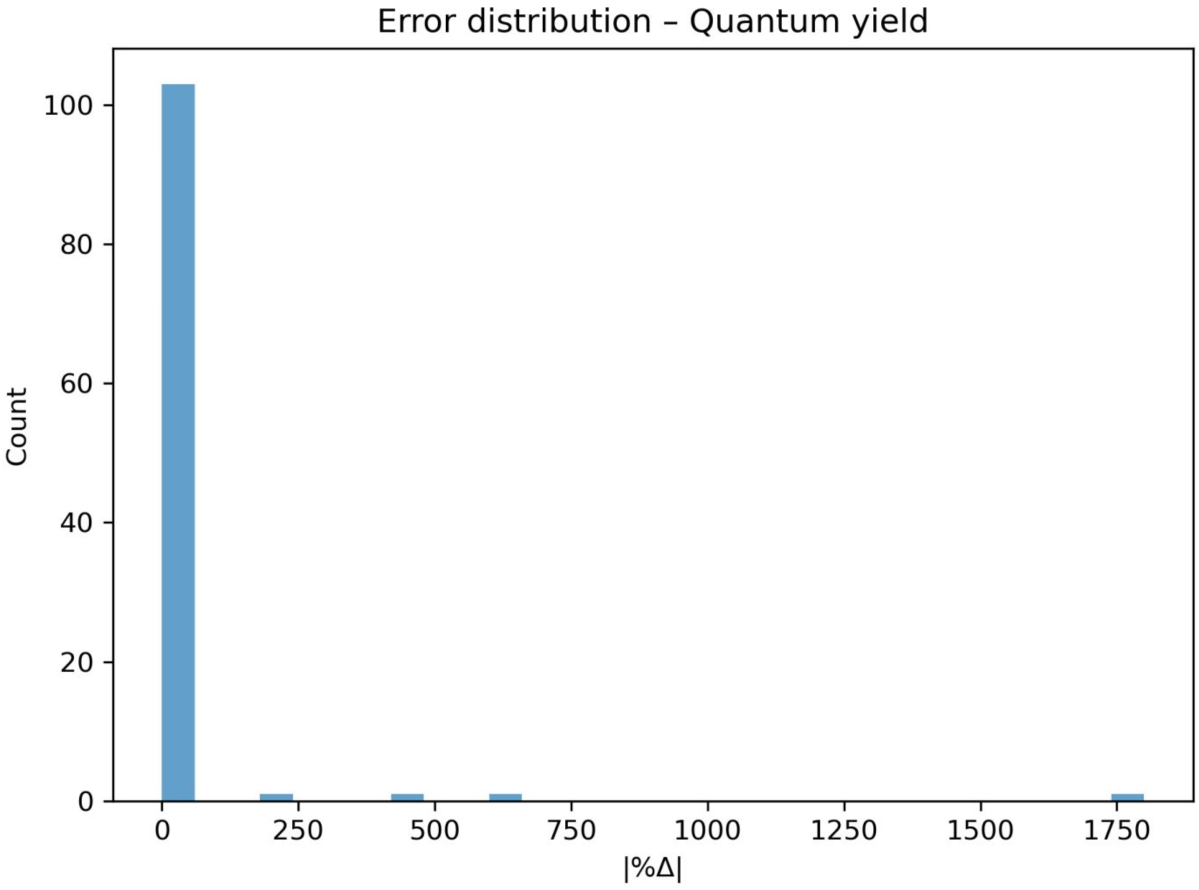
Error distribution – Quantum yield
Count
|%Δ|
0
20
40
60
80
100
0
250
500
750
1000
1250
1500
1750

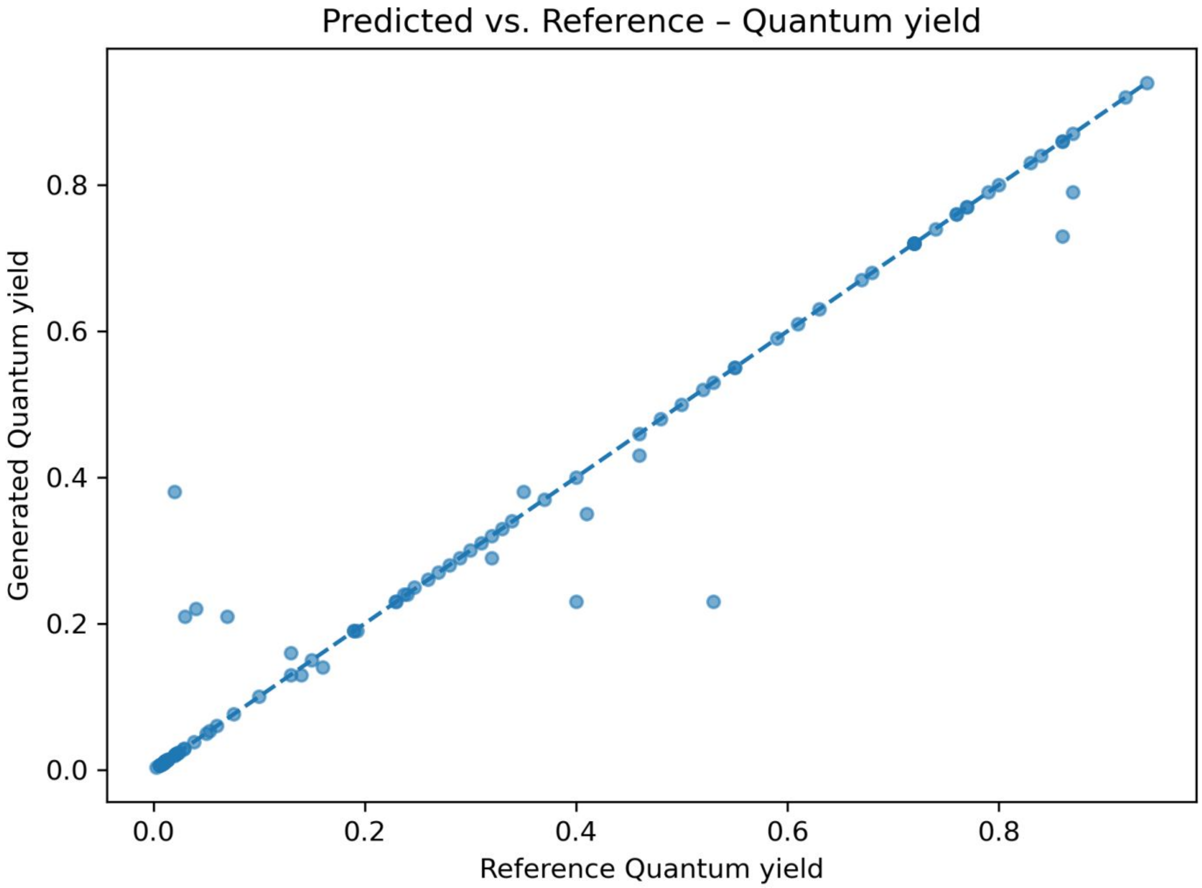
Predicted vs. Reference – Quantum yield
Generated Quantum yield
Reference Quantum yield
0.0
0.2
0.4
0.6
0.8
0.0
0.2
0.4
0.6
0.8

CDE result figures, as mentioned above:

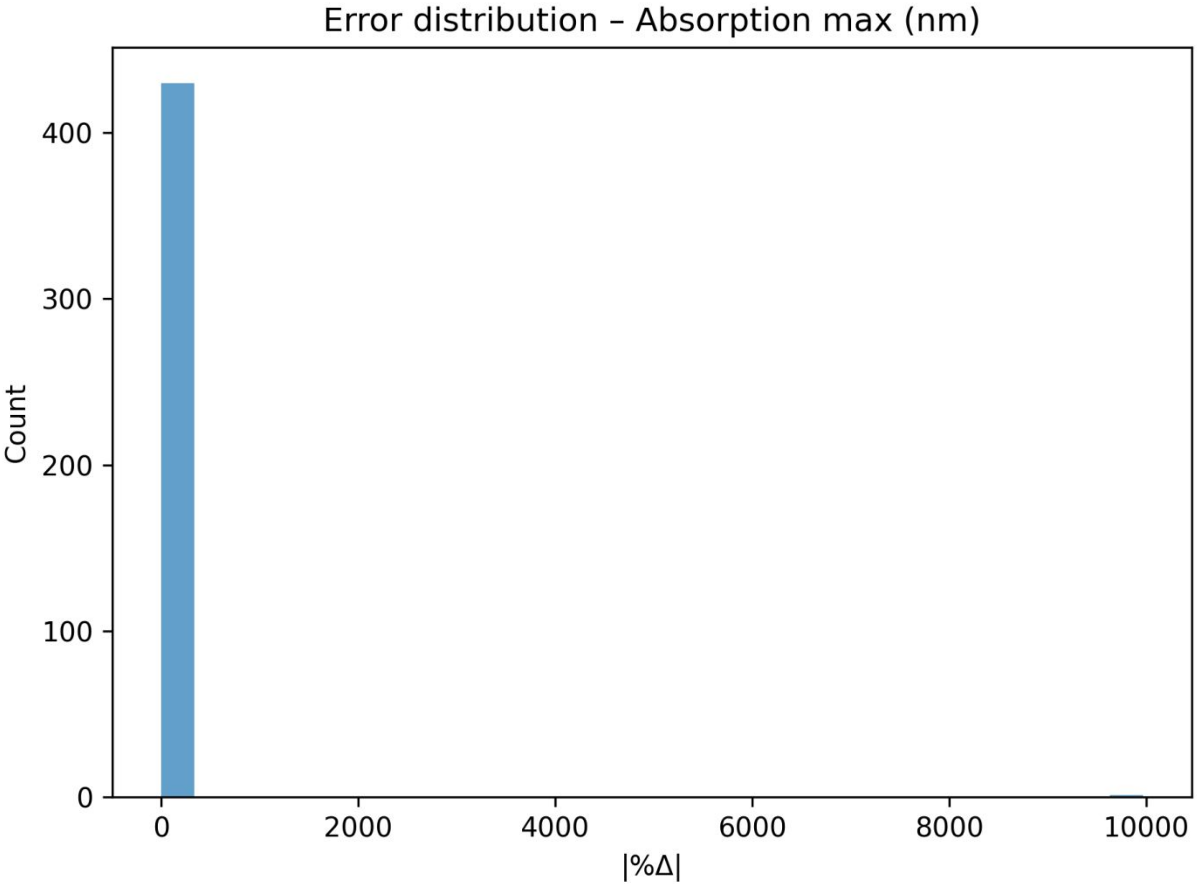

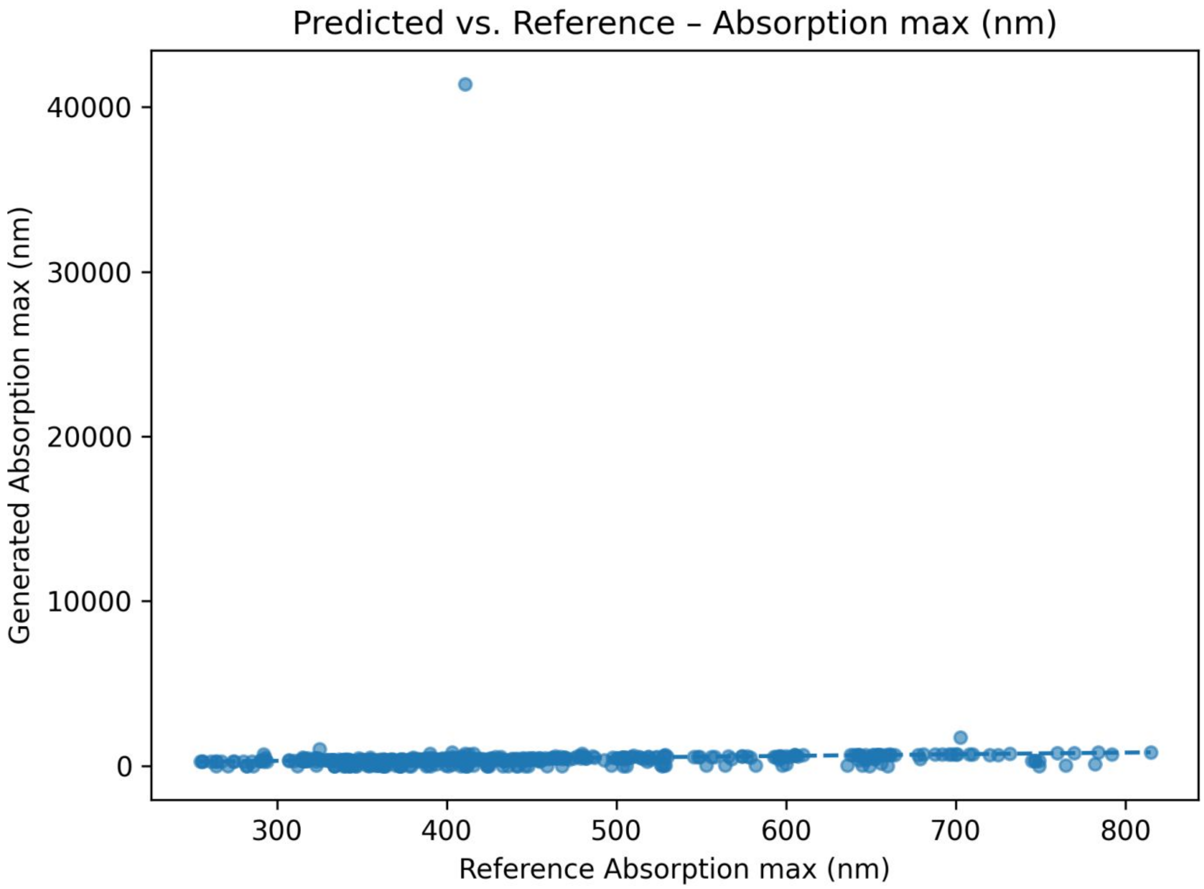

Predicted vs. Reference – Absorption max (nm)
40000
30000
20000
10000
0
300
400
500
600
700
800
Reference Absorption max (nm)
Generated Absorption max (nm)

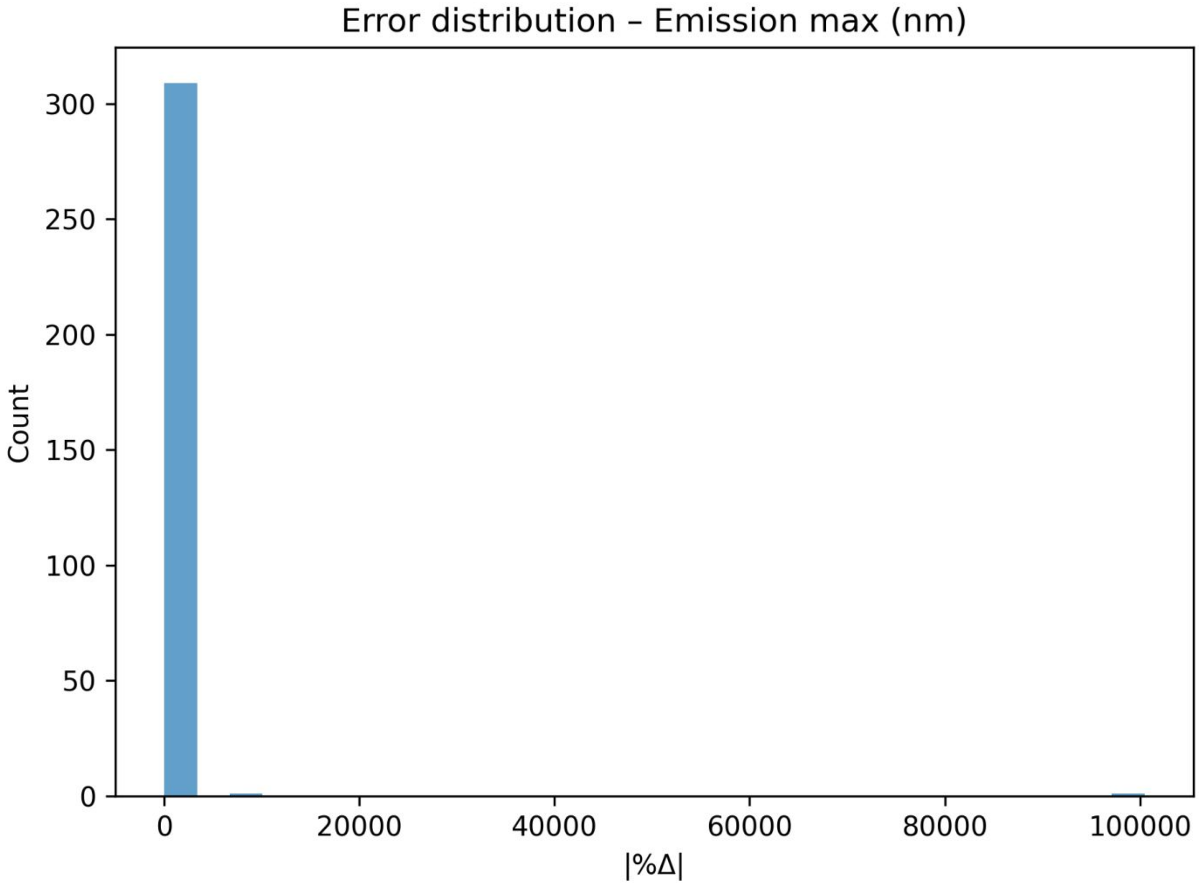
Error distribution – Emission max (nm)
Count
300
250
200
150
100
50
0
0
20000
40000
60000
80000
100000
|%Δ|

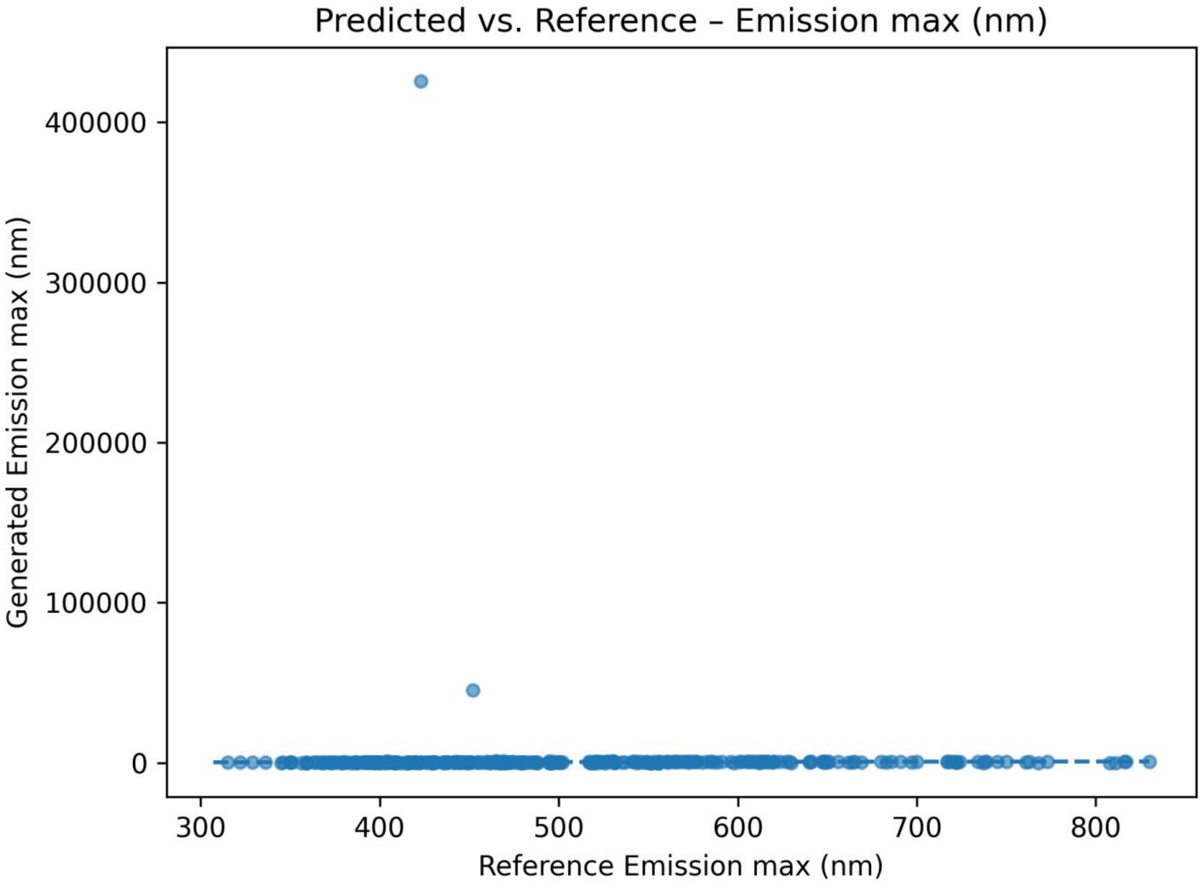

Predicted vs. Reference – Emission max (nm)
Generated Emission max (nm)
400000
300000
200000
100000
0
300
400
500
600
700
800
Reference Emission max (nm)

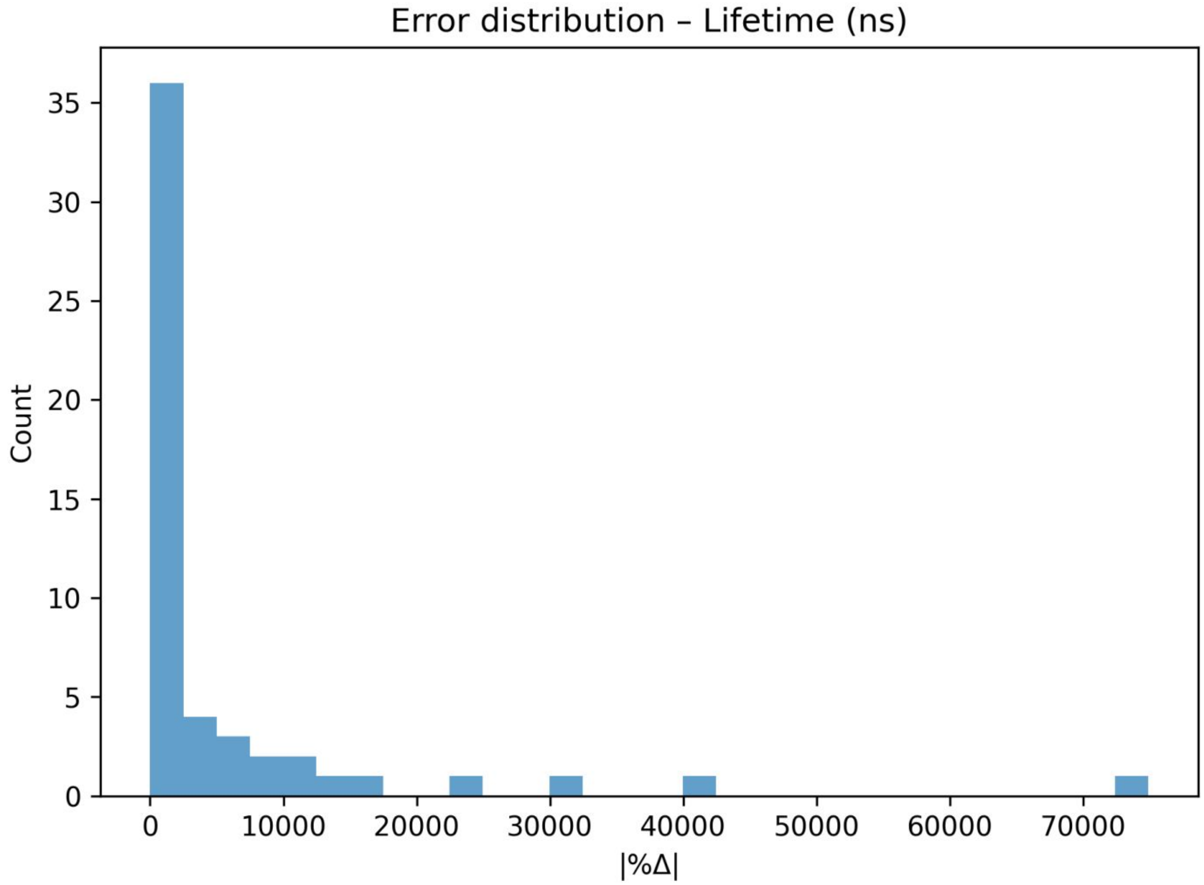
Error distribution – Lifetime (ns)
Count
|%Δ|
0
5
10
15
20
25
30
35
0
10000
20000
30000
40000
50000
60000
70000

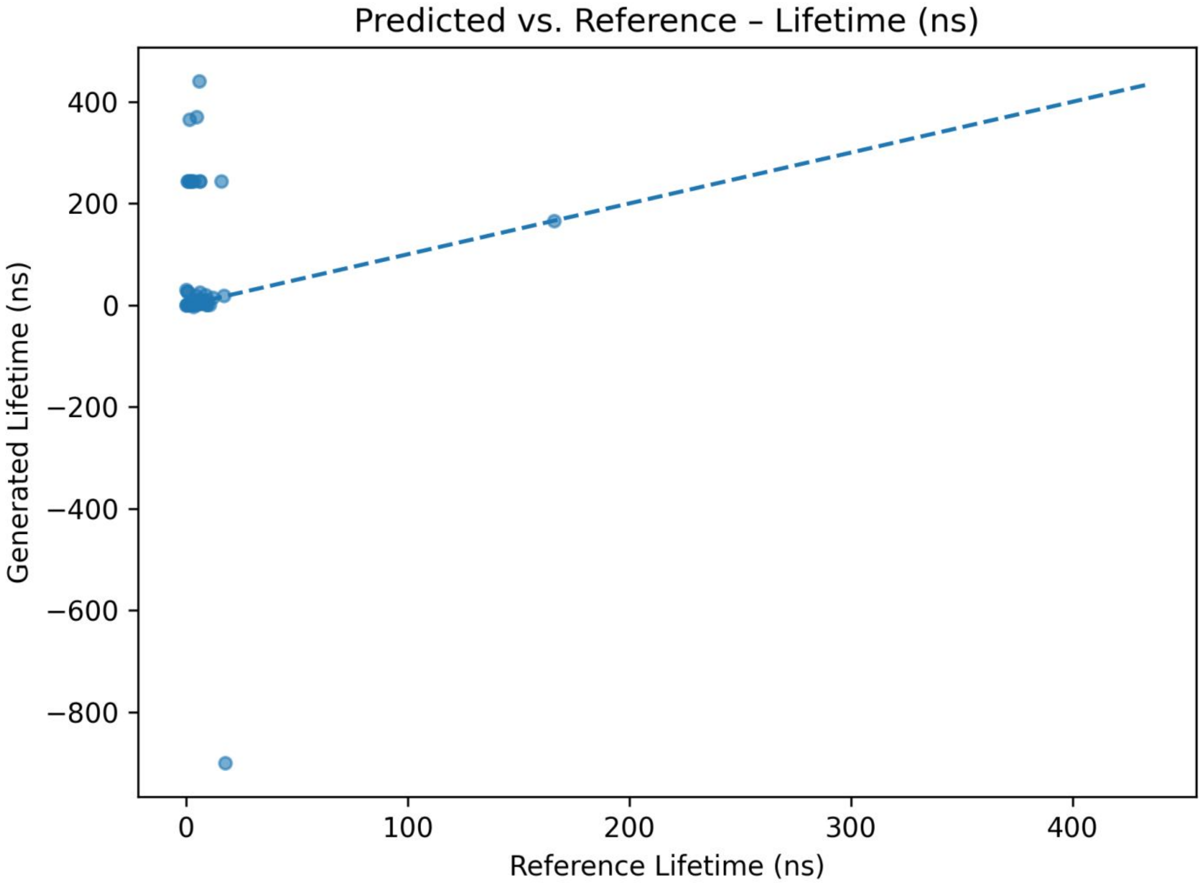

Predicted vs. Reference – Lifetime (ns)
Generated Lifetime (ns)
400
200
0
−200
−400
−600
−800
0
100
200
300
400
Reference Lifetime (ns)

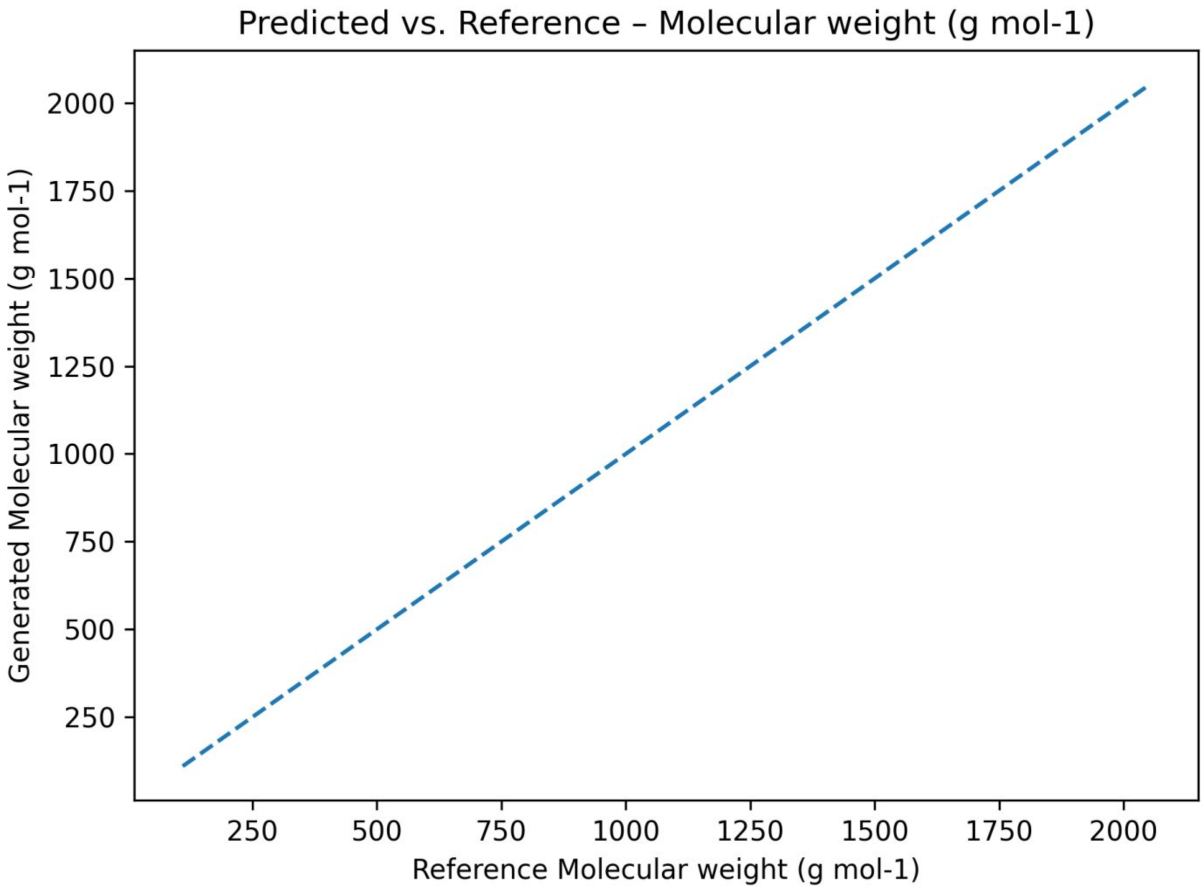

Predicted vs. Reference – Molecular weight (g mol-1)
Generated Molecular weight (g mol-1)
2000
1750
1500
1250
1000
750
500
250
250
500
750
1000
1250
1500
1750
2000
Reference Molecular weight (g mol-1)

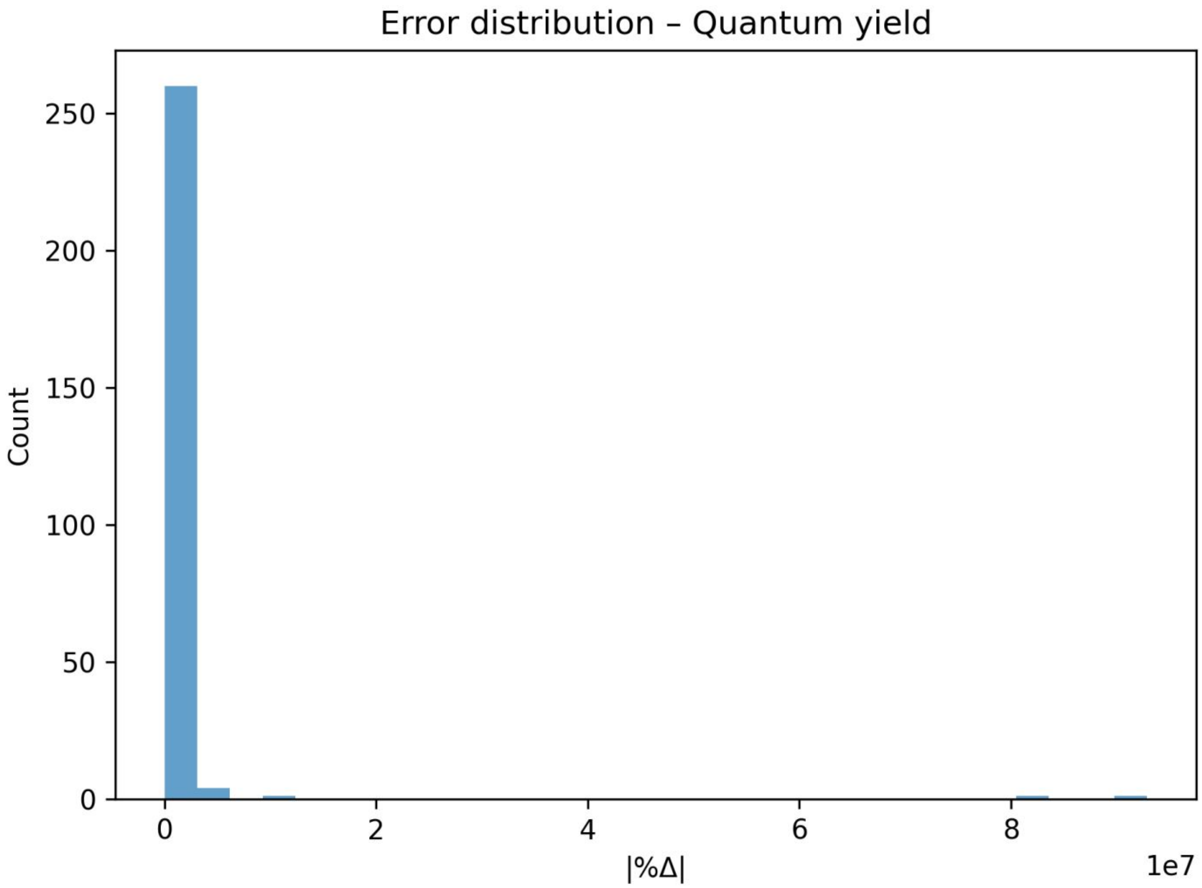
Error distribution – Quantum yield
250
200
150
100
50
0
Count
0
2
4
6
8
1e7
|%Δ|

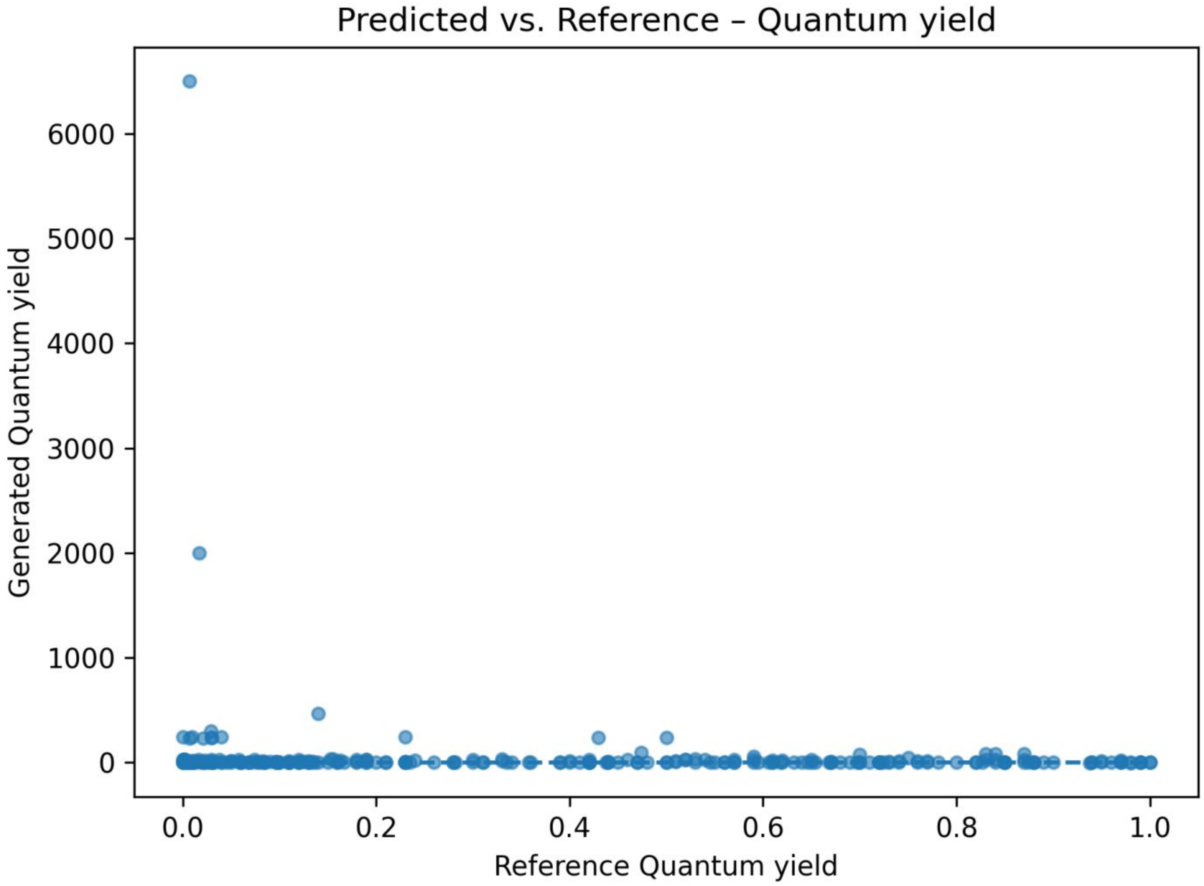
Predicted vs. Reference – Quantum yield
Generated Quantum yield
Reference Quantum yield
0
1000
2000
3000
4000
5000
6000
0.0
0.2
0.4
0.6
0.8
1.0

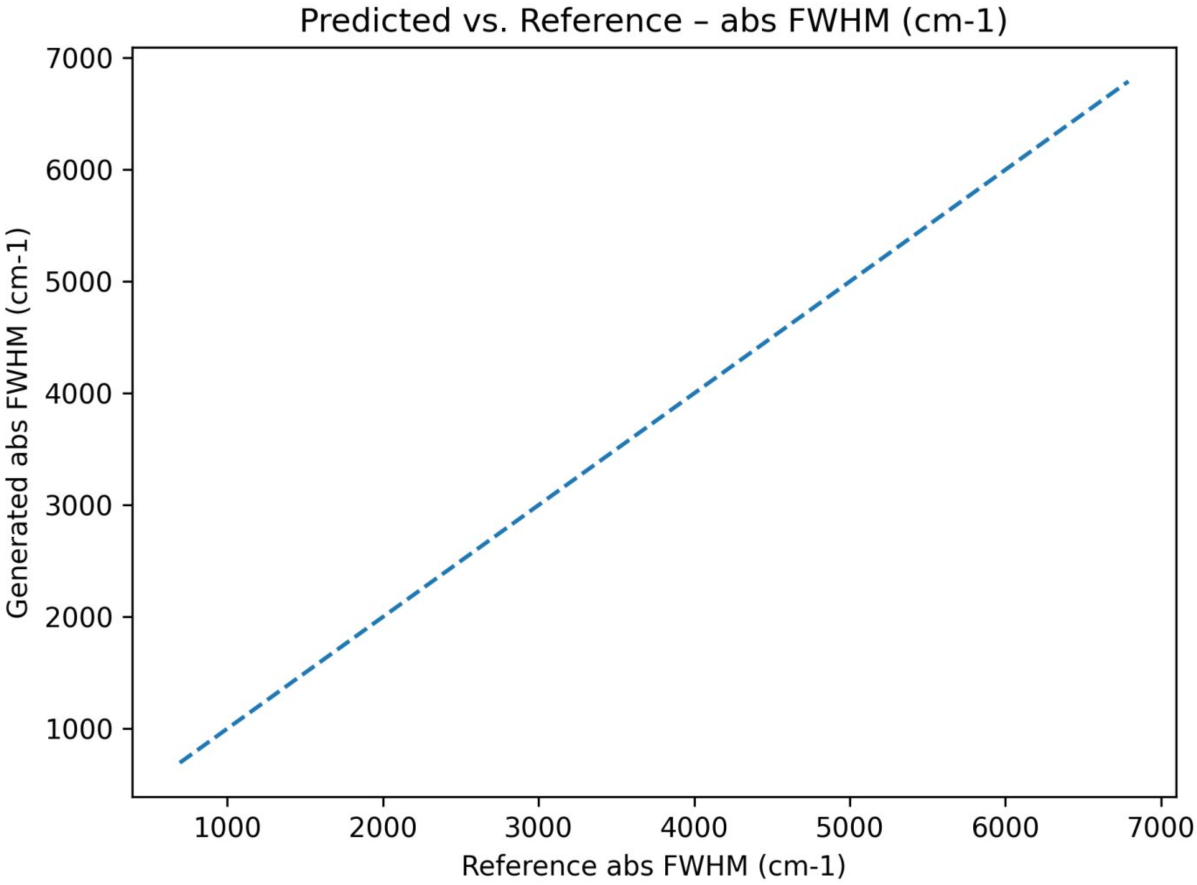

Predicted vs. Reference – abs FWHM (cm-1)
7000
6000
5000
4000
3000
2000
1000
Generated abs FWHM (cm-1)
1000
2000
3000
4000
5000
6000
7000
Reference abs FWHM (cm-1)

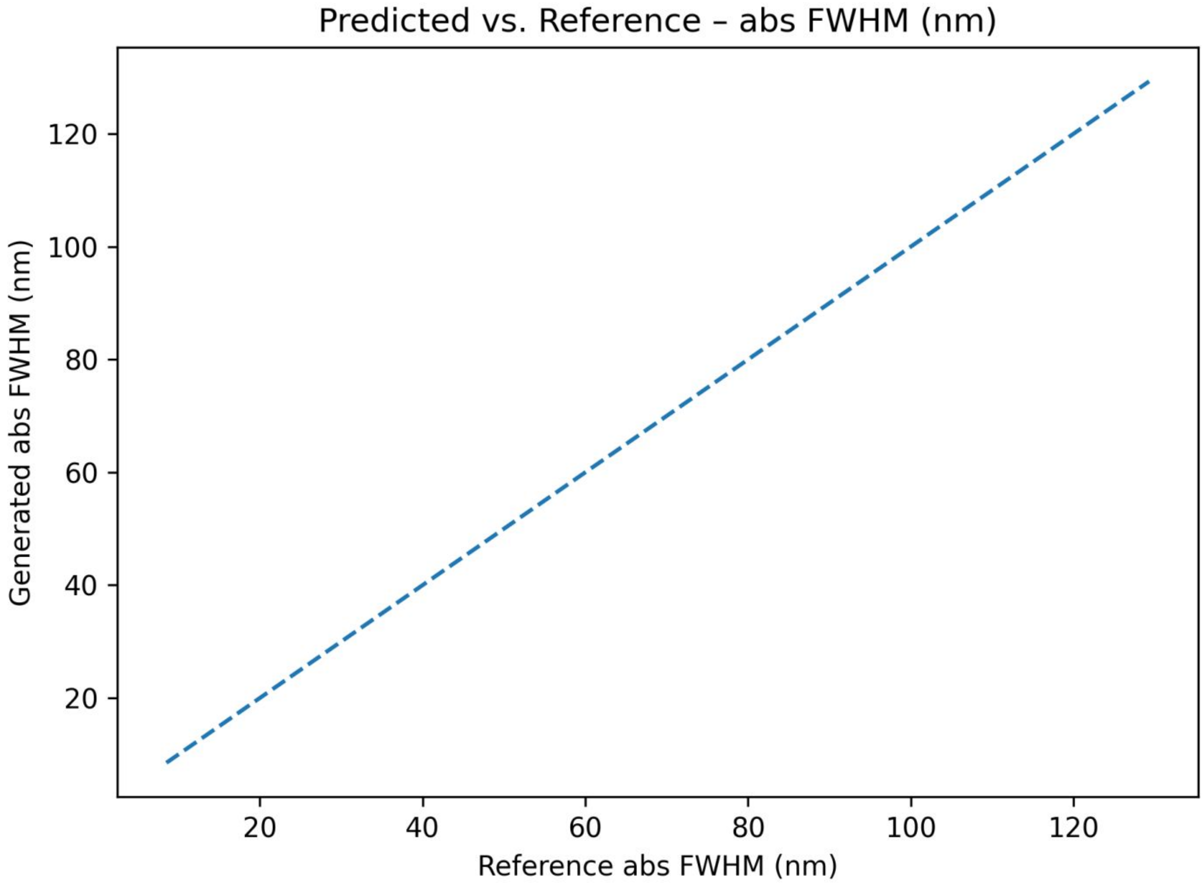
Predicted vs. Reference – abs FWHM (nm)
Generated abs FWHM (nm)
Reference abs FWHM (nm)
20
40
60
80
100
120
20
40
60
80
100
120

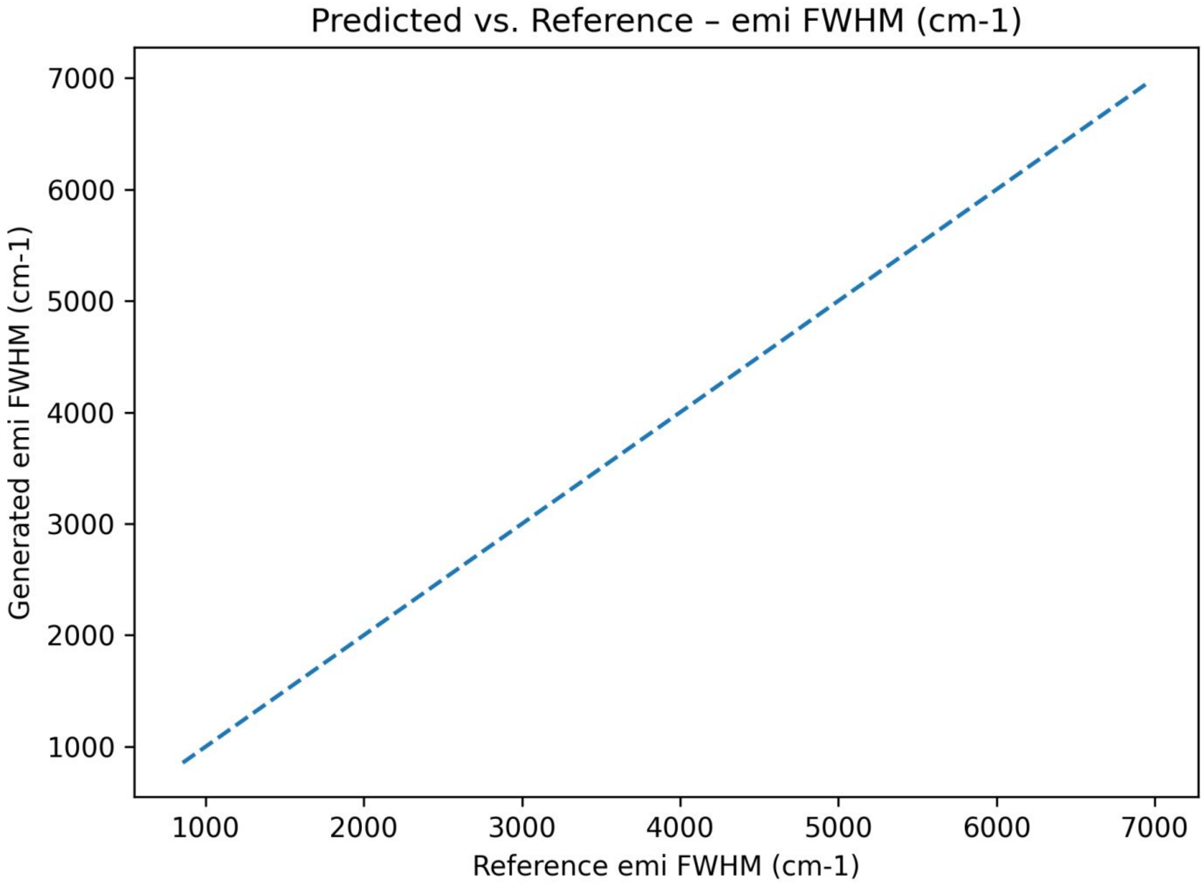
Predicted vs. Reference – emi FWHM (cm-1)
7000
6000
5000
4000
3000
2000
1000
Generated emi FWHM (cm-1)
1000
2000
3000
4000
5000
6000
7000
Reference emi FWHM (cm-1)

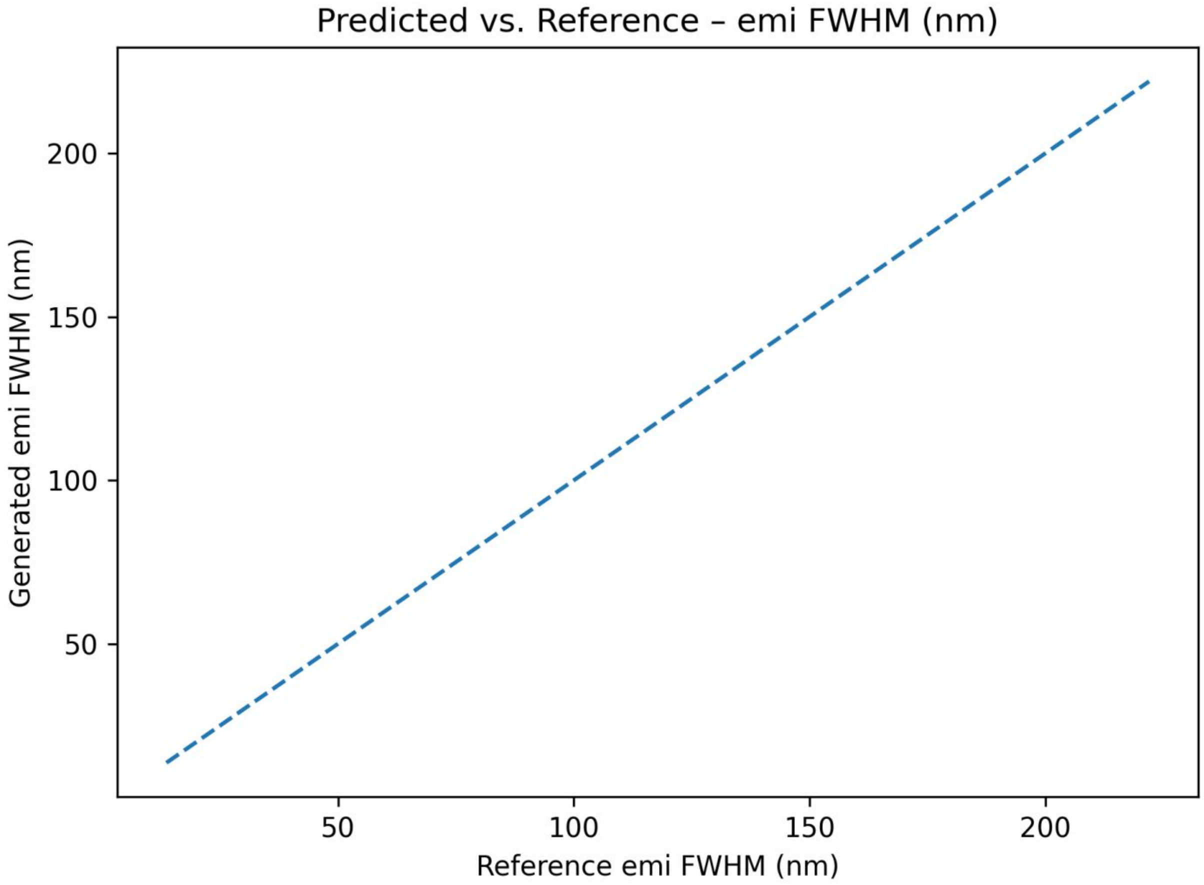
Predicted vs. Reference – emi FWHM (nm)
Generated emi FWHM (nm)
Reference emi FWHM (nm)
50
100
150
200

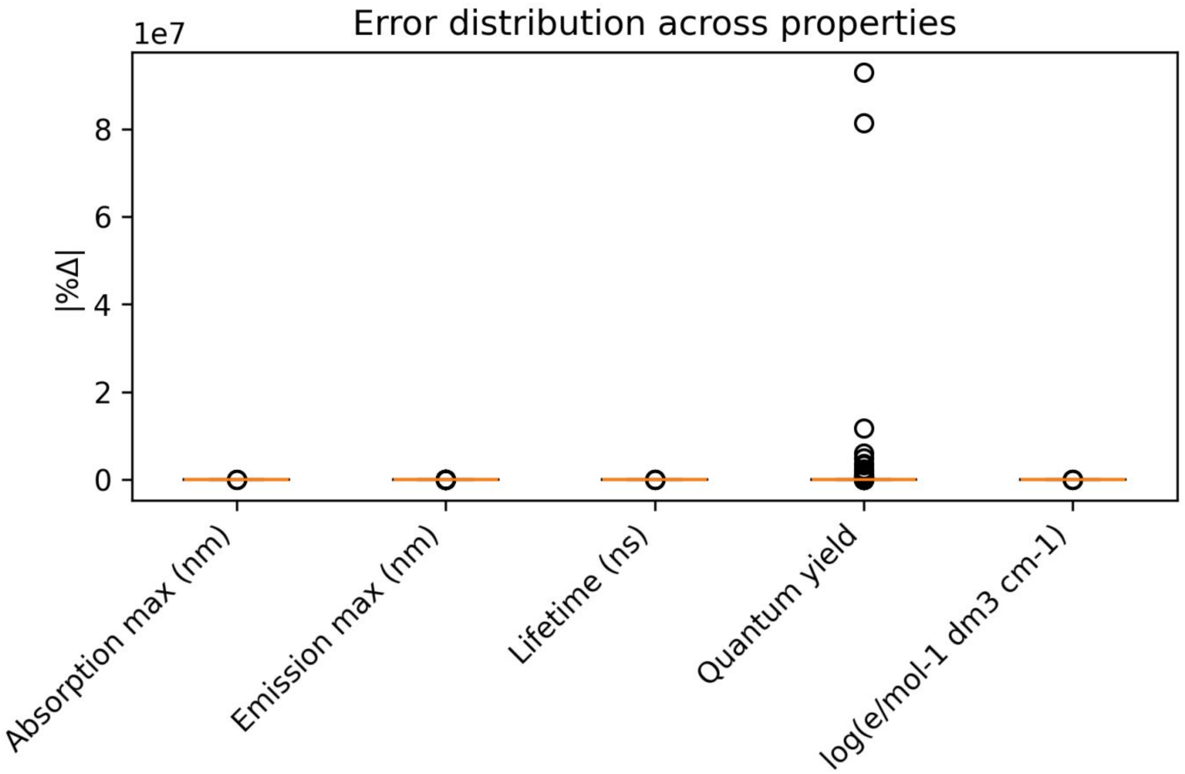
Error distribution across properties
1e7
8
6
4
2
0
|%Δ|
Absorption max (nm)
Emission max (nm)
Lifetime (ns)
Quantum yield
log(e/mol-1 dm3 cm-1)

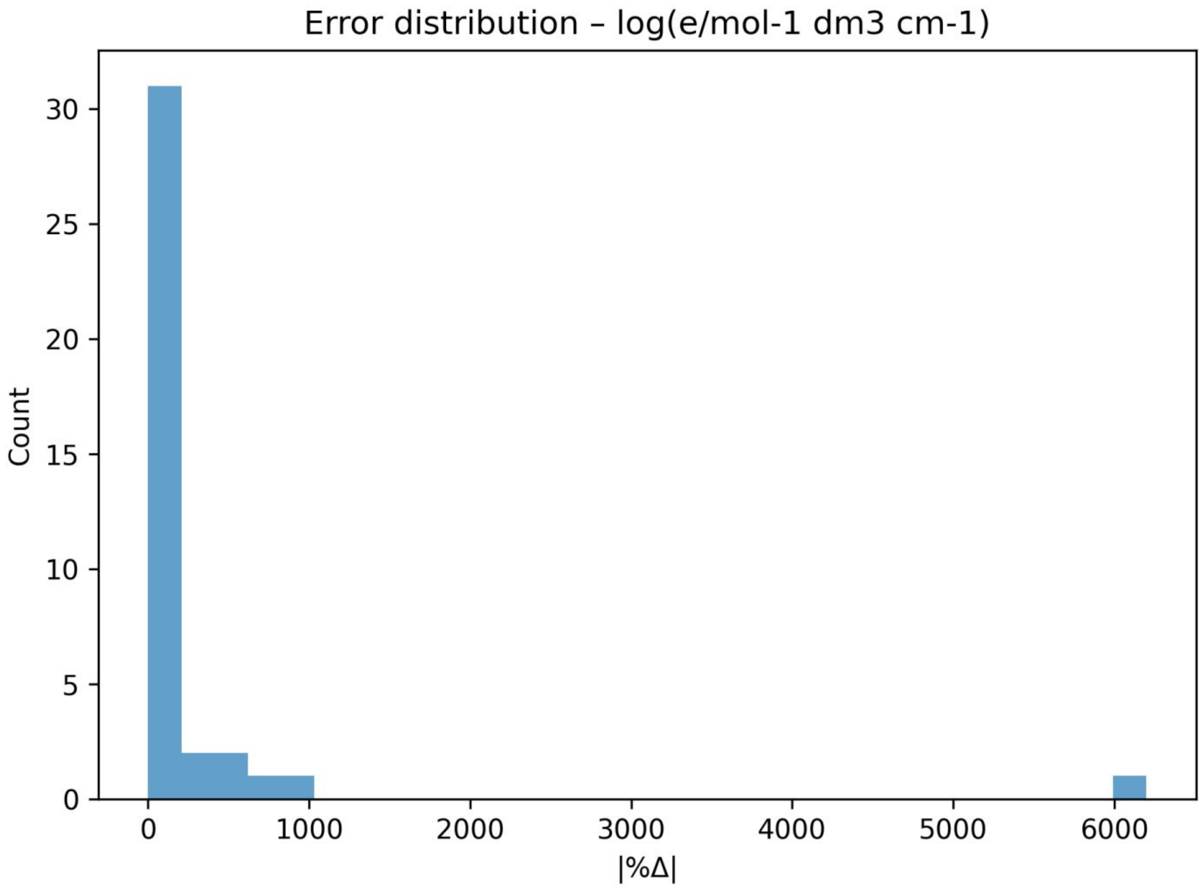
Error distribution – log(e/mol-1 dm3 cm-1)
Count
30
25
20
15
10
5
0
0
1000
2000
3000
4000
5000
6000
|%Δ|

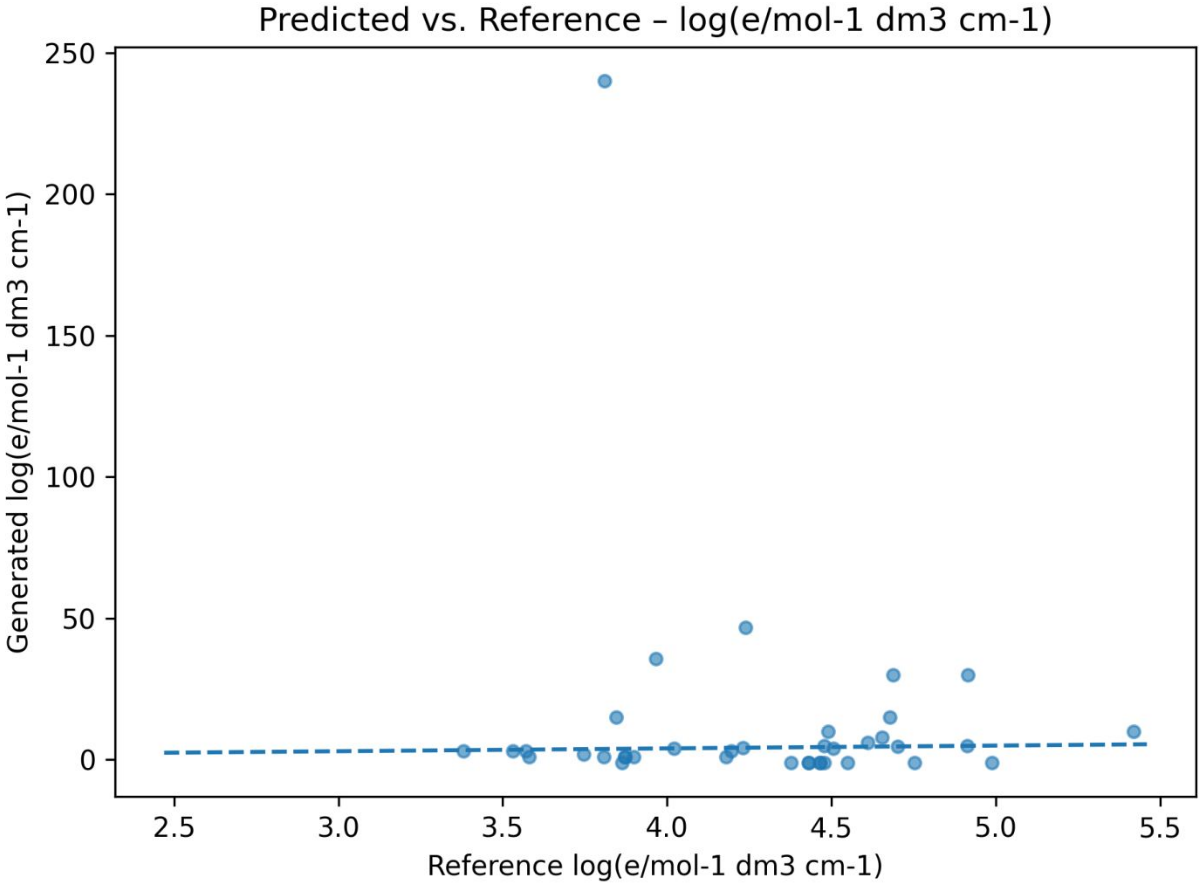
Predicted vs. Reference – log(e/mol-1 dm3 cm-1)
Generated log(e/mol-1 dm3 cm-1)
Reference log(e/mol-1 dm3 cm-1)
250
200
150
100
50
0
2.5
3.0
3.5
4.0
4.5
5.0
5.5

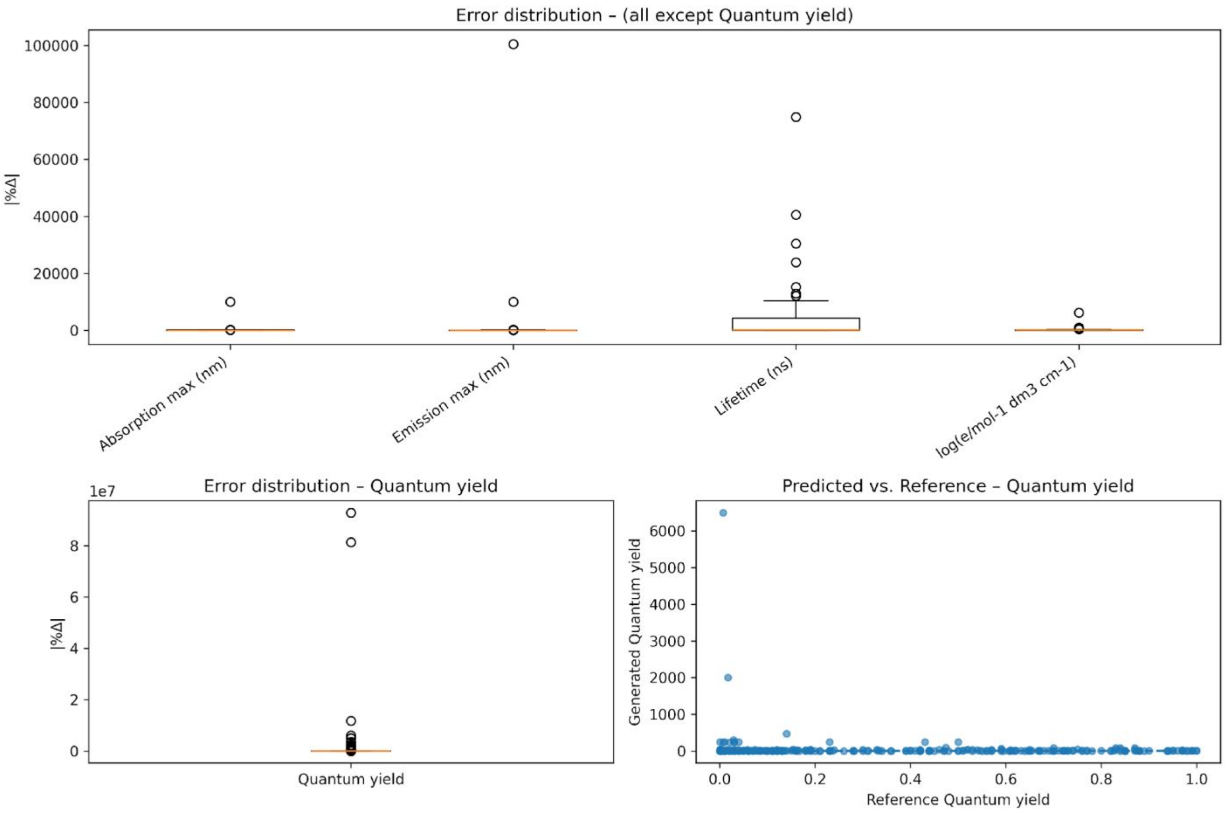
Error distribution – (all except Quantum yield)
|%Δ|
100000
80000
60000
40000
20000
0
Absorption max (nm)
Emission max (nm)
Lifetime (ns)
log(e/mol-1 dm3 cm-1)
Error distribution – Quantum yield
1e7
8
6
4
2
0
Quantum yield
Predicted vs. Reference – Quantum yield
Generated Quantum yield
6000
5000
4000
3000
2000
1000
0
0.0
0.2
0.4
0.6
0.8
1.0
Reference Quantum yield

CDE_fair result figures, as mentioned above:

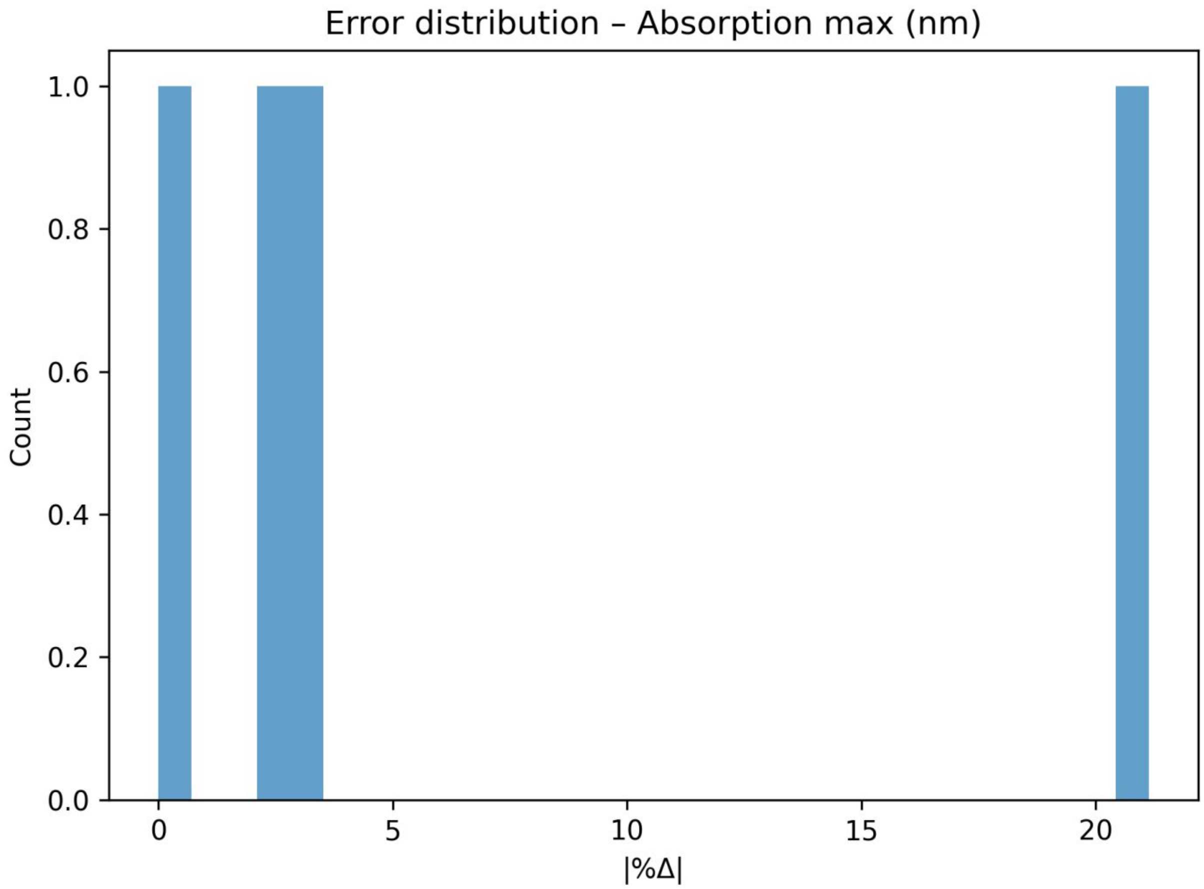

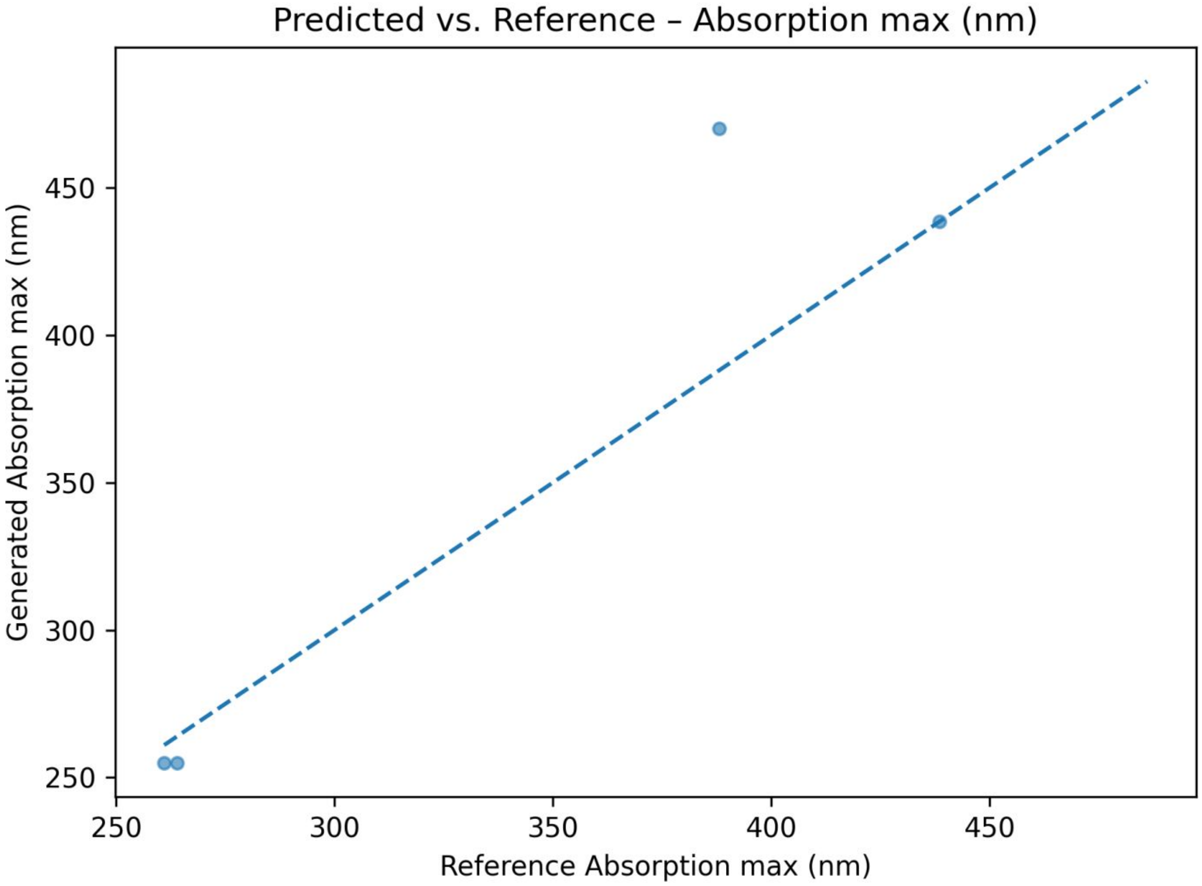
Predicted vs. Reference – Absorption max (nm)
Generated Absorption max (nm)
Reference Absorption max (nm)
250
300
350
400
450
250
300
350
400
450

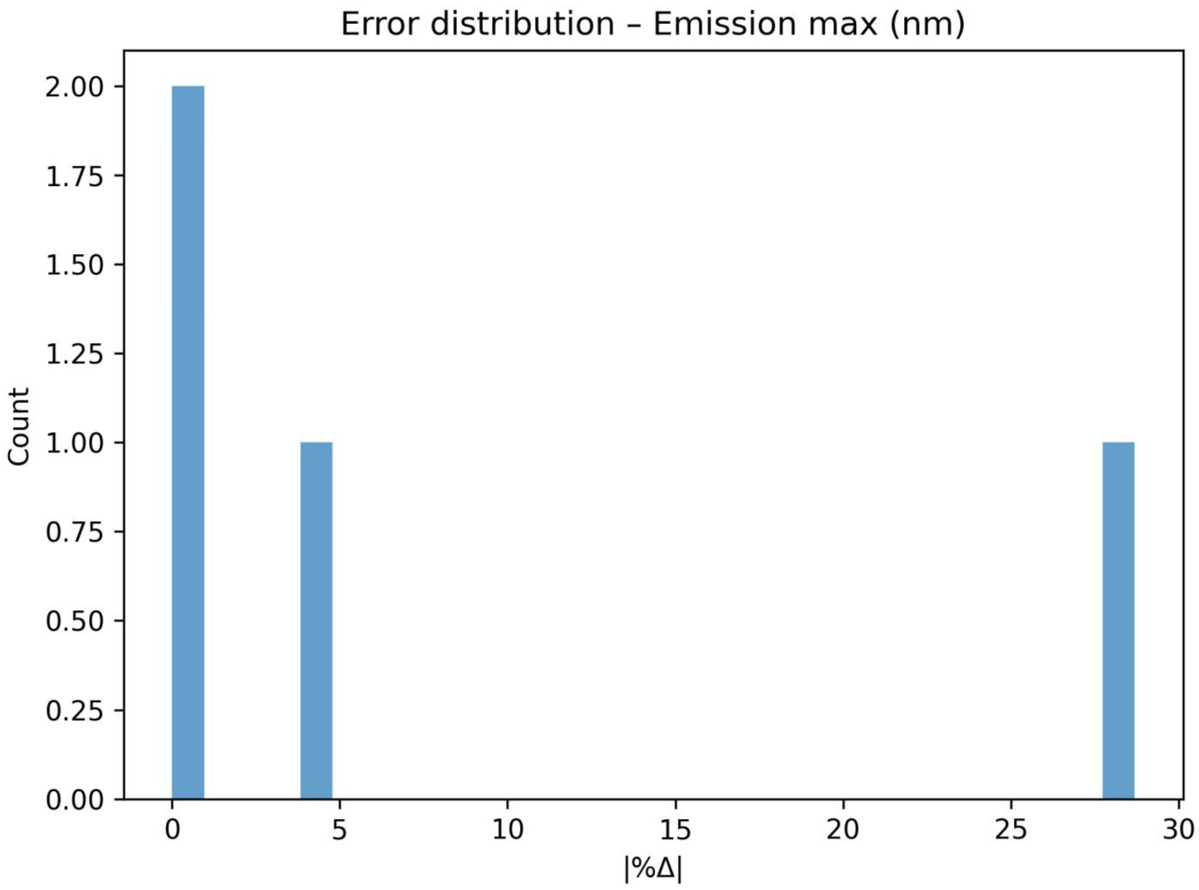

Error distribution – Emission max (nm)
Count
2.00
1.75
1.50
1.25
1.00
0.75
0.50
0.25
0.00
0
5
10
15
20
25
30
|%Δ|

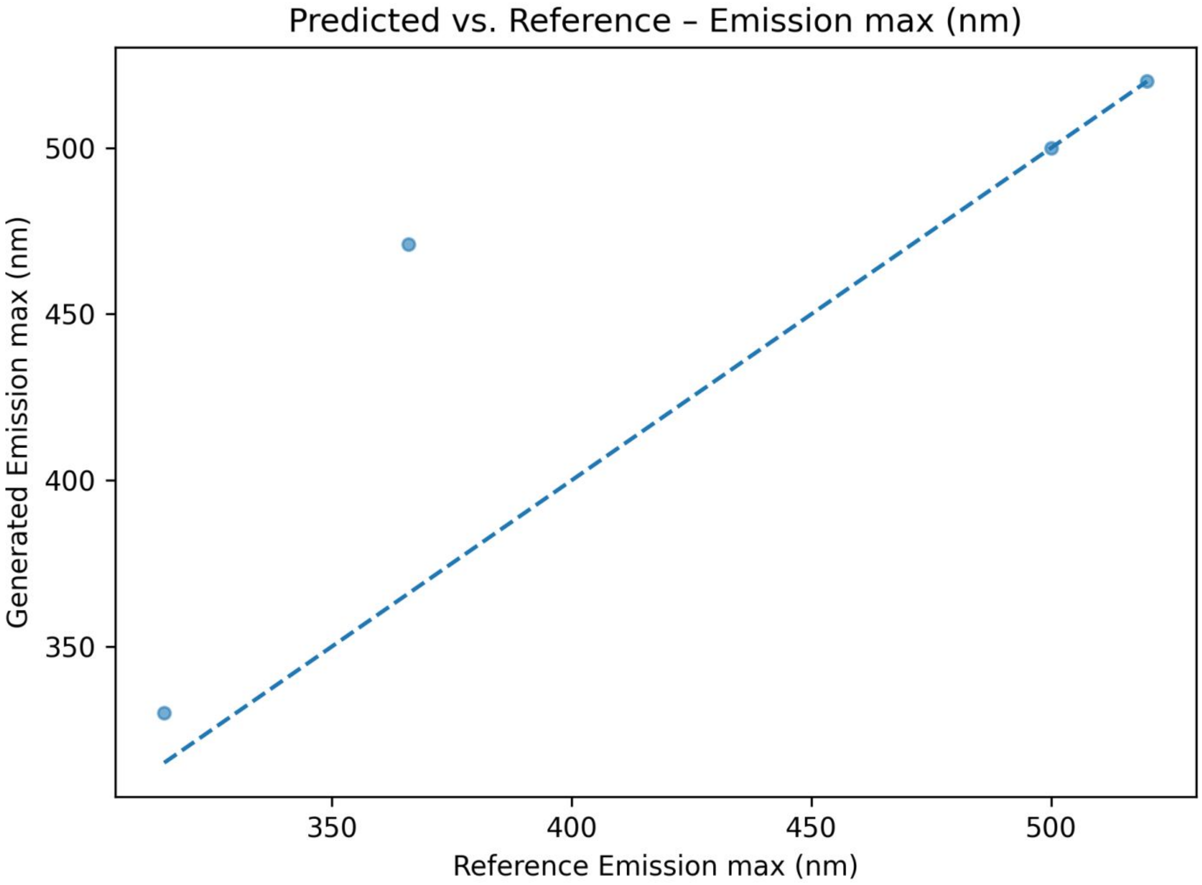

Predicted vs. Reference – Emission max (nm)
500
450
400
350
Generated Emission max (nm)
350
400
450
500
Reference Emission max (nm)

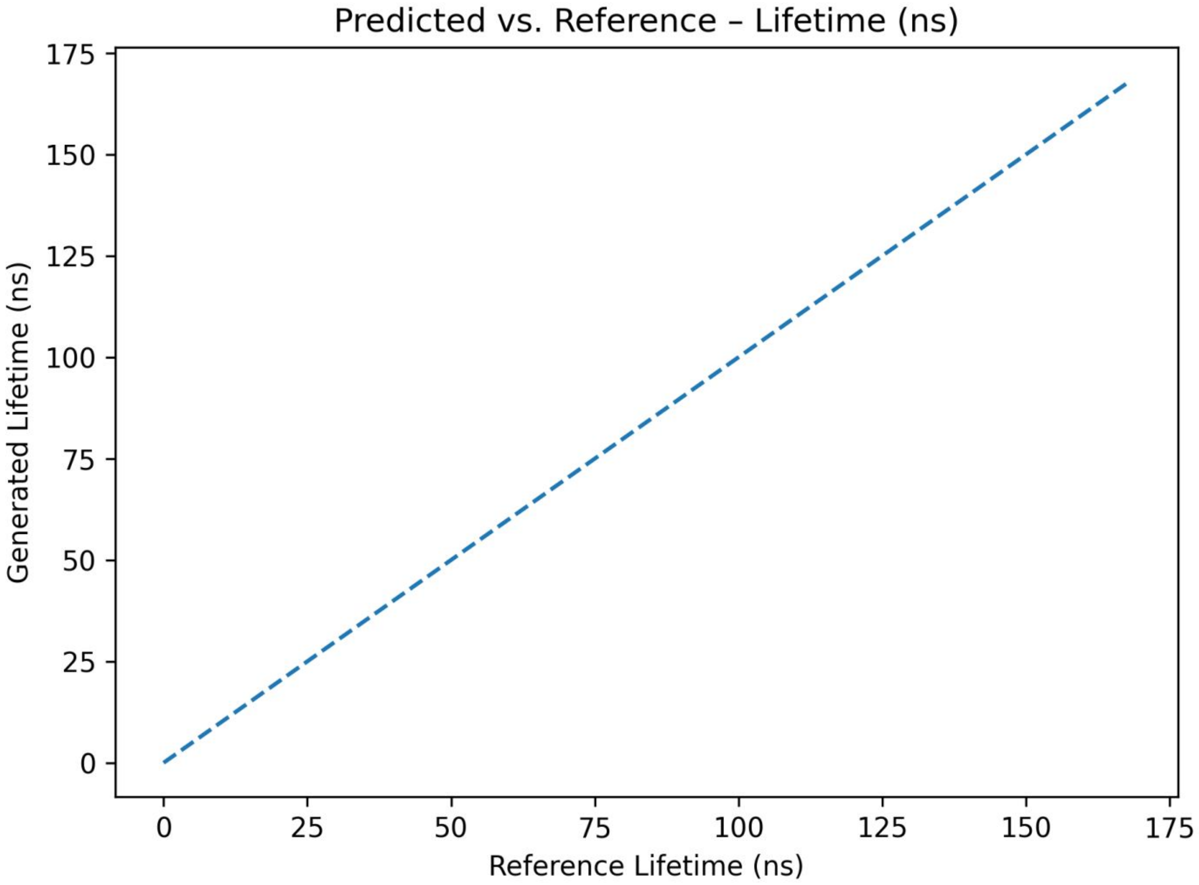
Predicted vs. Reference – Lifetime (ns)
Generated Lifetime (ns)
Reference Lifetime (ns)
0
25
50
75
100
125
150
175

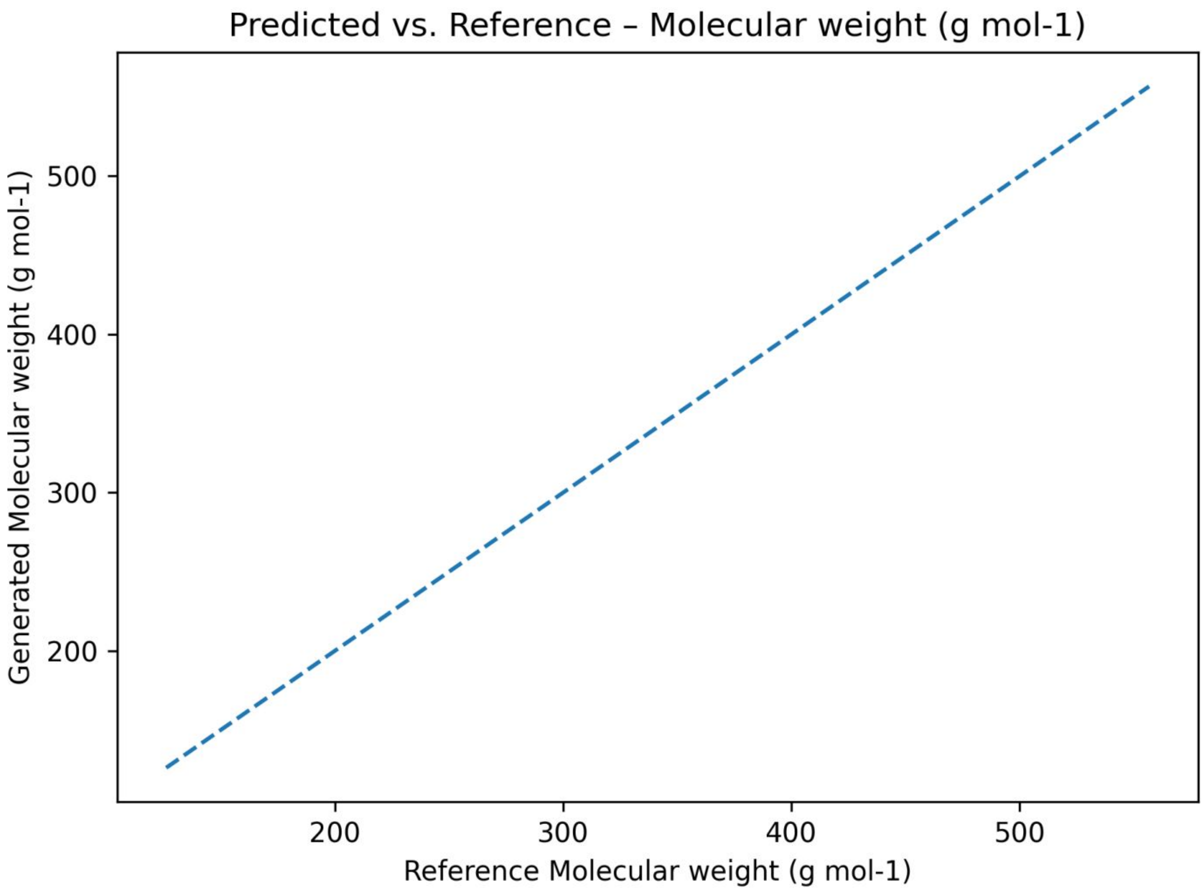
Predicted vs. Reference – Molecular weight (g mol-1)
Generated Molecular weight (g mol-1)
500
400
300
200
200
300
400
500
Reference Molecular weight (g mol-1)

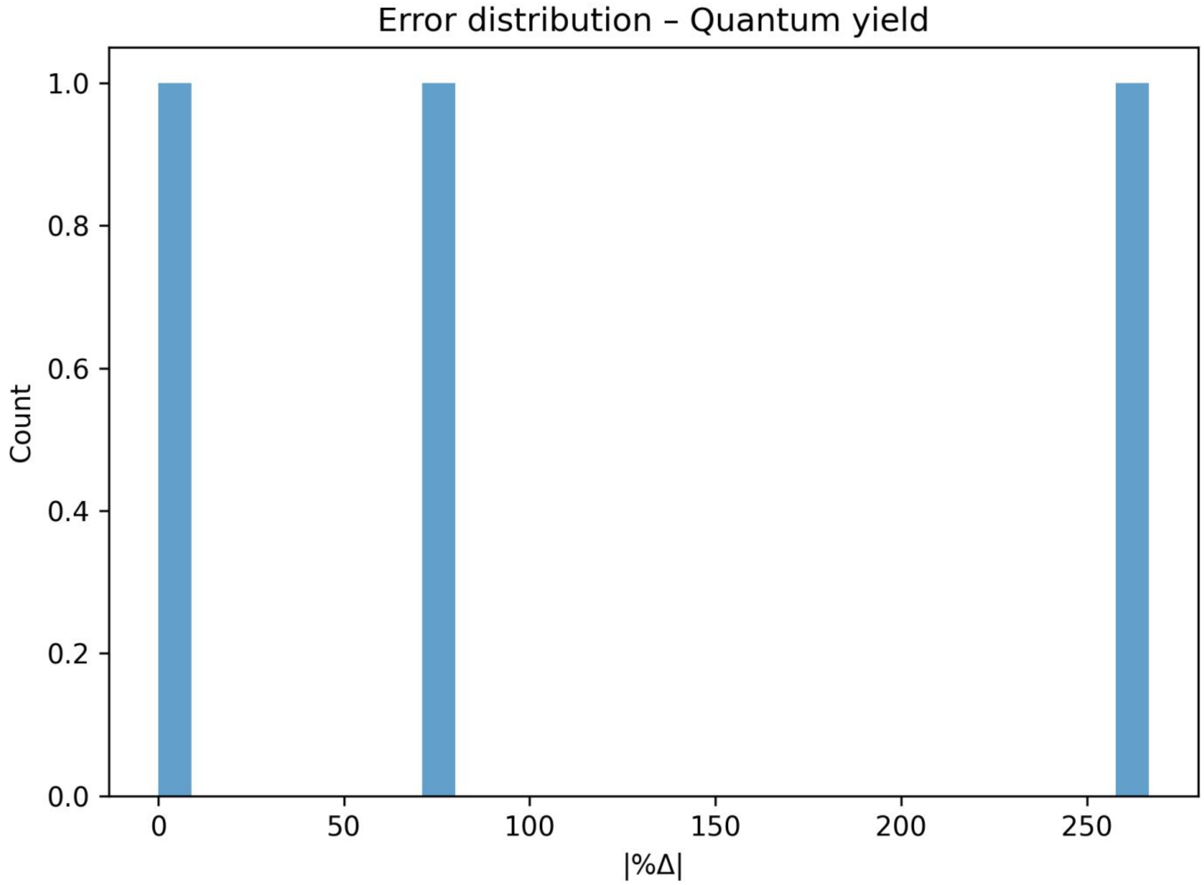
Error distribution – Quantum yield
1.0
0.8
0.6
0.4
0.2
0.0
Count
0
50
100
150
200
250
|%Δ|

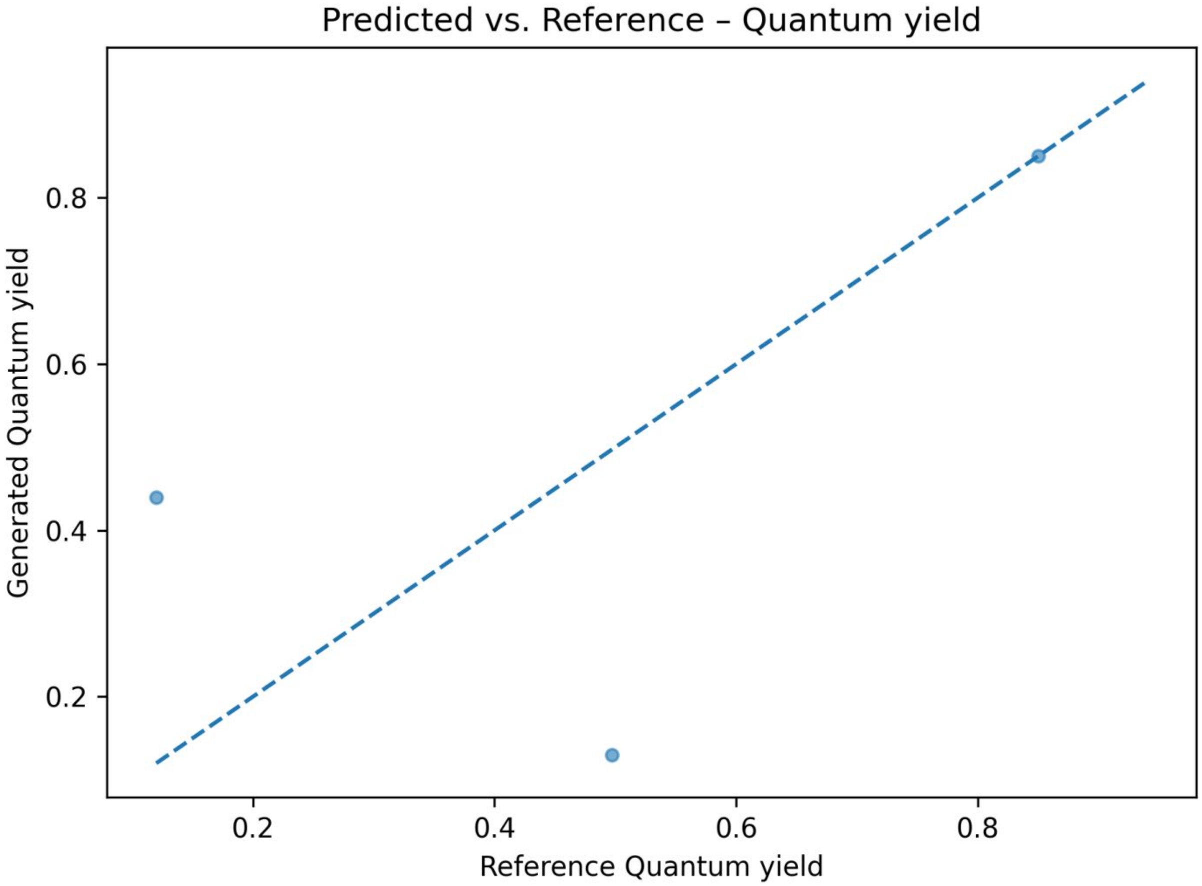

Predicted vs. Reference – Quantum yield
Generated Quantum yield
Reference Quantum yield
0.2
0.4
0.6
0.8
0.2
0.4
0.6
0.8

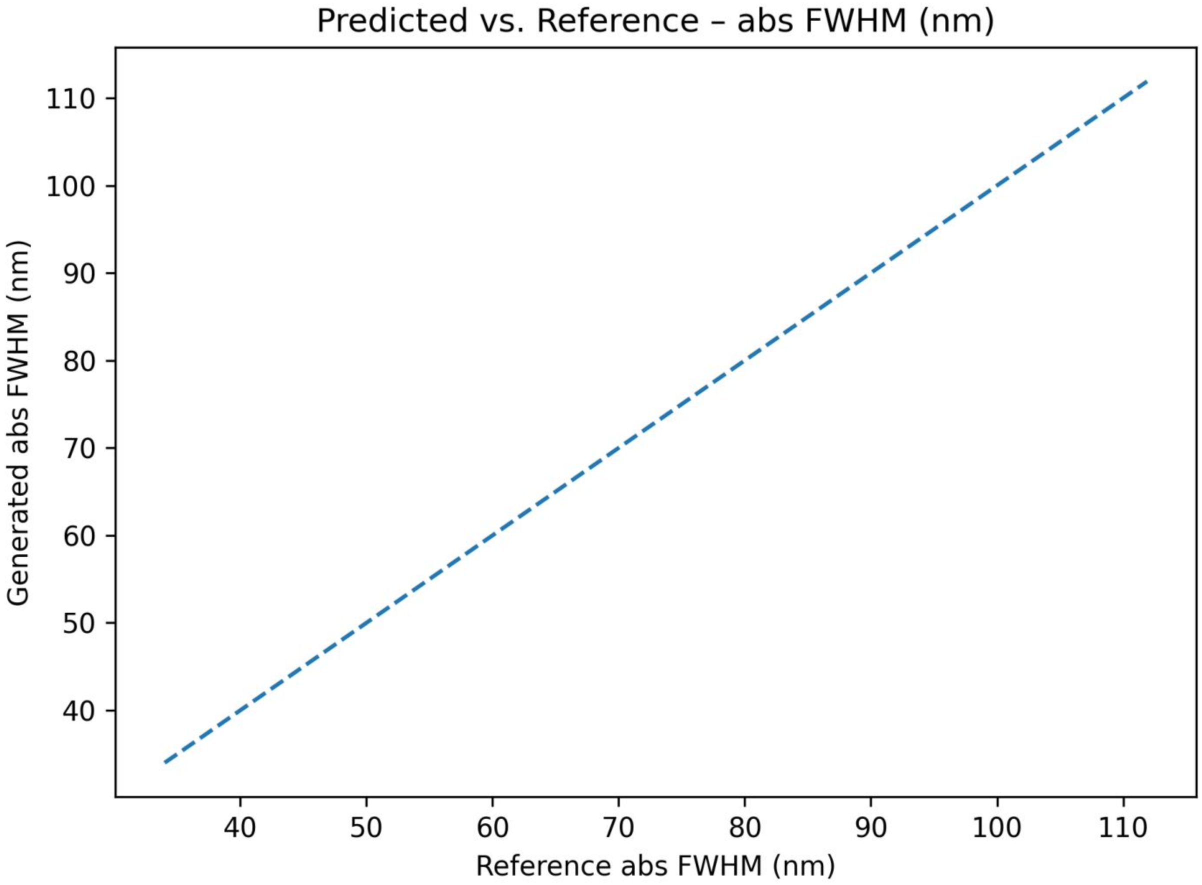
Predicted vs. Reference – abs FWHM (nm)
Generated abs FWHM (nm)
110
100
90
80
70
60
50
40
Reference abs FWHM (nm)
40
50
60
70
80
90
100
110

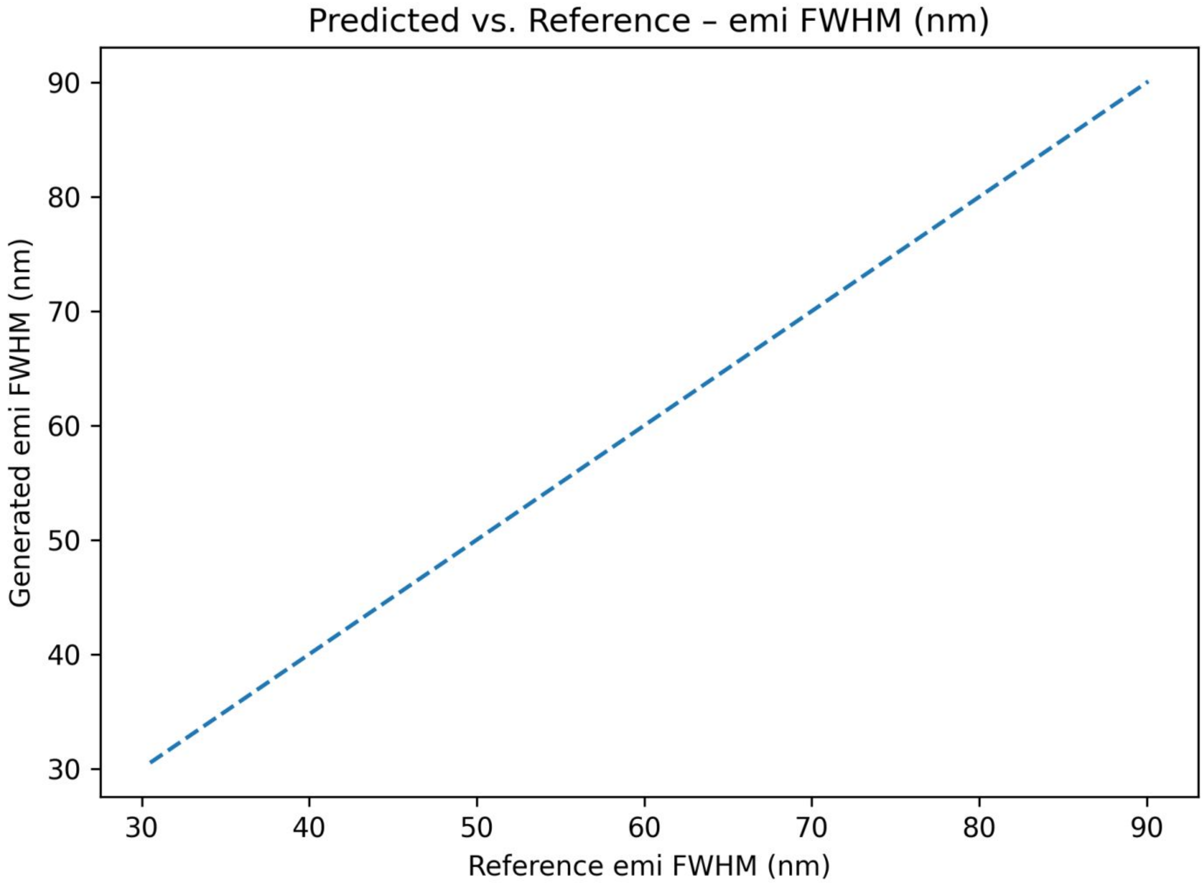
Predicted vs. Reference – emi FWHM (nm)
Generated emi FWHM (nm)
Reference emi FWHM (nm)
90
80
70
60
50
40
30
30
40
50
60
70
80
90

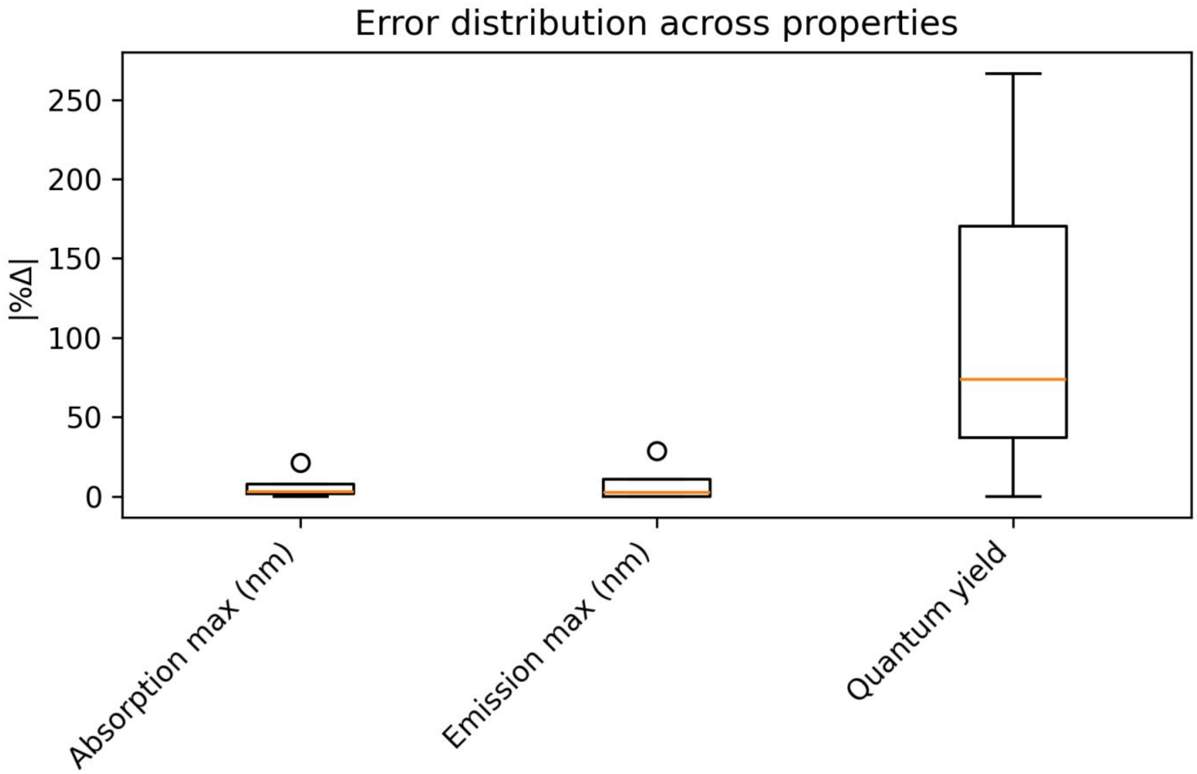
Error distribution across properties
250
200
150
100
50
0
|%Δ|
Absorption max (nm)
Emission max (nm)
Quantum yield

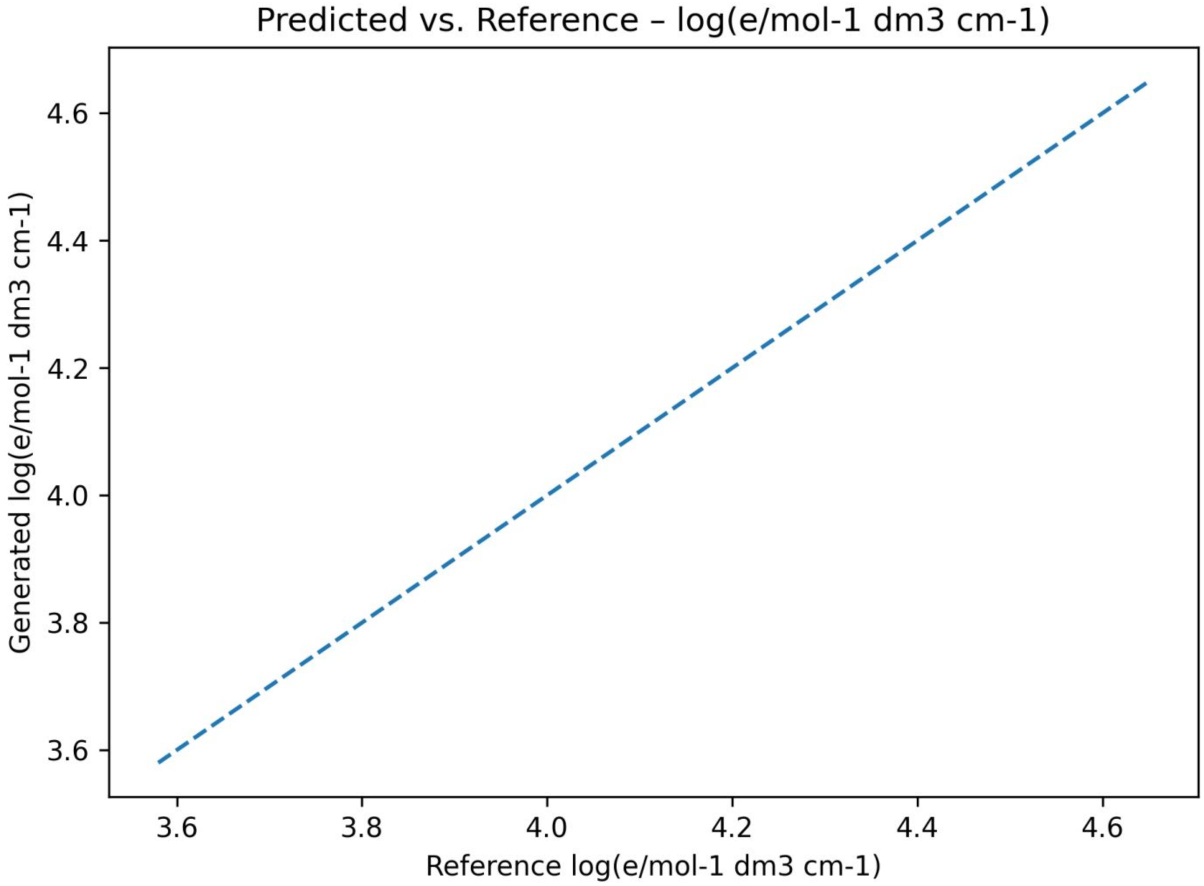
Predicted vs. Reference – log(e/mol-1 dm3 cm-1)
Generated log(e/mol-1 dm3 cm-1)
4.6
4.4
4.2
4.0
3.8
3.6
3.6
3.8
4.0
4.2
4.4
4.6
Reference log(e/mol-1 dm3 cm-1)

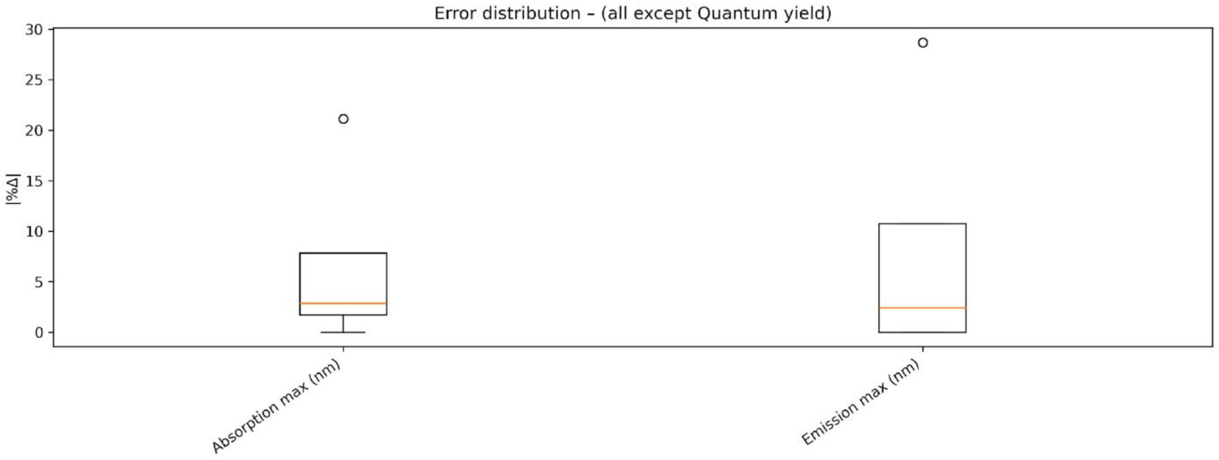
Error distribution – (all except Quantum yield)
|%Δ|
30
25
20
15
10
5
0
Absorption max (nm)
Emission max (nm)

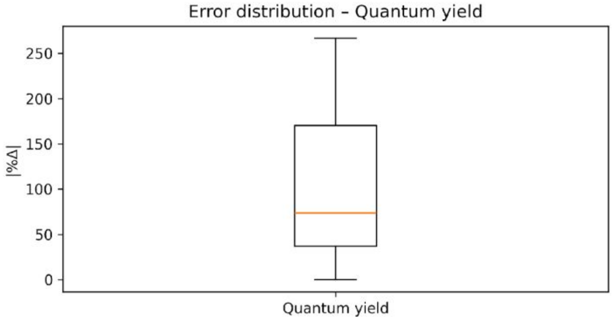
Error distribution – Quantum yield
|%Δ|
250
200
150
100
50
0
Quantum yield

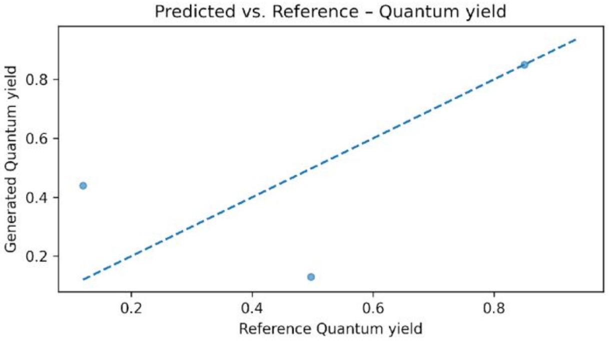
Predicted vs. Reference – Quantum yield
Generated Quantum yield
0.8
0.6
0.4
0.2
0.2
0.4
0.6
0.8
Reference Quantum yield
